# Supplementary material for: Monumental rock art illustrates that humans thrived in the Arabian Desert during the Pleistocene-Holocene transition
Source: Nat Commun. 2025 Sep 30;16:8249. doi: 10.1038/s41467-025-63417-y (PMC12485027; doi:10.1038/s41467-025-63417-y)
Supplement: Supplementary file 1 — Supplementary Infomation [file 41467_2025_63417_MOESM1_ESM.pdf]

Monumental rock art illustrates that humans thrived in the Arabian Desert during the Pleistocene-Holocene transition

Maria Guagnin*[1], Ceri Shipton*[2,3], Faisal Al-Jibreen [4], Giacomo Losi[5], Amir Kalifi[5], Simon J Armitage[6,7], Finn Stileman[8], Mathew Stewart[9], Fahad Al-Tamimi[4], Paul S. Breeze[10], Frans van Buchem[5], Nick Drake[10], Mohammed Al-Shamry[4], Ahmed Al-Shammari[4], Jaber Al-Wadani[4], Abdullah M. Alsharekh[11], Michael Petraglia[9,12, 13]

[1] Department of Archaeology, Max Planck Institute of Geoanthropology, Jena, Germany
[2] Institute of Archaeology, University College London, London, UK
[3] College of Asia and the Pacific, Australian National University, Canberra, Australia
[4] Heritage Commission, Ministry of Culture, Riyadh, Saudi Arabia
[5] Physical Science and Engineering Division, King Abdullah University of Science and Technology (KAUST), Thuwal, Saudi Arabia
[6] Department of Geography, Royal Holloway University of London, UK
[7] SFF Centre for Early Sapiens Behaviour (SapienCE), University of Bergen, Bergen, Norway
[8] Department of Archaeology, University of Cambridge, Cambridge, UK
[9] Australian Research Centre for Human Evolution, Griffith University, Brisbane, Australia
[10] Department of Geography, King's College London, London, UK
[11] Department of Archaeology, College of Tourism and Archaeology, King Saud University, Riyadh, Saudi Arabia
[12] Human Origins Program, Smithsonian Institution, Washington, D.C., USA
[13] School of Social Science, University of Queensland, Brisbane, Australia

*Corresponding authors

SUPPLEMENTARY NOTE 1: ROCK ART 3
SUPPLEMENTARY NOTE 2: JEBEL ARNAAN AND JEBEL MISMA EXCAVATIONS 13
SUPPLEMENTARY NOTE 3: JEBEL ARNAAN AND JEBEL MISMA ARTEFACTS 23
3.1. LITHICS 23
3.2. GRINDING, PERCUSSIVE, AND PLATTER STONES 35
3.3. PECKING AND SMOOTHING STONES 38
3.4. DECORATIVE ARTEFACTS 43
SUPPLEMENTARY NOTE 4: ARN AND JMI FAUNAL REMAINS 46
SUPPLEMENTARY NOTE 5: GEOLOGY, GEOMORPHOLOGY AND PALEOENVIRONMENT 47
SUPPLEMENTARY NOTE 6: CHRONOMETRIC DATING 56
6.1. LUMINESCENCE DATING 56
6.2. RADIOCARBON DATING 63
SUPPLEMENTARY REFERENCES 64

Supplementary Note 1: Rock art

Rock art was surveyed in three different areas along the southern edge of the Nefud desert: Jebel Arnaan (ARN), Jebel Misma (JMI) and Jebel Mleiha (MLH). Although the main body of rock art was similar in each area, with a majority of engravings belonging to a tradition of large, naturalistic animal engravings, we observed some differences in the in the stylistic, stratigraphic and thus chronological depth in each area.

At ARN, in the west of the project area, the mountain range rises to a height of over 400 m above the surrounding landscape, with steep but traversable slopes that are fringed with large boulders along the base, which provide surfaces for rock art production (Supplementary Figure 1). In the east of the project area, JMI is of similar height and forms a narrow mountain range with steep, impassable slopes and sheer cliffs. This contrasts with MLH, where rocky outcrops are smaller and more dispersed, reaching heights of ~200 m. This latter region contained few archaeological remains, while ARN and JMI were rich in archaeology.

1.1. Jebel Arnaan rock art (ARN)

At ARN, two panels had been reported (ARN1 and ARN3A, Supplementary Data 1) with sediments in front of both panels excavated by an amateur enthusiast wanting to expose buried sections of the panel. Archaeological surveys in the areas surrounding the site revealed surface lithics (Supplementary Note 3) and two trenches were excavated in front of panel ARN3A (Supplementary Note 2). Rock art surveys along the surrounding jebel slopes revealed a rich rock art landscape, and we recorded 46 panels with 64 life-sized animals (Supplementary Data 1).

Along the northern end of the embayment, large boulders obstruct the slope. A Saudi team member, Fahad Al-Tamimi (Heritage Commission) discovered an opening behind a large bolder which connects the embayment with a gully that runs down the slope from the top of the Arnaan mountains. Along this gully, water runs down the slope during the rainy season and pools in a rock pool (locally known as a “jubo”), where it can be accessed for months (Figure 8A and B). During excavations at the end of May 2023, water was still present despite the increasing heat and aridity of the season. Fresh animal footprints and faeces suggest this water is regularly accessed by wildlife. Of the 46 ancient rock art panels recorded at Jebel Arnaan, 7 were identified in wadi embayments, and 38 followed the water course to the top of Jebel Arnaan (Supplementary Figure 1).

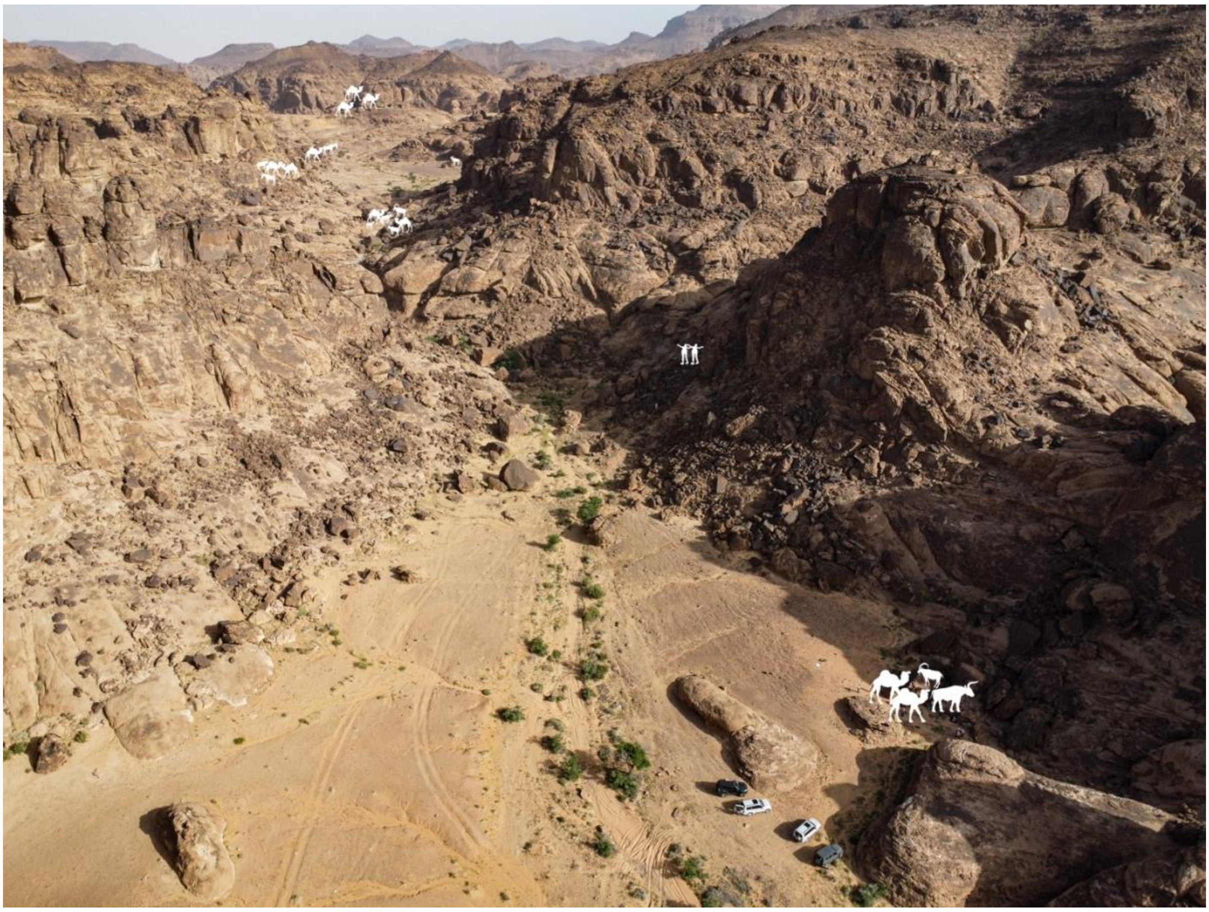

Supplementary Figure 1 Aerial view of the gulley marked with rock engravings at ARN. Excavations at ARN3 are visible in the front right. Icons of camels, ibex, equids, gazelles, cattle and human figures indicate the location and scale of life-sized engravings recorded along the gulley. Note that less than half of engraving panels are visible from this viewpoint, with additional panels obscured by rock spurs. Panels ARN44, 45 and 46 are visible in the far back. Vehicles in foreground for scale; scale of icons is approximate and optimized for visibility. Icons adapted from rock art.

Ancient rock art recorded at ARN matches similar life-sized and naturalistic representations of camels and equids that have been reported from Sahout[1], from Jebel Misma[2], and in high-relief from the Camel Site[3,4]. The engravings are generally life-sized or close to life-sized and are also highly naturalistic. Outlines always follow the animals' naturalistic shape and proportion; skin texture, skin folds and hair are frequently depicted alongside detailed representations of eyes, lips, nostrils and ears. Hooves, muscles and tendons are also generally shown with detail and precision (Supplementary Figure 2, Supplementary Figure 3). However, some engravings follow a more standardised representation of facial features or horns, perhaps reflecting a move from capturing naturalistic features of individual animals faithfully to a more standardised ideal of beauty (Supplementary Figure 4, Figure 2C, ibex).

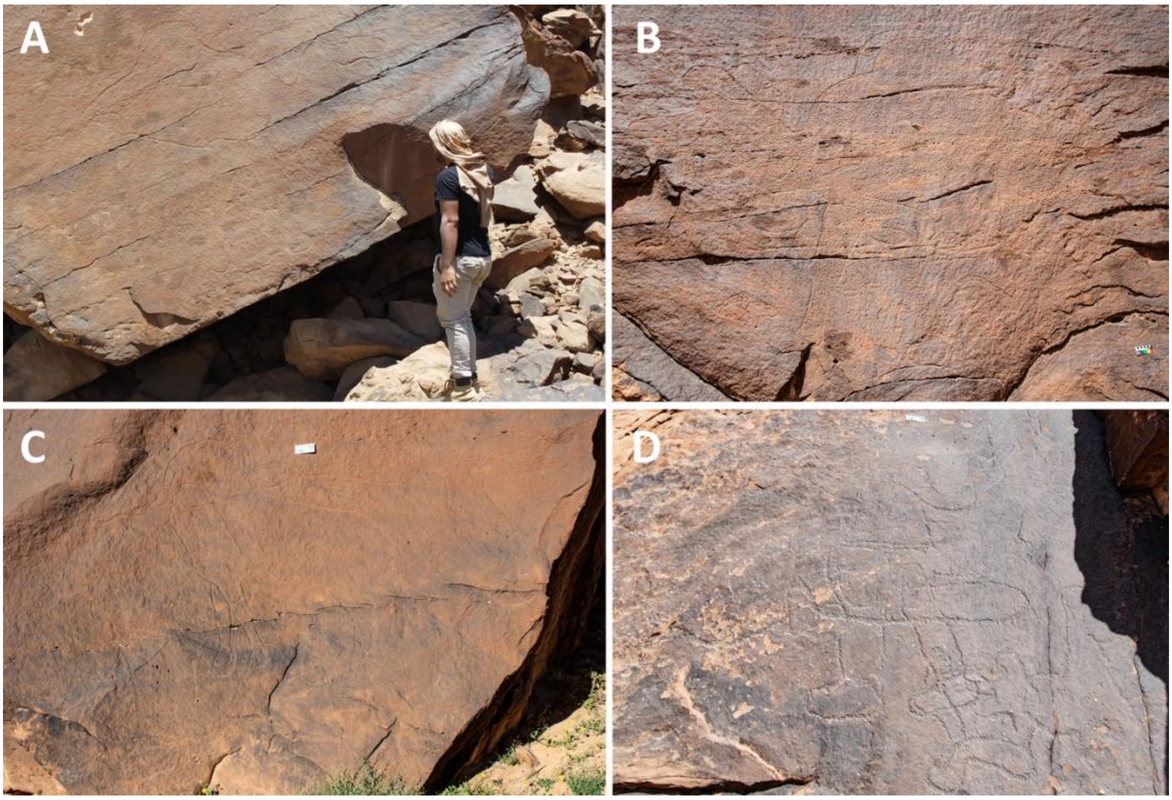

*Supplementary Figure 2 Large animal engravings from ARN. **A:** Panel ARN2 showing a life-sized camel, hind legs unfinished. Panel damaged by bullet holes, team member for scale. **B-D**: Untraced photos of panels ARN22A, ARN3B and ARN21A, shown with tracings in Figure 2. Scale 10 cm.*

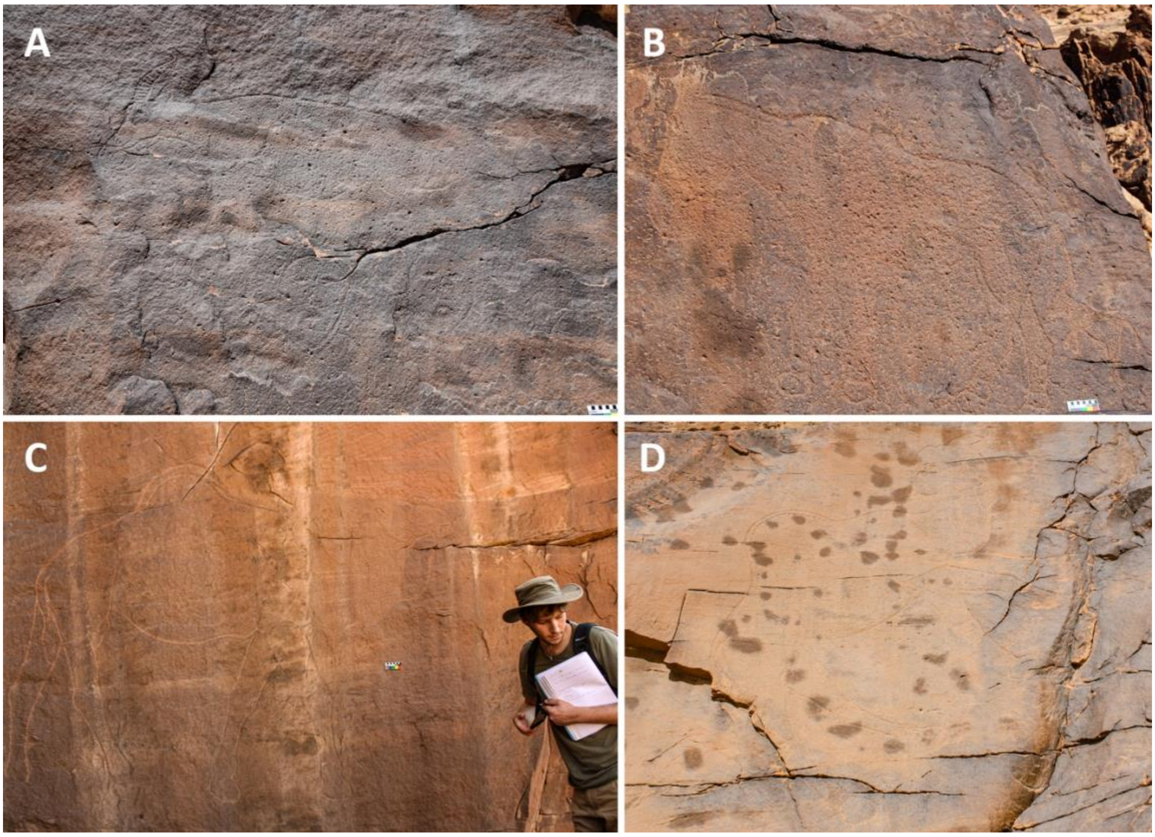

*Supplementary Figure 3 Large animal engravings recorded at ARN. **A:** ARN30, showing a gazelle superimposed over an equid. Below it two human faces or masks are visible in low relief. **B:** ARN39, showing an equid and its young, shown with tracing in Figure 2. **C:** ARN44 showing a large camel; team member for scale. **D:** Large camel apparently depicted walking up the panel slope. Note patches of moisture partly obscure this panel. Scale 10 cm.*

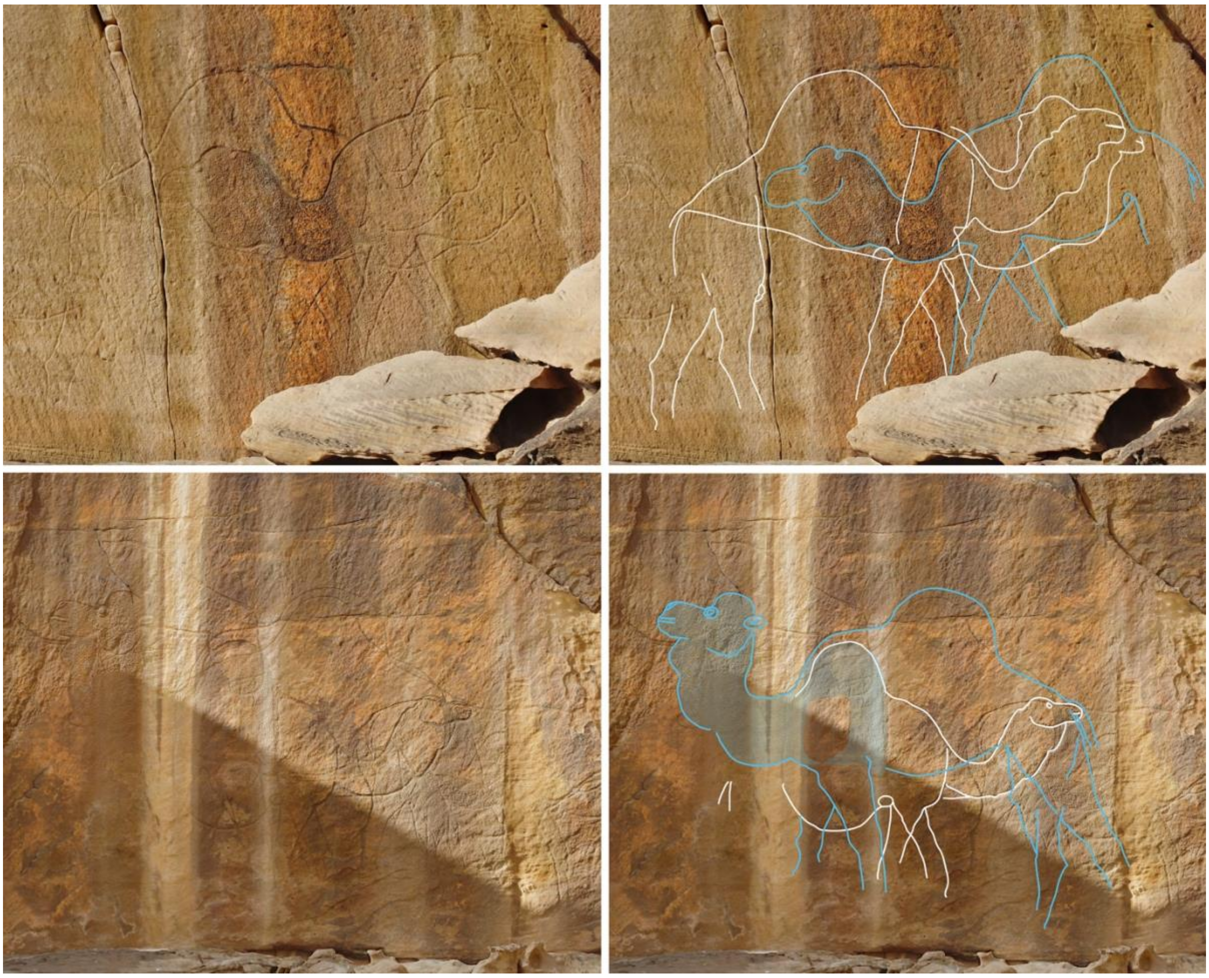

Supplementary Figure 4 Camel engraving sequence on panel JMI18. Top: Naturalistic and individualistic outline of a Phase 3 camel, traced in white. Superimposed with the more standardized head shape typical for Phase 4, traced in blue. Circular eye and pronounced jaw line are clearly visible. Bottom: Heavily stylised life-sized engraving of a camel with the round eyes, round muzzle and pronounced jaw typical for Phase 4 camels (traced in blue). Neck and shoulder pecked to give texture. Superimposed over an earlier camel with very small head and tapered neck (traced in white), typical of Phase 3. White traced camels: 1.7-1.9 m length, blue camels: 2.15-2.6 m length. Traced from an orthophoto generated from a high-resolution 3D model built from drone images.

Interestingly, no Neolithic rock art was recorded in the embayment and gulley at ARN (Supplementary Figure 5, top). The iconic rock art of the Neolithic is wide-spread across northern Saudi Arabia and is well known from multiple sites, including the UNESCO World Heritage sites of Jubbah and Shuwaymis[5-7]. Images from this period typically show stylised human figures in the so-called “Jubbah style” and stylised, medium sized representations of livestock and wildlife[8-10]. In this Neolithic rock art, individual animals are generally ca. 50-80cm in size, and are depicted with simplified outlines, lacking details such as eyes, nostrils or skin texture. At ARN, Neolithic rock art was only recorded in one location, ca 1.5km north of the main cluster of ancient rock art (Supplementary Figure 5, top). Remarkably, rock art panels in the embayment where ARN1 and ARN3 are located show exclusively Iron Age and Historic rock art, with the majority of these later panels showing human figures with firearms, providing a clear *terminus post quem*.

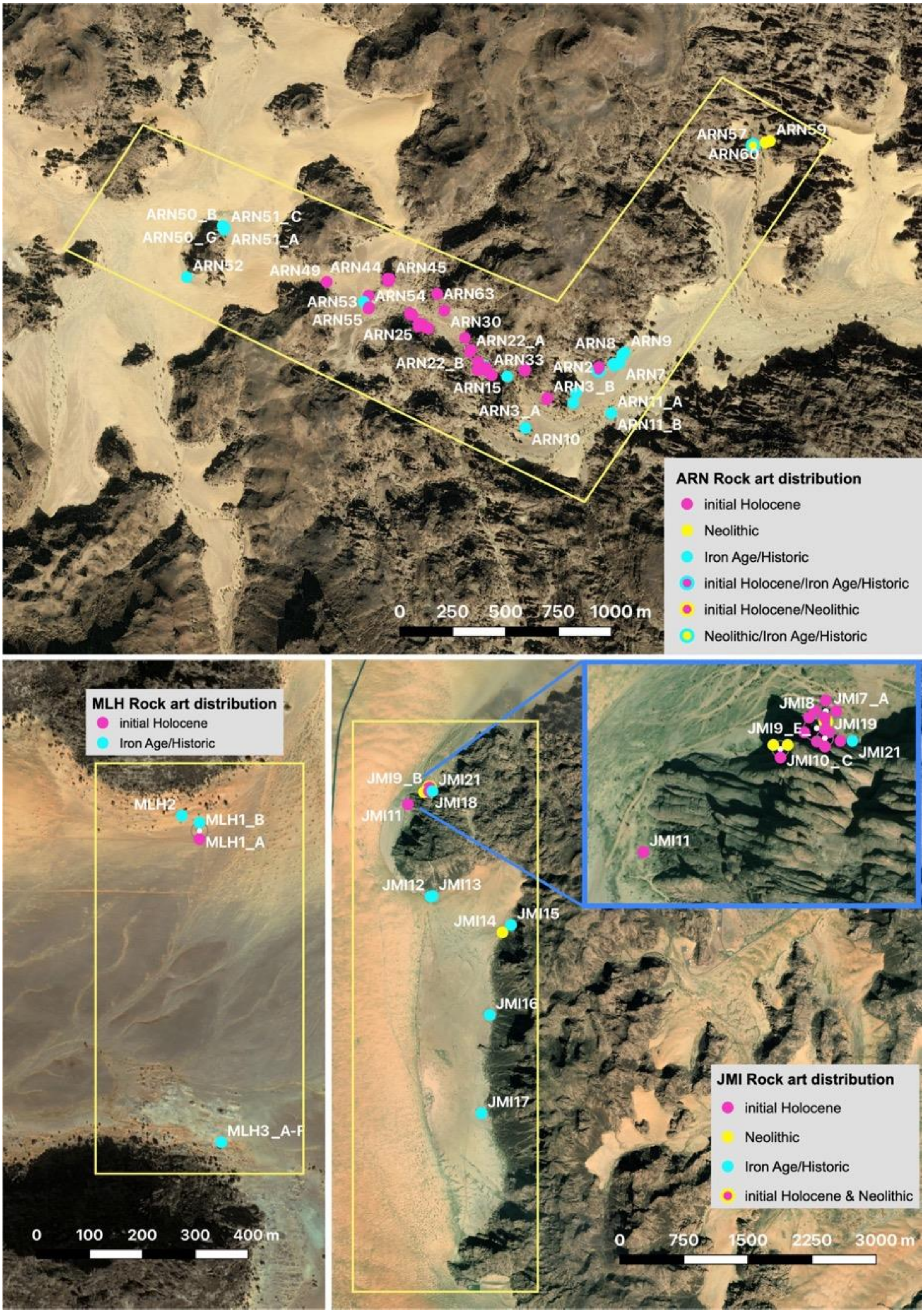

Supplementary Figure 5 Distribution of rock art panels by age. ***Top:*** *Distribution of rock art panels in Jebel Arnaan, showing a clear spatial differentiation.* ***Lower left:*** *at Jebel Mleiha rock art was only recorded on three boulders.* ***Lower right:*** *Distribution of rock art panels in Jebel Misma.* ***Inset*** *(blue frame) showing high-density locations using point displacement. Yellow rectangles indicate survey areas.*

At ARN life-sized, naturalistic engravings appear to span multiple engraving phases, and on some panels multiple stylistically similar engravings were found superimposed (Supplementary Figure 2B). In some cases, this phase of large naturalistic animal engravings overlies even earlier depictions, which generally depict human figures (Figure 2A; Supplementary Figure 2D). Re-use of the same rock surface suggests that the engraving tradition of the large naturalistic animal engravings was itself built on earlier rock art traditions in the region.

1.2. Jebel Misma rock art (JMI)

At JMI, three panels had previously been brought to the attention of the authors by Christoph Baumer and been documented during a brief visit in 2022[2]. Systematic surveys, carried out during fieldwork in 2023, identified 14 panels with 48 life-sized animals (Supplementary Data 1), including 23 on a single panel (JMI18, Figure 3, Supplementary Figure 4). Most panels are located in highly visible locations, facing a small embayment on the edge of a palaeolake. Freshly engraved, the engravings would have had a striking effect on the landscape (Supplementary Figure 6).

The distribution of the rock art at JMI shows slightly more spatial overlap with the Neolithic period (Supplementary Figure 5). Three panels with Neolithic rock art were recorded at JMI (Supplementary Data 1), in one case a Jubbah style human figure was apparently added onto the same panel as a life-sized, naturalistic camel (JMI7A)[2].

At Jebel Misma, erosion appears to be more advanced on cliff and boulder surfaces along the base of the jebel, particularly in the south-eastern end of the embayment. We surmise this may be a result of wind channelling along the cliff surfaces (Supplementary Figure 7). We also noted several heavily eroded footholds that had been carved into the cliff face to the east of JMI19 (Supplementary Figure 8). These provide access to a water hole (jubo) ca. 6 m above ground level, with further steps leading upward, towards panel JMI18. This spatial connection suggests the footholds are contemporary with the large, naturalistic rock art at JMI.

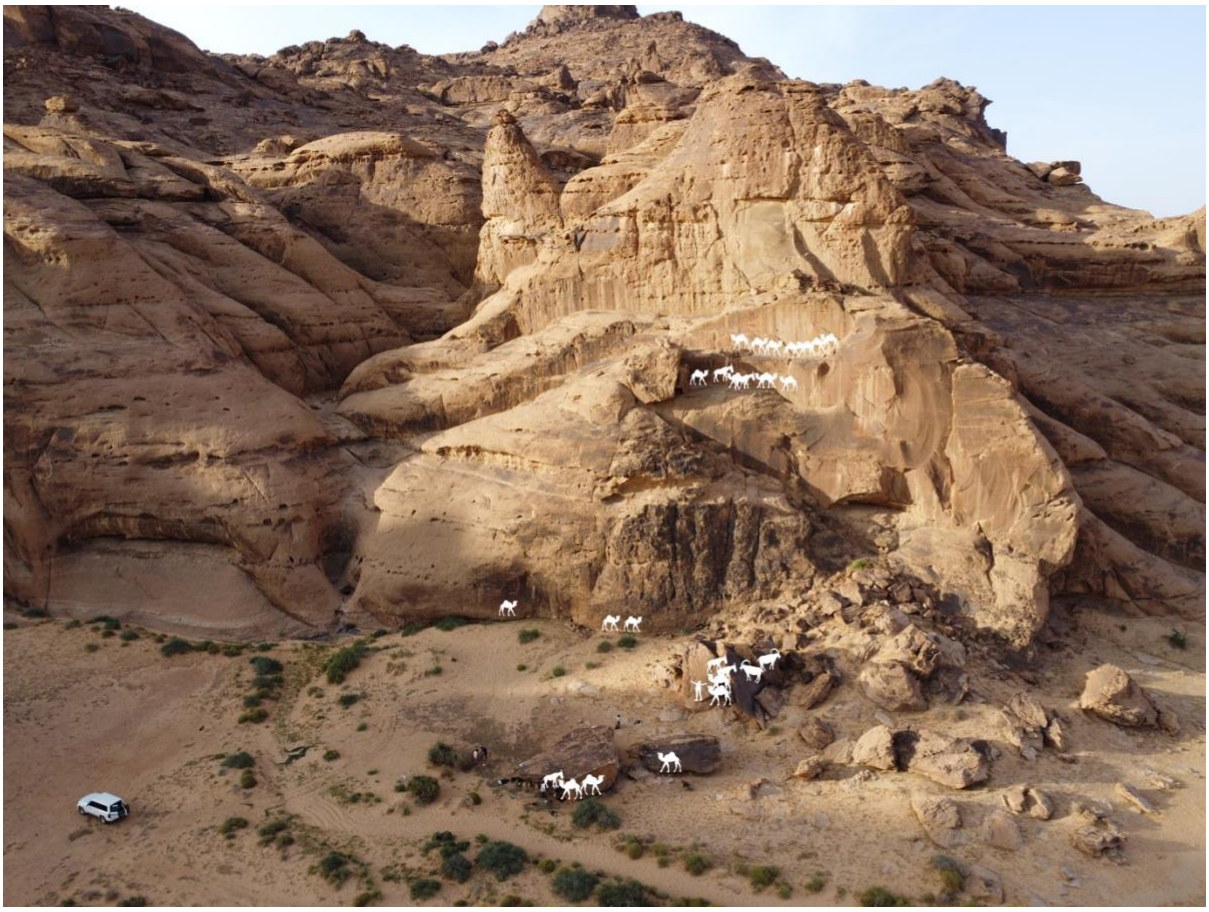
Supplementary Figure 6 Aerial photo of the JMI embayment, with icons of camels, ibex, equids and human figures indicating the approximate location and scale of the engravings. Excavations are visible in the centre foreground of the image. Vehicle for scale. Icons adapted from rock art.

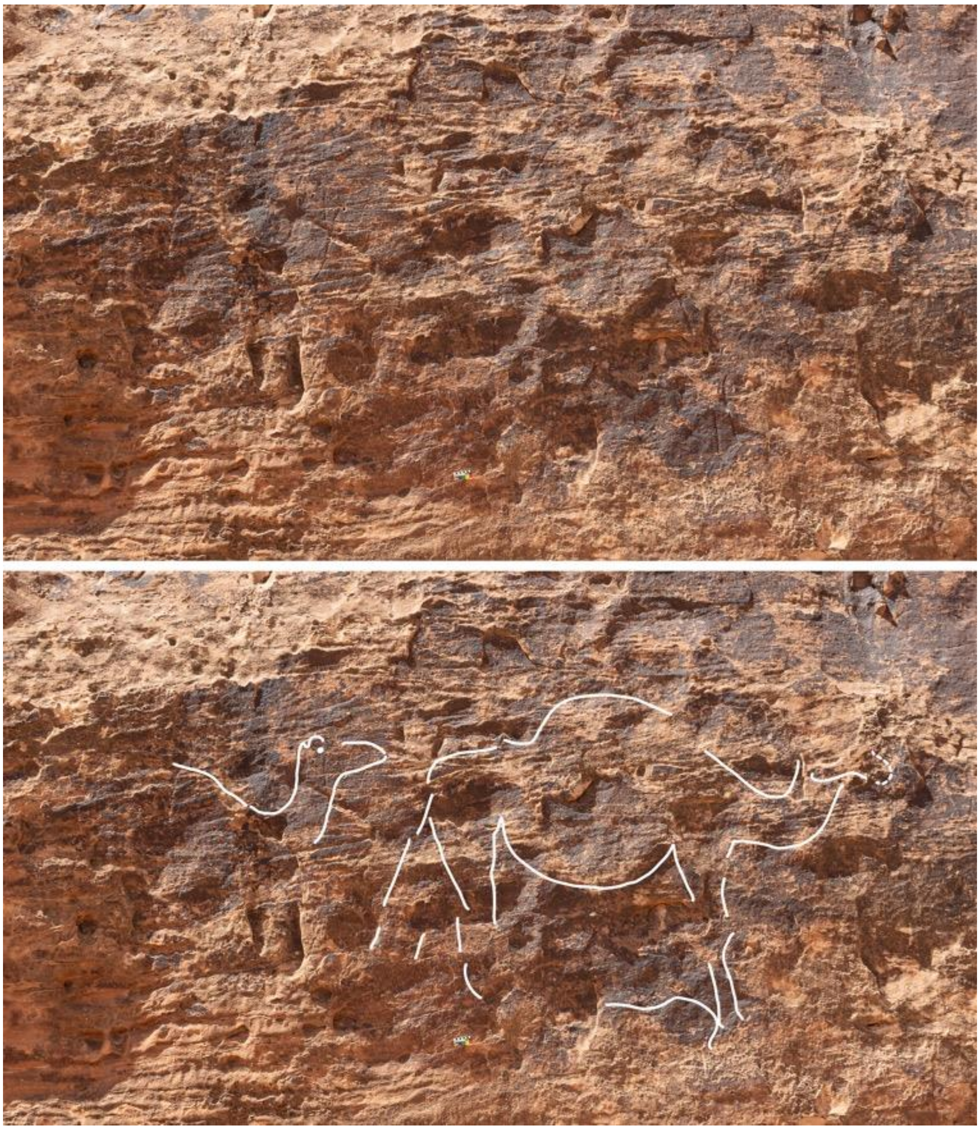

Supplementary Figure 7 Advanced erosion visible on Panel JMI19. This panel is located at the base of the cliff, in the south-eastern part of the embayment. Of the left camel only the neck and head remain.

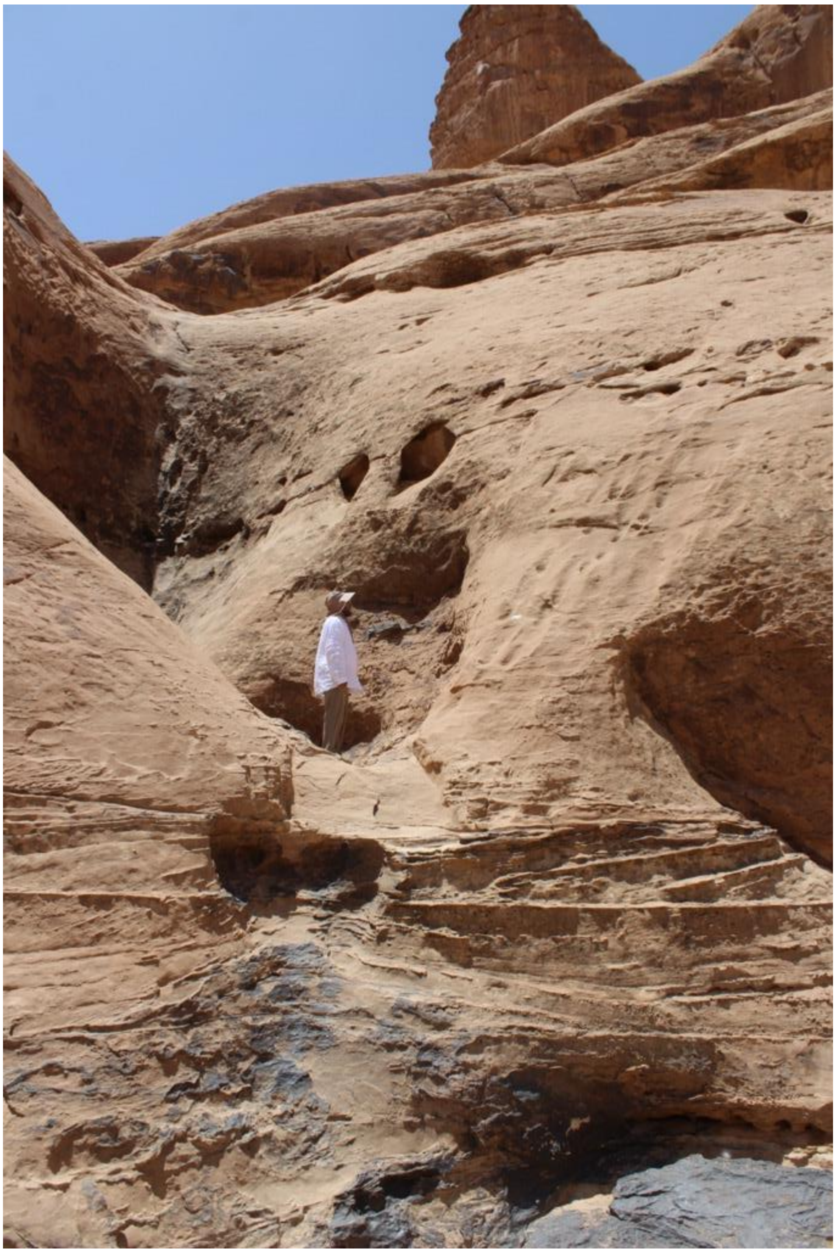

Supplementary Figure 8 Abdullah Alsharekh inspecting engraved footholds leading up to JMI18. A water hole opens up in the gap behind him and still contained water in May 2023.

1.3. Jebel Mleiha rock art

At Jebel Mleiha, only one engraving of a large, naturalistic animal was noted. The panel had been reported to the Heritage Commission by an amateur enthusiast, who had excavated sediments below the engraving to reveal the folded legs of a kneeling camel (Supplementary Figure 9). Only two other panels were noted in the immediate vicinity, with six further panels on the base of another outcrop, ca. 500 m south (Supplementary Figure 5). All other panels show Iron Age and historic engravings (Supplementary Data 1).

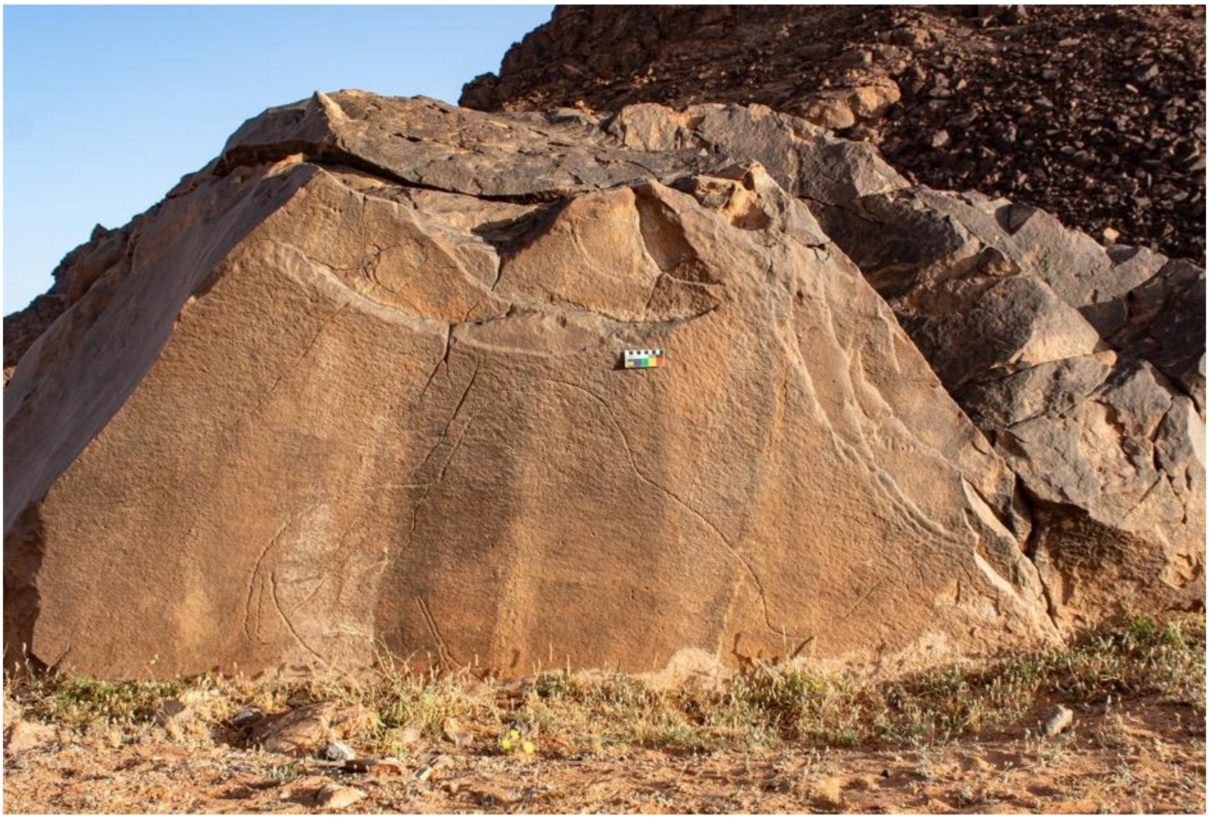

Supplementary Figure 9 Panel MLH1A, showing a kneeling panel. The sediment in front had been dug by an amateur enthusiast to a depth of ca 20 cm to expose the legs of the camel.

Supplementary Note 2: Jebel Arnaan and Jebel Misma Excavations

Archaeological deposits associated with rock art panels were excavated, with the aim of establishing a correlation between datable archaeological sites and rock art production. Only few rock art panels are surrounded by suitable substrate. Many of the panels at ARN and JMI are situated above bedrock, and at some sites sediments, and consequently archaeological remains, have been removed by amateur enthusiasts. However, we identified three rock art panels with archaeological deposits directly below the engraved surface. Two trenches were excavated in front of panel ARN3A, two trenches in front of JMI1B, and one trench in front of panel JMI8. Trenches were excavated using the single context method

2.1. ARN3, Trench 1

A 2x1 m trench was sited in front of the double engraved camel on the southwest side of the ARN3 boulder (Panel ARN3A), with the long side of the trench running parallel to the face of the panel ~15 cm from the rock face (Supplementary Figure 10). Disturbance from a rock art enthusiast was evident on the surface and had removed up to 0.5 m of sediment (Supplementary Figure 10; Figure 4), where he had dug up sediment to expose buried parts of the rock art panel. We placed the trench over the disturbance as we were interested in the association between any buried archaeological remains and the rock art panel. The trench was excavated to a maximum depth of ~180 cm below the current surface. The matrix throughout the excavation was a moist, friable, mid-brownish orange, well-sorted, medium sand, indicating principally aeolian input, with the moisture retention suggesting a silt component to the matrix.

Layers 1 and 2 were recent sand input into the hollow left by the disturbance.

Layer 3 represents further aeolian input into the disturbance hollow. In the lower half of this layer, there were frequent sub-angular pieces of sandstone and shale, as well as a few lithics, likely representing parts of the trench that were left undug during the disturbance and/or the immediate backfill of larger clasts.

Layer 4 had more frequent lithics, occasional small sub-angular sandstone and shale clast inclusions, as well as several larger sandstone rocks together with a large slab exposed on the northern side of the trench (Figure 4). This layer is therefore characterized by a large colluvial event, which sealed the layer below (Supplementary Figure 10). Photos taken by the amateur enthusiast, and passed on by Saudi colleagues, indicate also that this is below the level to which he dug.

The upper part of layer 5 had the highest artefact density and a moderate frequency of sub-angular sandstone and shale clasts up to ~5 cm in maximum dimension. The pecking stone was recovered from this layer in sediment under a large sandstone clast measuring approximately 60x60 cm in horizontal extent. This sandstone slab was itself sitting at the base of layer 4 on a triangular shaped rock that is still in the section (Supplementary Figure 10). The pecking stone was piece-plotted using a plumb-bob

and tape measures. Artefact density decreased in the lower half of layer 5, while sandstone and shale clasts increased to ~10 cm in maximum dimension.

Layer 6 was moister than previous layers with frequent sub-angular limestone and shale clasts and very few lithics. Excavation ended at this point as large sandstone slabs were covering much of the trench, impeding access to the diminishing area of sediment.

Two luminescence samples were taken from layer 5 to date the deposition of artefacts within this layer, including the pecking stone (Figure 4).

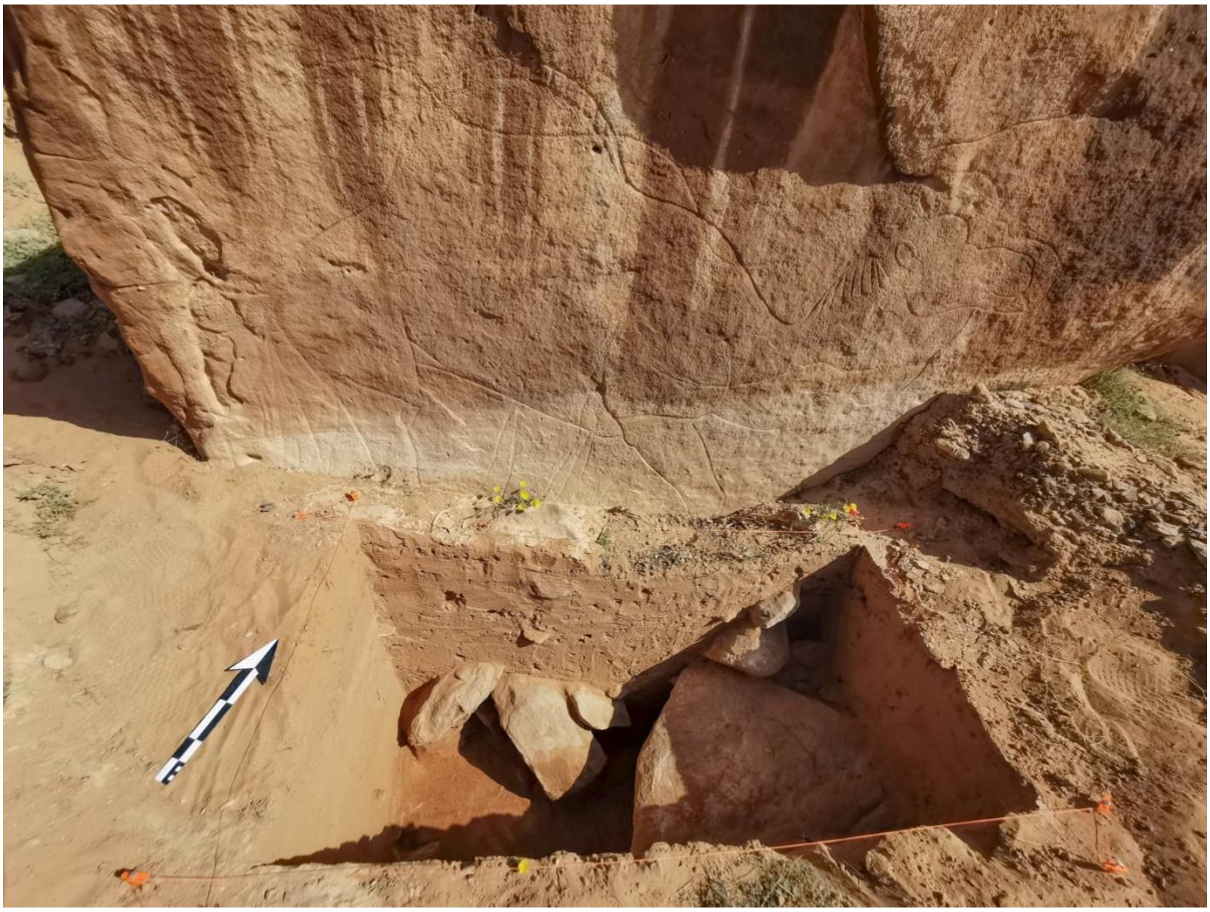

Supplementary Figure 10 ARN3 T1 upper layer 5 at the end of excavation. The top of layer 5 is evident as the sediment stain across the large triangular rock in the north section. Note the relationship of the trench to the camel engravings and the height of the entire deposit prior to disturbance shown by the white horizon on the rock art panel. A 15 cm bulk was initially left in place to protect the engraving during excavation and to identify the stratigraphy of sediments directly in front of the panel. To ensure correlation between engraving and excavated layers this bulk was carefully removed at the end (see Supplementary Figure 11). Scale is 40 cm long.

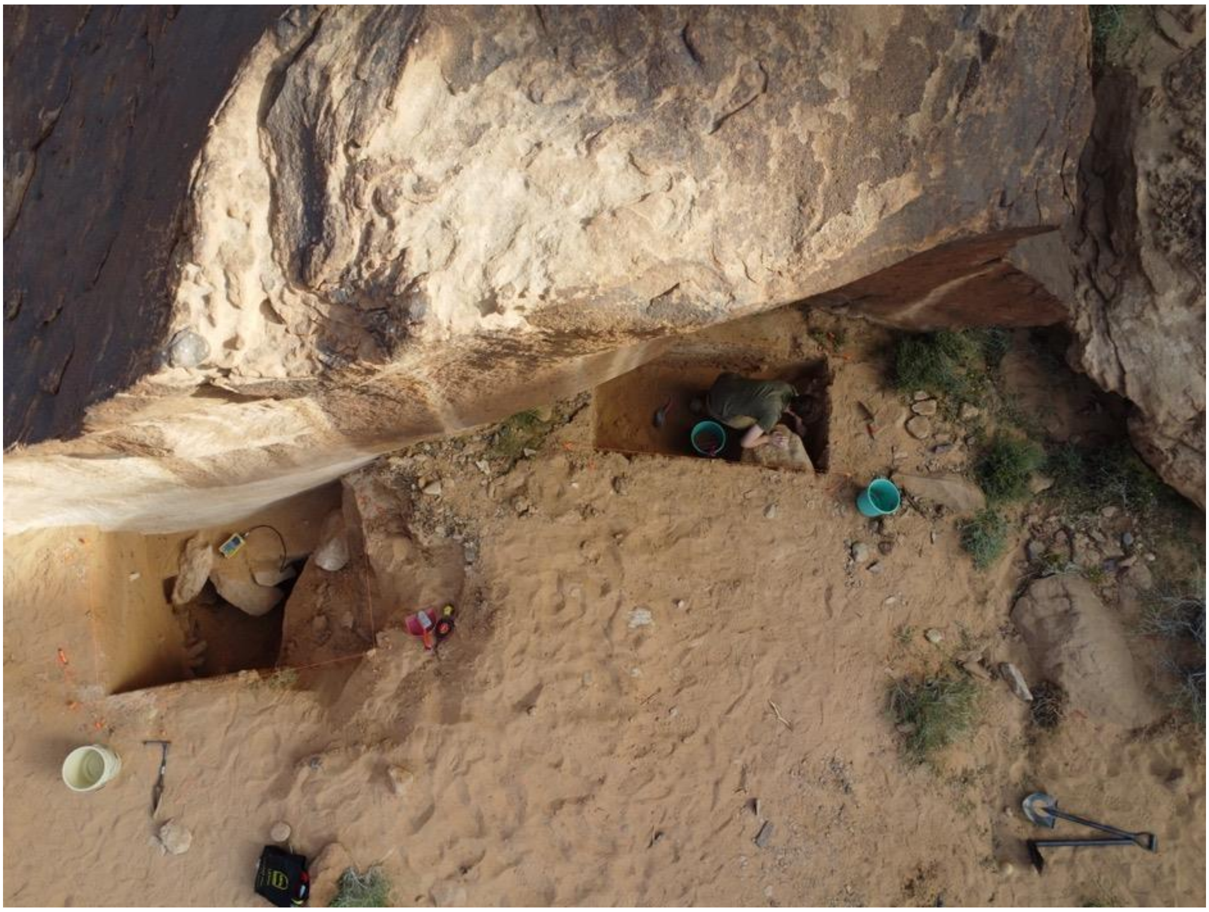

Supplementary Figure 11 Panel ARN3 viewed from above, during excavation. T1 on the left, with gamma spec measurements for luminescence dating in progress. Note that the gamma spec reader is resting where the bulk was removed as far as large sandstone blocks in layer 4 allowed. T2 on the right, with excavations in progress.

2.2. ARN3 Trench 2

Trench 2 was sited a few metres to the east of T1 (Supplementary Figure 11) on the same face of the boulder, but in front of a different petroglyph. Three partial/unfinished camels are engraved on this side of the panel, with one camel head visible above the ground surface (Supplementary Figure 12). A 2x1 m trench was placed in front of this panel, ~20 cm from the rock wall (Supplementary Figure 12). Ten layers were differentiated in the excavation, which reached a depth of ~120 cm (Supplementary Figure 13).

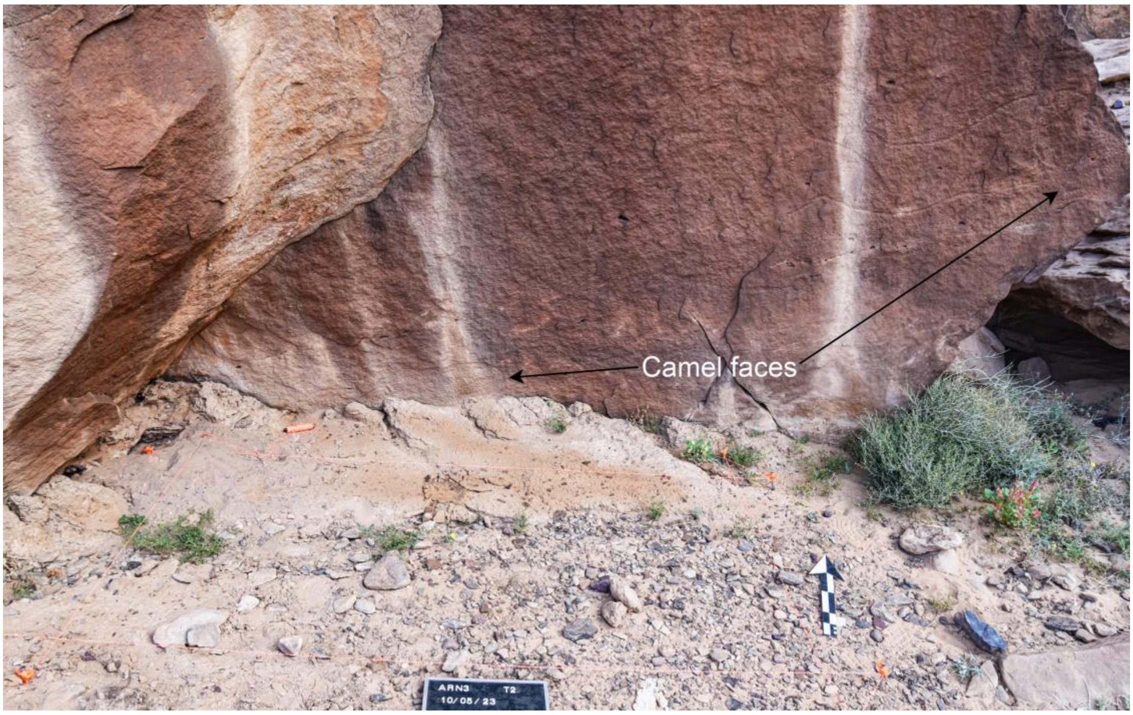

Supplementary Figure 12 The location of ARN3 T2 in front of a petroglyph panel with three partial camels on it. Note the gravelly sediment in the southern part of the trench and the finer sediment behind the boulder dripline in the northern part. Scale is 40 cm long.

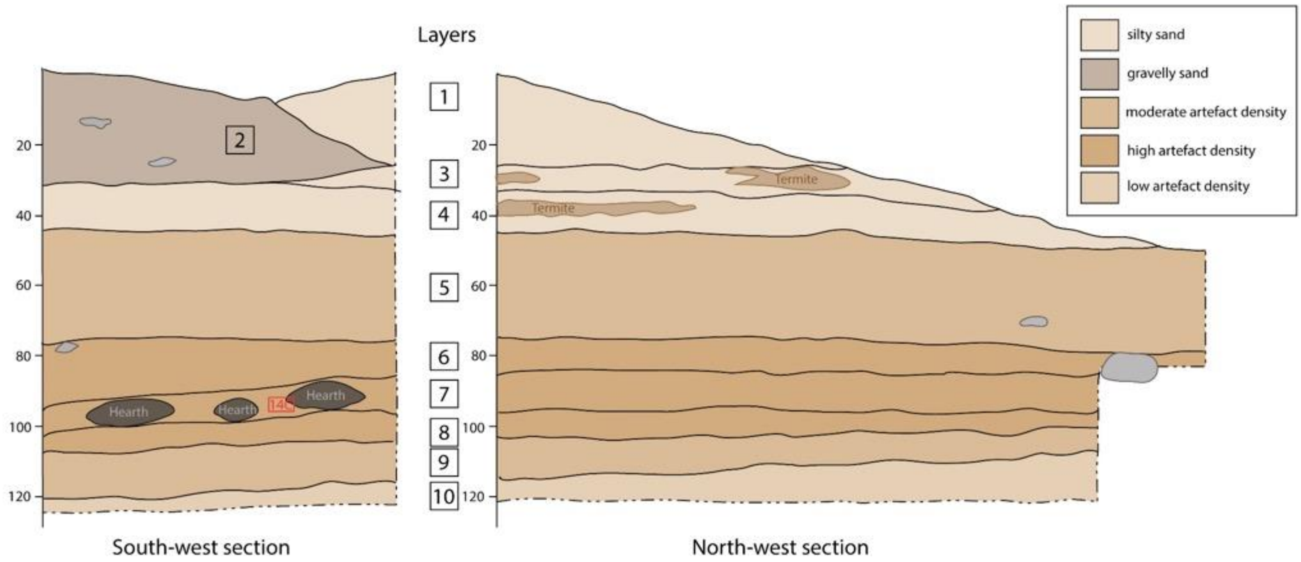

Supplementary Figure 13 Excavation profile of ARN3 T2.

Behind the dripline of the boulder, layer 1 was a very loose, dry, pale orangey beige, silty sand, with degrading camel dung and rodent burrows ~3 cm in diameter. On the outside of the dripline, layer 2 was a compact, dry, pale orangey beige, gravelly silty sand, with the gravel composed of small to medium sub-angular clasts of sandstone and shale.

There was a sharp contact between layers 1 and 2 and layer 3 below, the latter occurring across the whole trench. Layer 3 was a dry, loose, pale beigey orange, silty sand, with frequent small sub-angular sandstone and shale inclusions. The well-sorted nature of the sand likely reflects aeolian deposition. Layer 4 was a dry, loose, pale

beigey orange silty sand with occasional sub-angular sandstone and shale inclusions. Termite nesting was noted in layers 3 and 4.

Layer 5 was the first layer to produce significant quantities of lithics. It was again a dry, loose, pale beigey orange, silty sand, with occasional sub-angular sandstone and shale inclusions.

Layer 6 produced a high density of lithics in a sediment matrix of dry, loose, pale beigey orange, fine sand, with a moderate frequency of small to medium sub-angular sandstone and shale clasts.

Layer 7 continued the high density of lithics of layer 6 in a matrix of dry, loose, pale yellowish beige, fine sand, with occasional sandstone and shale inclusions. There were three ash lenses in the western end of the trench which went into the section, with a C14 sample collected from one of these (Supplementary Figure 13). Large horizontally bedded lithics were exposed at the base of this layer (Supplementary Figure 14). Layer 8 had a similar matrix to layer 7 and continued the high density of lithics, but had friable sandstone inclusions.

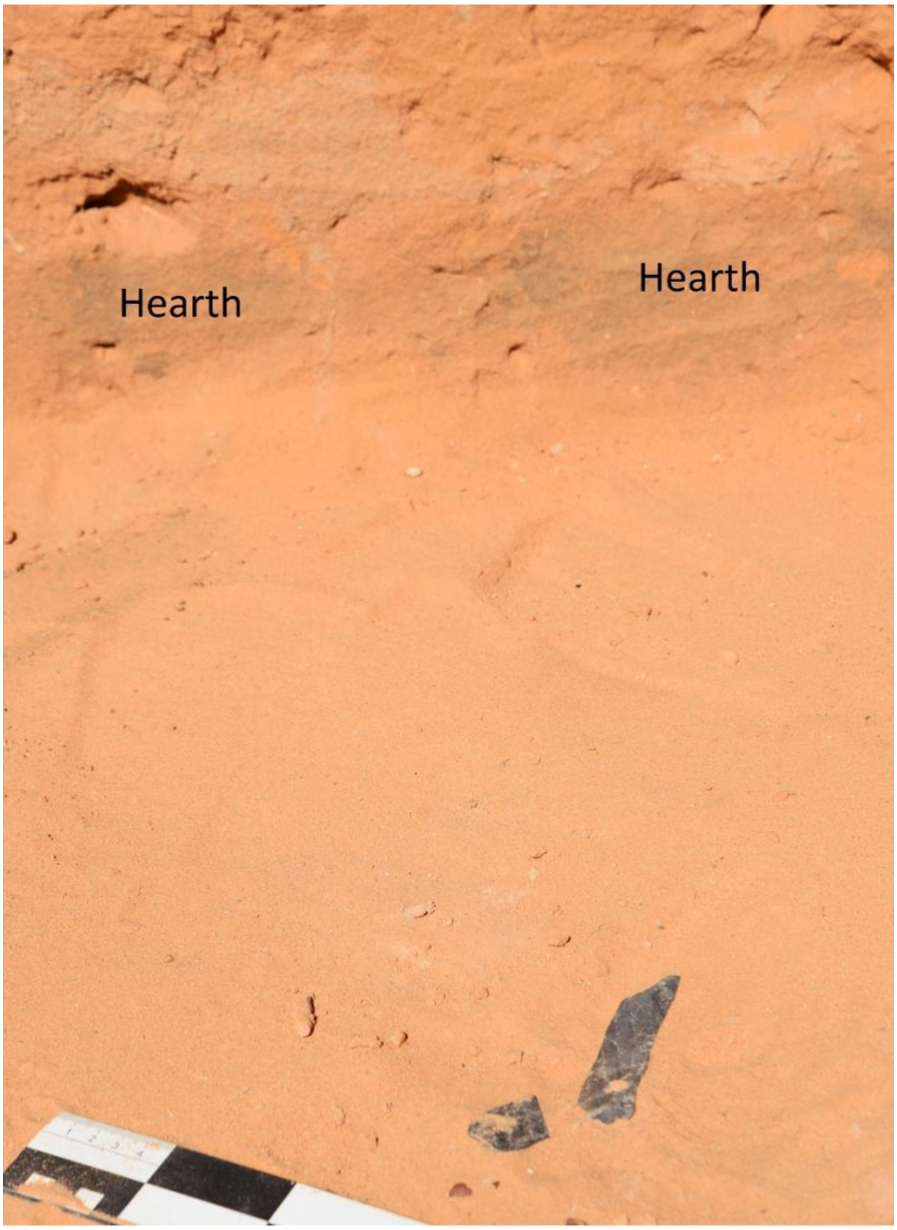

Supplementary Figure 14 The top of ARN3 T2 layer 8, showing the ash-lense hearths from layer 7 in the section and two large horizontally bedded lithics.

Layer 9 had a similar matrix to layers 7 and 8, the sandstone clasts were degraded and there were very few shale inclusions. Lithics dropped off in the lower half of this layer. A drip feature of calcrete precipitate, c.25 cm in diameter was noted in this layer and that below.

Layer 10 was a dry, loose, pale beigey orange, medium sand with very few lithics or other inclusions.

2.3. JMI7

JMI 7 is a small rock shelter with a rock art panel (JMI7B) featuring an equid and two camels (Supplementary Figure 15, Supplementary Data 1). The shelter provides shade from the late morning onwards. Caprine dung and shotgun shells were removed from the surface prior to excavation. The excavation was in two contiguous 1x1 m units (trenches 1 and 2), with the same sequence of 6 layers in each (Supplementary Figure

16). T2 was opened up in the thicker section on the east side of T1 to gain a larger sample of artefacts.

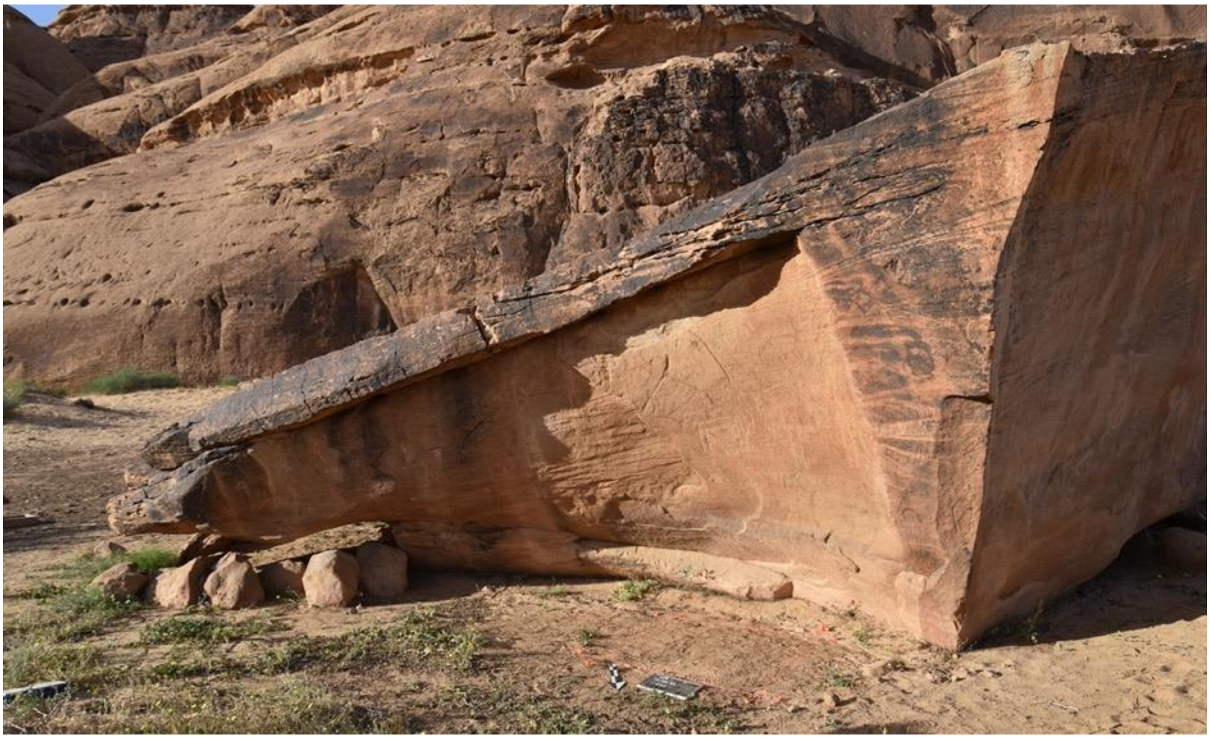

Supplementary Figure 15 Jebel Misma 7, with T1 laid out in the foreground.

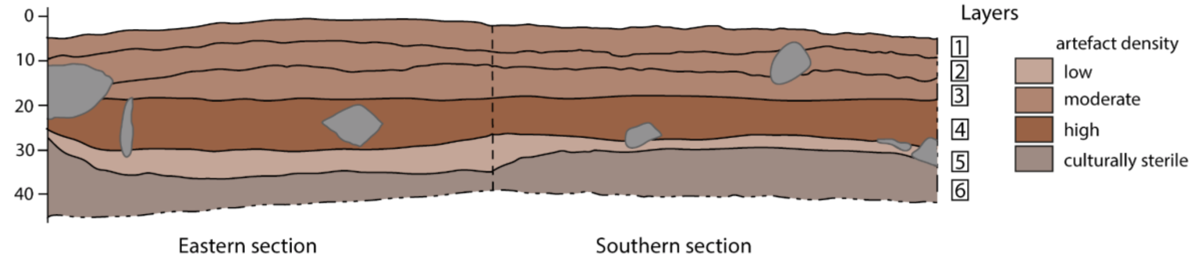

Supplementary Figure 16 JMI7 T1, eastern and southern sections. Note the eastern section is also the western section of T2. Note also the vertical clast on the left of the eastern section which is the platter that appeared to have been cached. A fragment of a pearl oyster was recovered from T1, Layer 3 and radiocarbon dated (Supplementary Table 7). Vertical scale in cm.

Layer 1 was a dry, compact, pale brownish beige, silty sand, with occasional lithics and shotgun pellets.

Layer 2 was a dry, loose, pale brownish orange, coarse sand, with sandstone inclusions.

Layer 3 was a dry, friable, pale yellowish orange, silty sand, with frequent small sub-angular shale and sandstone inclusions. A hearth lens was in the northeastern corner of this layer, with larger stones placed around it. A piece of pearl oyster was recovered from this layer and radiocarbon dated to 129-310AD (UGAMS69965; Supplementary Table 7).

Layer 4 was a dry, loose, mottled red and orange gravelly sand, with frequent large sandstone pieces up to 35 cm in maximum dimension. Two slabs of stone were recovered from this layer that appeared to be platters (Supplementary Note 3.2), with one vertically oriented as though it had been cached (Supplementary Figure 16).

Layer 5 was a moist, compact, brownish red, gravelly fine sand. There were some large clasts and a hearth lens in this layer. There was a sharp contact between this layer and the red silty fine sand of the layer below.

Layer 6 was only excavated in T1. This layer was a moist, compact, brownish red, silty fine sand, with no inclusions. No archaeological remains were found in this layer and a 10 cm deep sondage did not produce any further material so the trench was stopped at this point.

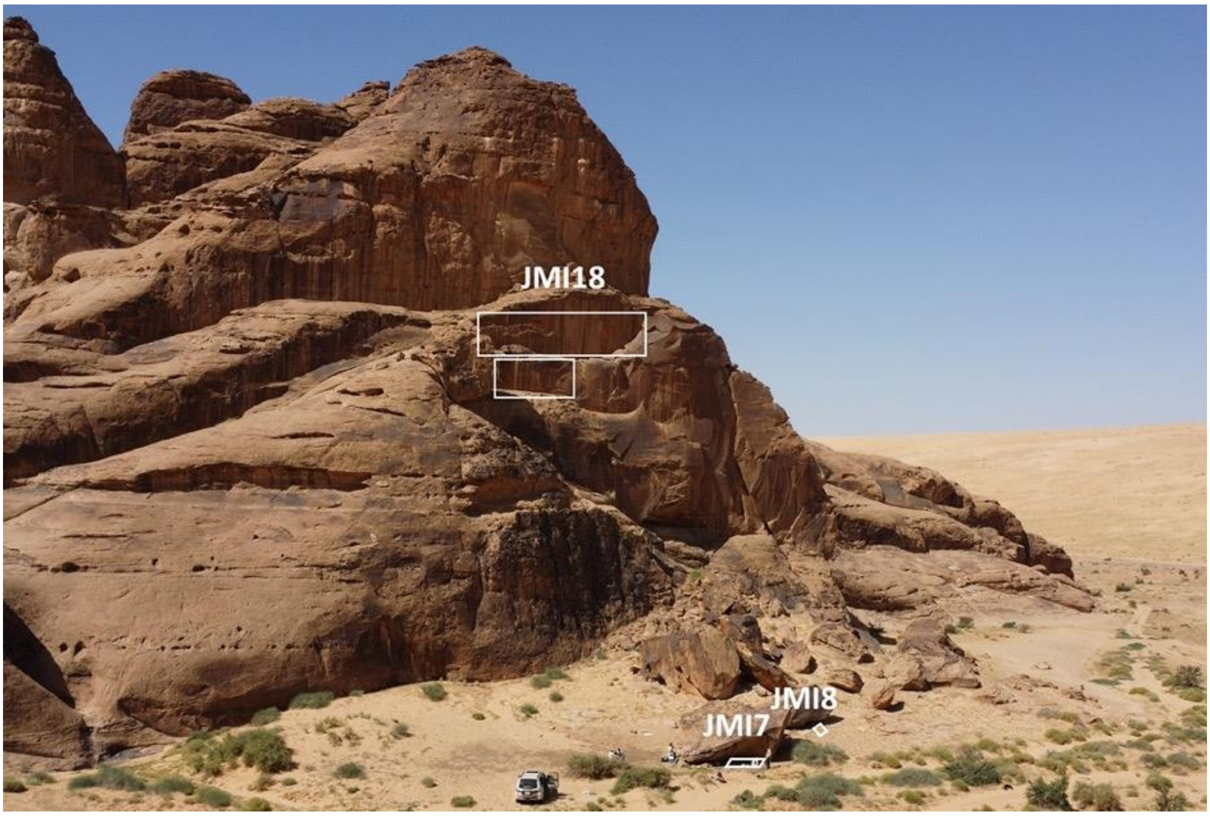

Supplementary Figure 17 Jebel Misma excavations and rock art. Aerial photo showing the location of JMI7 and JMI8 excavation trenches, next to boulders with rock art, and the location of the largest documented rock art panel (JMI18).

2.4. JMI8

JMI8 is a small boulder with an engraving of a large camel on it near to JMI7 (Supplementary Figure 17, Supplementary Figure 18). A putative silcrete pecking stone was found on the surface in front of the JMI8 boulder (Supplementary Note 3.3) along with quartz lithics. As JMI8 was higher up than JMI7 it was surmised there might be a greater depth of sediment above the basin floor. A 1x1 m trench was excavated to a depth of ~65 cm, within which 8 layers were differentiated (Supplementary Figure 19).

Layer 1 was a thin covering of dry, fine sand, likely from aeolian deposition, with lithics. Beneath this was a hearth with large charcoal fragments in the middle of the trench, measuring 43x39 cm horizontally and 7 cm thick.

Layer 2 was a dry, compact, pale orangey brown, sand, with occasional sandstone fragments. Layer 3 was a similar matrix but with more sandstone inclusions.

Layer 4 was a dry, compact, pale reddish brown, silty sand, with occasional sandstone inclusions. Larger clasts of sandstone extending beyond the walls of the trench were also exposed in this layer which continued down to the base of the trench.

Layer 5 was a dry, compact, pale orangey brown, silty sand, with frequent sandstone rubble inclusions. Layer 6 was a similar matrix to layer 5 but with fewer sandstone inclusions.

Layer 7 was a dry, compact, pale orangey brown, silty sand, with frequent small (up to 10 cm in maximum dimension) sub-angular sandstone fragments.

Layer 8 was a natural deposit of indurated, brownish red, silty fine sand, equivalent to layer 6 in JMI7, and was culturally sterile. The excavation area had become too small with the large clasts in the section walls by this point, so this deposit was not excavated.

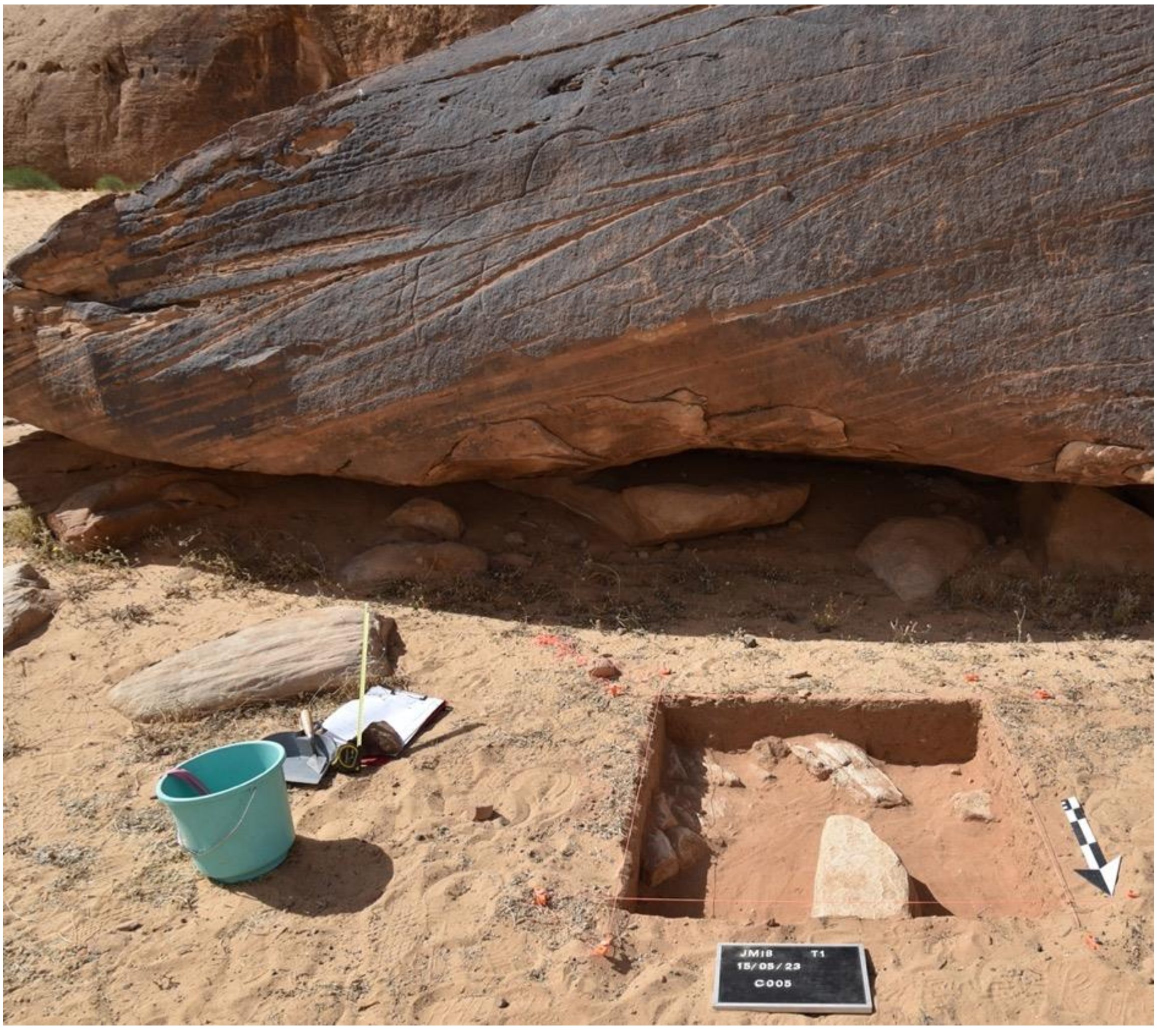

Supplementary Figure 18 The JMI8 excavation. Note the unfinished large naturalistic and fully varnished camel on the boulder behind. Scale is 40 cm long.

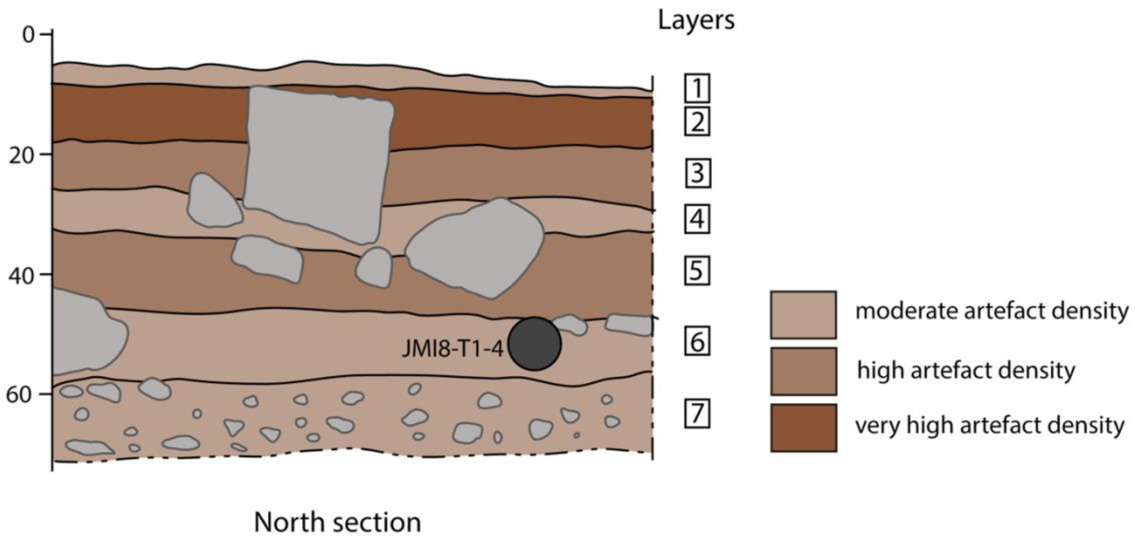

Supplementary Figure 19 The northern profile in the JMI 8 excavation.

Supplementary Note 3: Jebel Arnaan and Jebel Misma Artefacts

3.1. Lithics

3.1.1. Materials

Ten materials were used for knapping at ARN and JMI: a typical milky quartz, a fine crystal variety, and rarely a smoky crystal quartz. Chert came in a range of colours but typically orange; with a distinctive dark blue fine-grained variety producing the largest artefacts. The only igneous stone was a very fine-grained aquamarine banded obsidian or very fine-grained rhyolite. Silcrete, common in some layers, was typically peach in colour. Translucent brown and white chalcedony was a rare but persistent presence. Dark purple ferruginous quartzitic sandstone was used for large flakes while there was occasionally a pink true metamorphic quartzite. Sources for some of these have been identified in the surrounding landscape. Milky quartz occurs as small pebbles eroding out of Jebel Misma itself. A knapped silcrete outcrop was identified at Sahout (SAU4), north of Jebel Misma and Jebel Arnaan (Figure 1). Ferruginous quartzitic sandstone is widely available in the region, often around the base of jebels[11], including at the southern end of Jebel Misma; however we did not encounter any outcrops at Jebel Arnaan. The pink quartzite (known only from Jebel Misma) occurs as rounded cobbles in the Jebel Oraf Basin[12] around 100 km to the northeast, though it is possible there are nearer sources. The fine-grained volcanic stone (hereafter referred to as obsidian) seems to derive from the Jabal al Abyad volcano in the Khaybar region 180 km to the south[13] (Supplementary Figure 20). Sources for the chalcedony, the cherts, and the crystal and smoky quartzes are as yet unknown, but all are better represented at Jebel Arnaan than Jebel Misma.

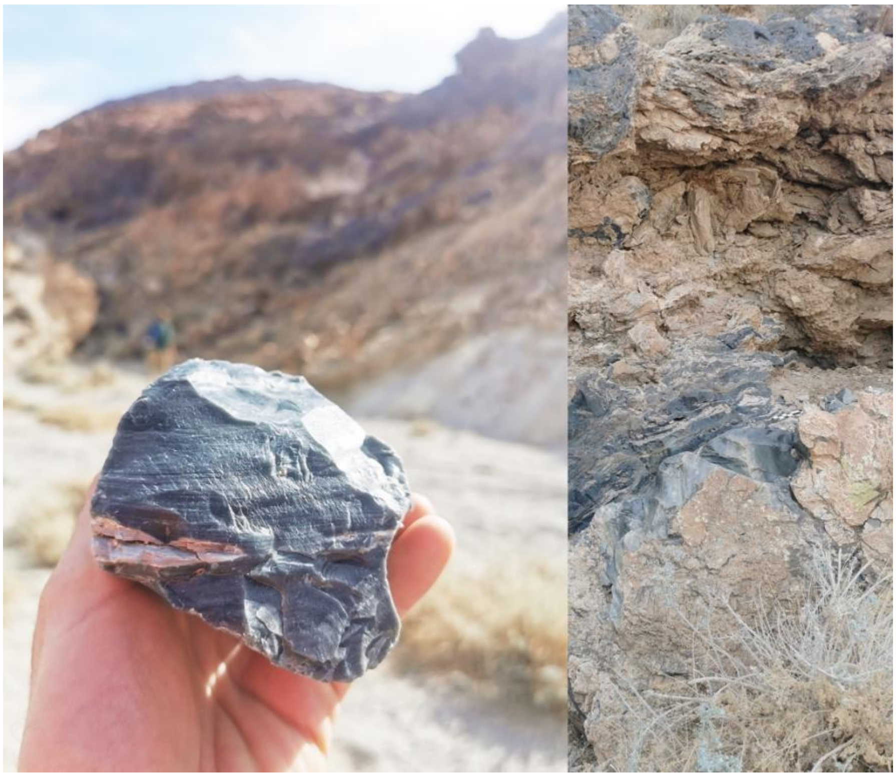

Supplementary Figure 20 An obsidian outcrop and knapped artefact at the base of the Jabal al Abyad volcano, Khaybar. Note the banding in the material

3.1.2. ARN

Two trenches were excavated at the ARN3 rock art site, with trench 1 (T1) producing 101 lithics and T2 producing 532. Six layers were differentiated in T1 with over half the lithics coming from layer 5 (Supplementary Data 2). In T2 large samples of lithics are represented across layers 5 to 9 (Supplementary Data 2) (only 7 lithics were recovered from layer 10 so these were combined with layer 9). Chert was the best represented material in both trenches, but there were also high numbers of obsidian and crystal quartz (Supplementary Data 2). The only discernible diachronic changes in material use were slight increases in ferruginous sandstone and milky quartz in the upper layers (Supplementary Data 2). A chi-square test comparing material distribution in T2 layers 1-5 versus layers 6-10, combining the two cherts and three quartzes, and with chalcedony and silcrete lumped together showed significant differences between the upper and lower sequence ($n=532$, $p<0.001$).

The T2 lithics were very small (mean weight 1.14 g, vs. 1.95 g for T1), suggesting *in situ* knapping and good recovery of debitage. A conjoin of an ancient break on a large dark chert blade from T2 layer 8 testifies to the low levels of spatial disturbance (Supplementary Figure 21). From T2 layer 8 obsidian complete flakes have a mean length of just 6.44 mm ($n=34$, $SD=3.07$), with some likely being from retouching. Indeed, the large obsidian retouched artefact from this layer had a larger maximum retouch scar length of 9.31 mm, and one of the obsidian flakes refitted on to this piece (Supplementary Figure 22). A five-piece refit sequence from layer 9, with an additional two-piece refit of the same distinctive brown veined chert, and a further 22 flakes of this

material from the same layer, all likely part of the same knapping event, testifies to the high integrity of the ARN sequence (Supplementary Figure 22).

The only cores represented in the excavated ARN assemblage were small bipolar pieces in chert, crystal quartz, and milky quartz. This strategy seems to have been used to maximise material productivity as both chert pieces were core-on-flakes and one was also used as a multiple notch, showing complex life histories. However, it is apparent that other knapping strategies were being used as there were several chert bladelets with lamellar scar patterns, as well as five chert, two crystal quartz, and one chalcedony piece with ground platforms. Most of the dark chert artefacts were large blades with parallel longitudinal arrisses and typically overhang removal (Supplementary Figure 21), with six of these also having ground platforms (Supplementary Figure 23); this unusual platform preparation strategy has also been documented at SAU on a dark chert blade [14].

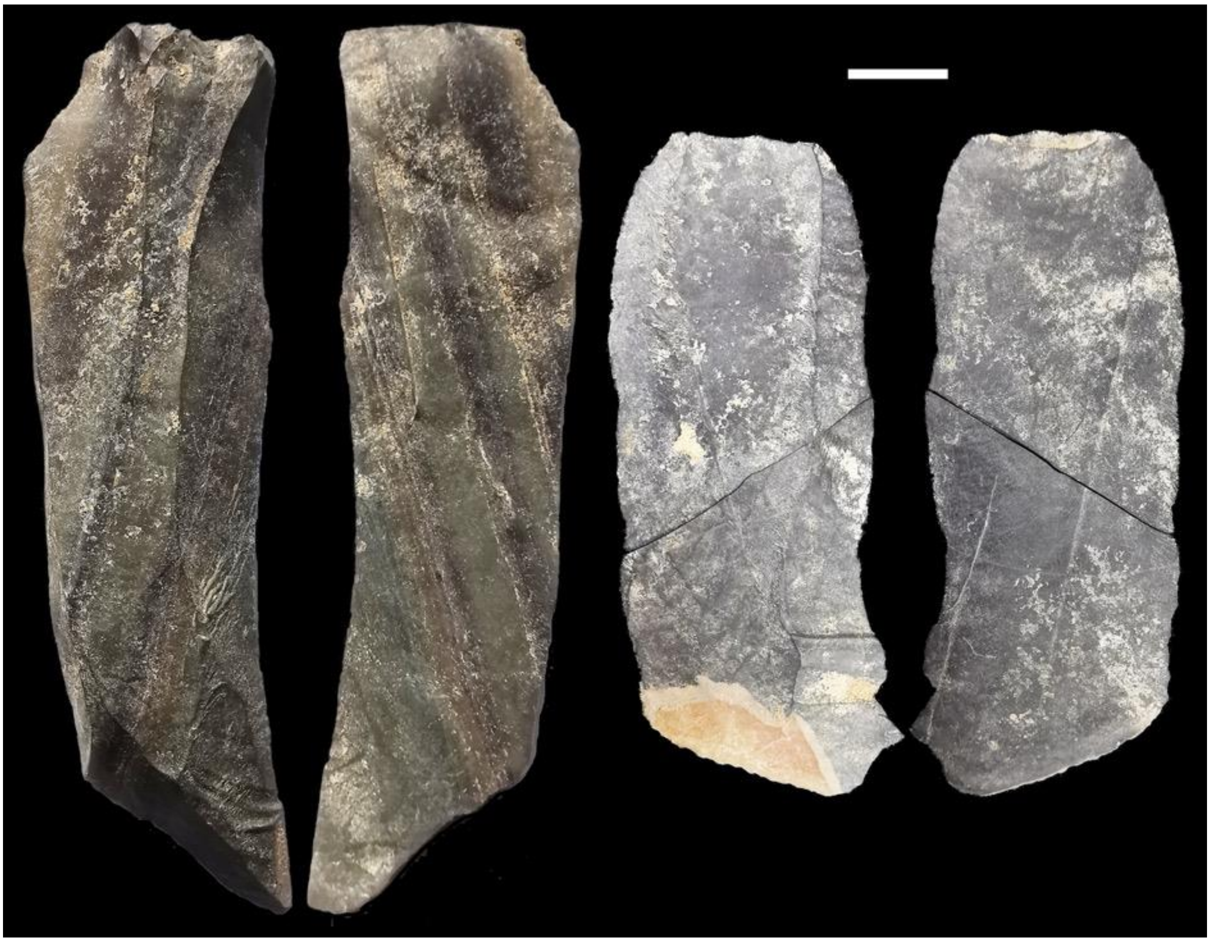

Supplementary Figure 21 Dark chert blades from ARN3 T2. Note that the blade on the right is a conjoin of an ancient break. The blade on the left is the longest flake in the assemblage at 87.26 mm. The blade on the left and the proximal part of the blade on the right were found together with a third blade in a cluster at the top of layer 8 (Supplementary Figure 14),. Both blades have parallel longitudinal arrisses, while the blade on the left has overhang removal. Scale is 1 cm long.

A total of 30 retouched artefacts were recovered from the ARN excavations (Supplementary Table 1). Notches were common, but particularly noteworthy were two distinctive opposed notch chert blades from T1. One of these was from the disturbance layers and had a ground platform, while the other was recovered from the stratigraphically secure layer 4 (Supplementary Figure 24, Supplementary Figure 25).

Multiple opposed notches on blades are characteristic of the Natufian in the southern Levant[15]. Perhaps also suggestive of the Natufian is a single crystal quartz Helwan bladelet from T1 layer 4 (Figure 5). However, the most diagnostic artefacts and those from the underlying main occupation phase in both trenches were three chert El Khiam points, indicating PPNA occupation, as also suggested by the dates from this trench. These were a broken butt from T1 layer 5, and both a broken butt and a complete El Khiam point from T2 layer 5 (Figure 5; Supplementary Figure 26). At under 3 cm long, the complete El Khiam point is akin to those from the southern rather than the northern Levant[16]. El Khiam points have previously been found at the surface site of Jebel Qattar 101, part of the Jubbah Oasis in the Nefud desert[17], but this is the first time PPNA artefacts have been found both in a stratified context in Arabia and south of the Nefud desert.

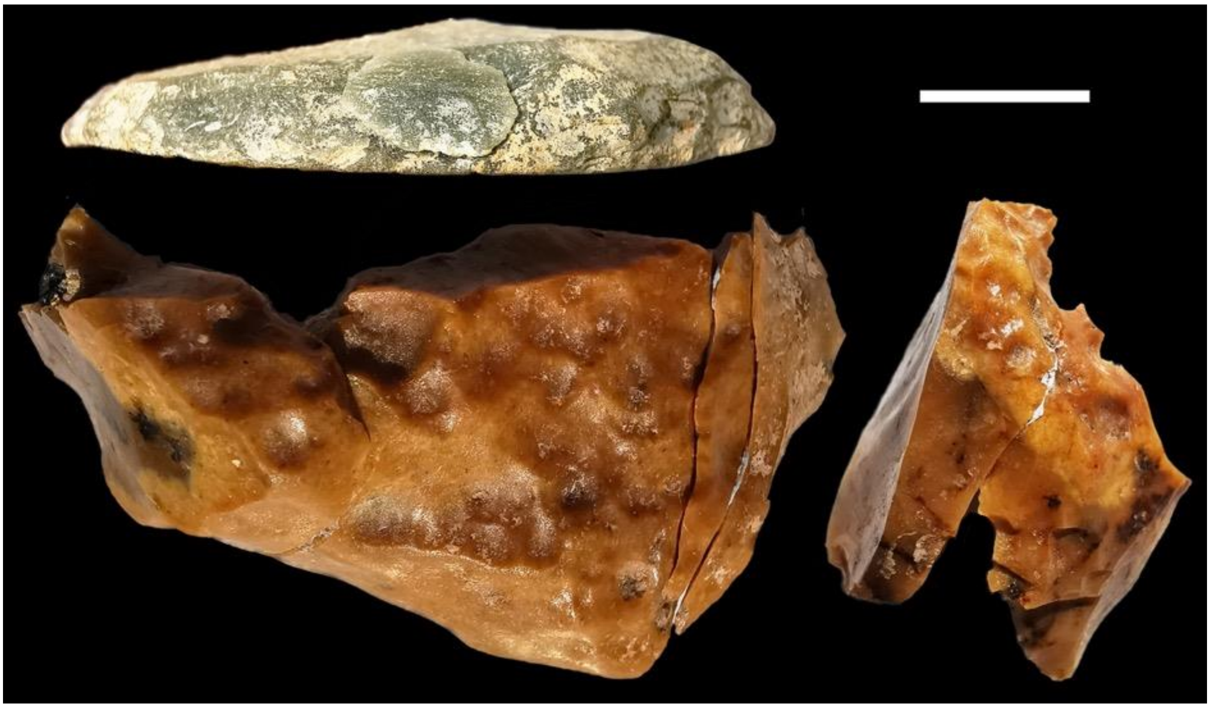

Supplementary Figure 22 Refits from ARN3 T2. Top refitting retouch flake on the obsidian scraper. Bottom refits of the same distinctive brown-veined chert. Scale is 1 cm long.

The most common type of points were bladelets with marginal distal convergent retouch (Supplementary Figure 24) (Supplementary Table 1), deriving from T2 layers 6-8. These were typically made on the dark chert, though there was one orange chert example (Supplementary Figure 25). The nearest parallels to these are Abu Maadi points of the southern Levantine PPNA[18], though the ARN examples lack equivalent butt modifications. The broken butt of an obsidian blade with a small tang recovered from T1 layer 5 (Supplementary Figure 26), may be an exception to this. Two larger blades also had marginal retouch, though these were not pointed; one was a dark chert piece from T2 layer 7, and the other was an obsidian piece with a broken distal end from T2 layer 8 (Supplementary Figure 27).

Two chert drills were found in T2 layers 8 and 9 (Supplementary Figure 24). The piece from layer 8 has abrupt dorsal retouch and that from layer 7 is a broken piece with

abrupt propellor retouch. They may be related to the manufacture of stone beads (see below), with drills a common component of the PPNA in the southern Levant[19].

A single endscraper was recovered from the T1 disturbance layers, and a scraper made on a broken flake was in T2 layer 7. However, the largest retouched artefacts were two tongue shaped scrapers from T2 layer 8 (Supplementary Figure 28). One of these was made of silcrete, a very unusual material in this layer (Supplementary Data 2), while the other was made of obsidian. We are not aware of any particular parallels to these pieces from the Levant.

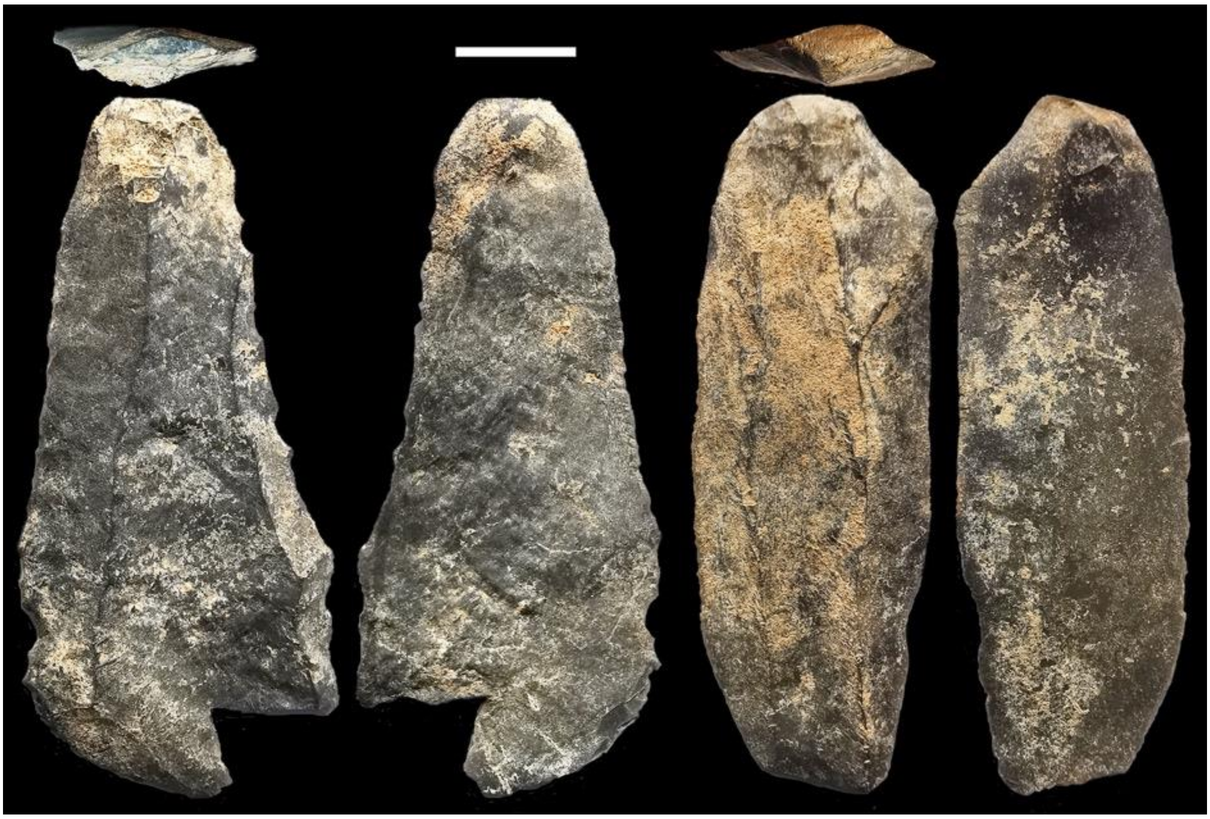

Supplementary Figure 23 Dark chert blades with longitudinal arrisses, overhang removal, and ground platforms from ARN3 T2 layer 9. Scale is 1 cm long.

Luminescence and radiocarbon ages obtained from T1 and T2 respectively combined with the presence of El Khiam points indicate that the main occupation phase in both trenches relates to the PPNA. Though there is overlap, the El Khiam points are underlain by the more idiosyncratic marginally retouched points in T2, with the tongue-shaped scrapers also occurring relatively early. The occurrence of a Helwan bladelet (Figure 5) adds to the initial identification of this artefact type at SAU2[14]. In addition to the excavated lithics, a small selection of diagnostic artefacts were documented on the gravel surface to the west of the ARN3 boulder; these included a silcrete naviform core (Supplementary Figure 29).

Supplementary Table 1 Breakdown of ARN retouched artefacts by trench.

**Retouched types**	**T1**	**T2**	**Total**
Notch	5	3	8
Opposed notch blade	2	0	2

Helwan bladelet	1	0	1
El Khiam point	1	2	3
Tanged point	1	0	1
Marginal pointed blade	0	7	7
Marginal	0	2	2
Drill	0	2	2
Endscraper	1	0	1
Tongue-shaped scraper	0	2	2
Other scraper	0	1	1
Total	11	19	30

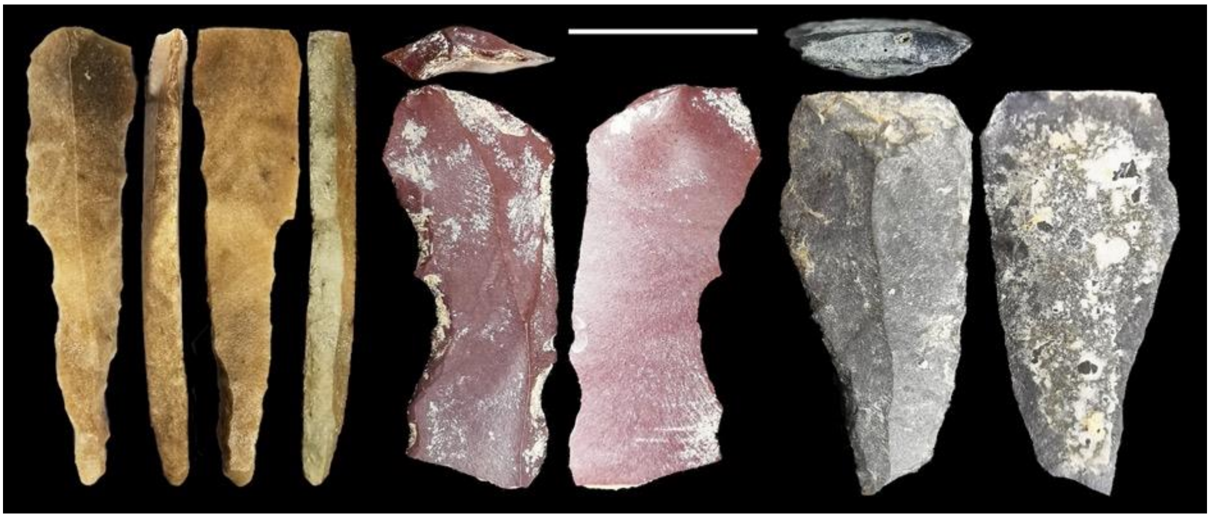

Supplementary Figure 24 Retouched bladelets from ARN3. Left is the chert drill from T2 layer 8, centre is the chert opposed notch blade from T1 layer 4, and right is a marginally retouched pointed bladelet with a ground platform and broken tip from ARN3 T2 layer 7. Scale is 1 cm long.

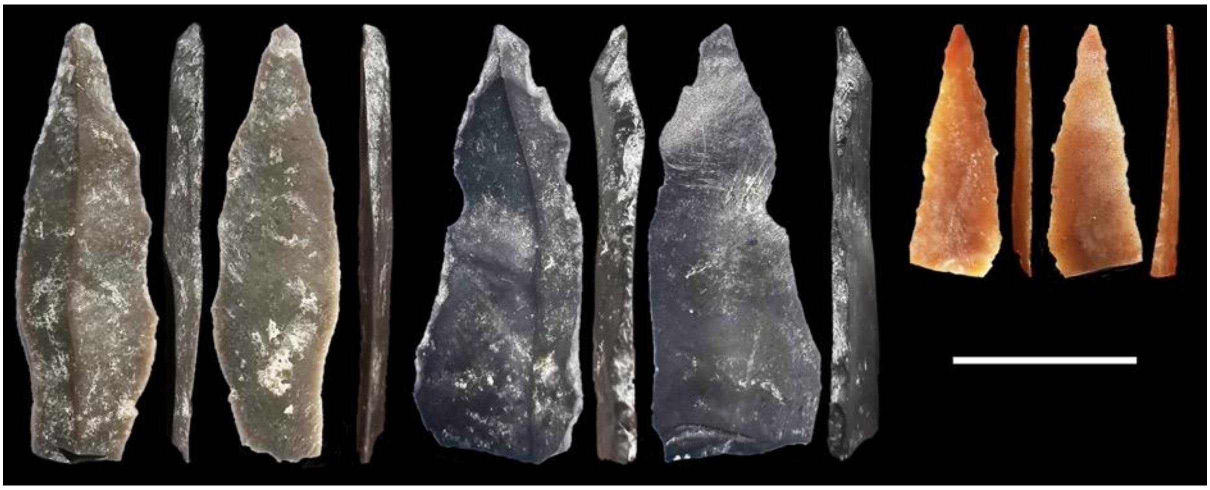

Supplementary Figure 25 Marginally retouched pointed bladelets from ARN3. Left is a dark chert piece from T2 layer 8, centre is a dark chert piece from T2 layer 6, right is an orange chert piece from T2 layer 6.

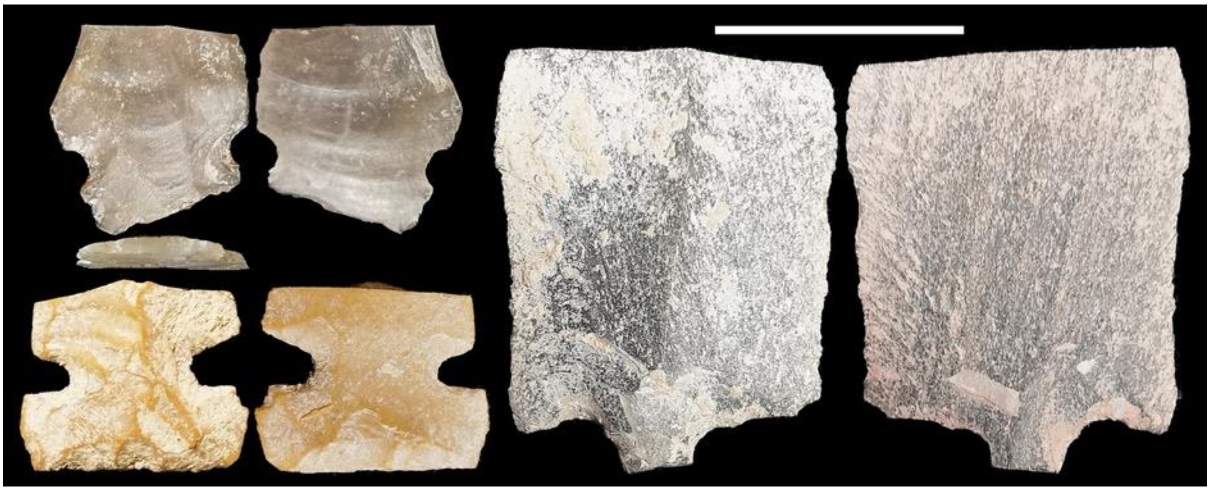

Supplementary Figure 26 Broken point butts from ARN3. Top left is the chalcedony butt of a perhaps unfinished El Khiam point from T2 layer 5 (the flaking on the butt is not invasive); bottom left is the chert butt of an El Khiam point from T1 layer 5; and right is the obsidian butt of an unknown point type from T1 layer 5. Note the banded shades in the material on the obsidian piece, equivalent to that in Supplementary Figure 20. Scale is 1 cm long.

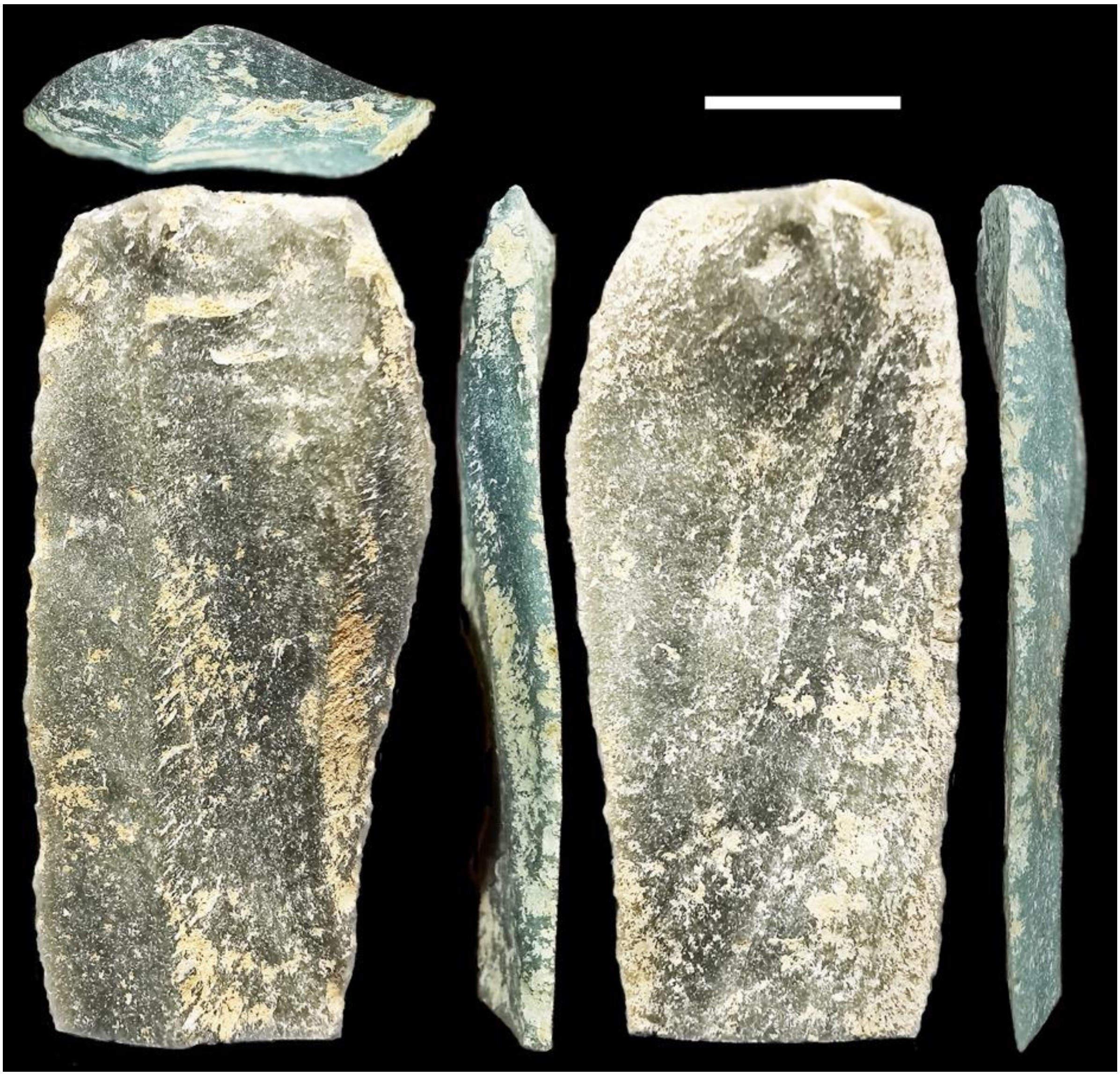

Supplementary Figure 27 Fine-grained obsidian blade with steep retouch along the entirety of both lateral edges and a broken distal end, from ARN3 T2 layer 7. The left facet of the dihedral platform has been ground. Note the banded shades in the material, equivalent to that shown in Supplementary Figure 20. Scale is 1 cm long.

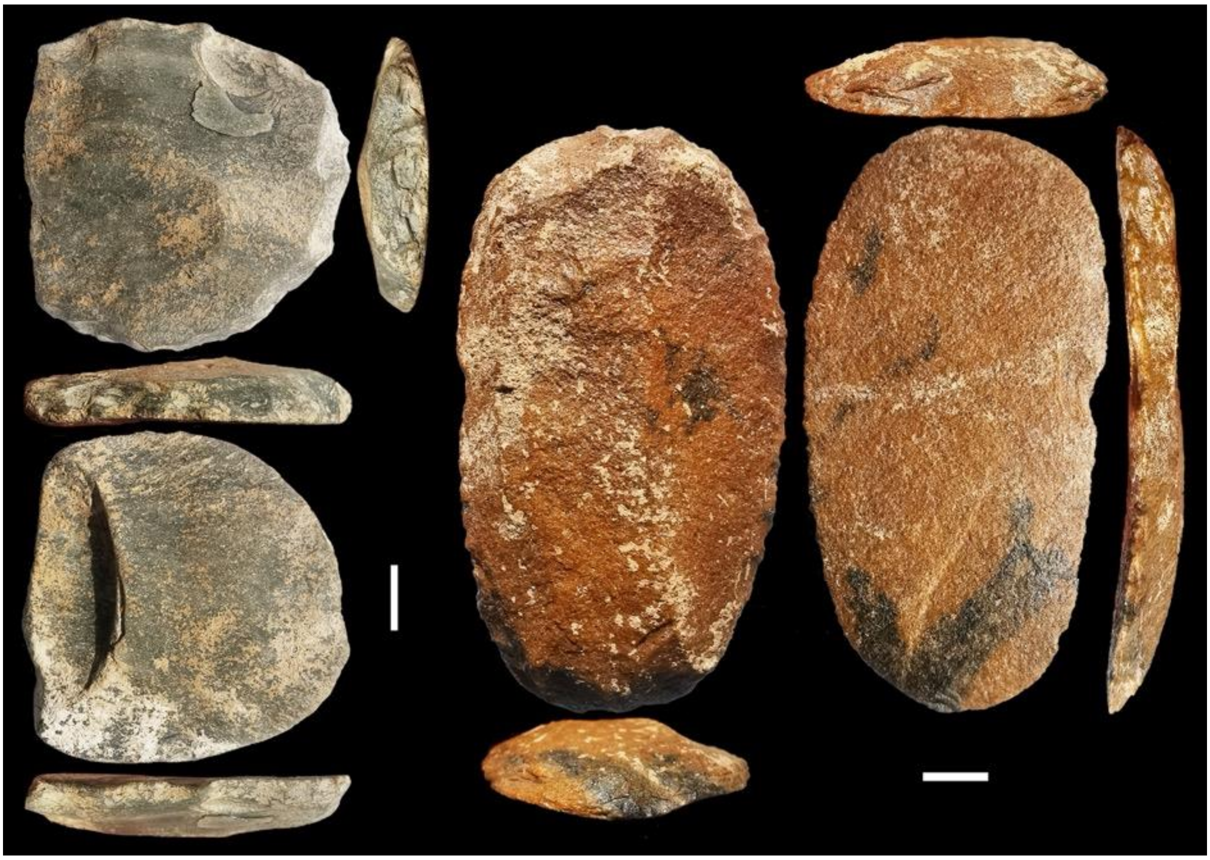

Supplementary Figure 28 'Tongue shaped' scrapers from ARN3 T2 layer 8. Left is a fine obsidian piece, right is a double-ended silcrete piece. Scales are 1 cm long.

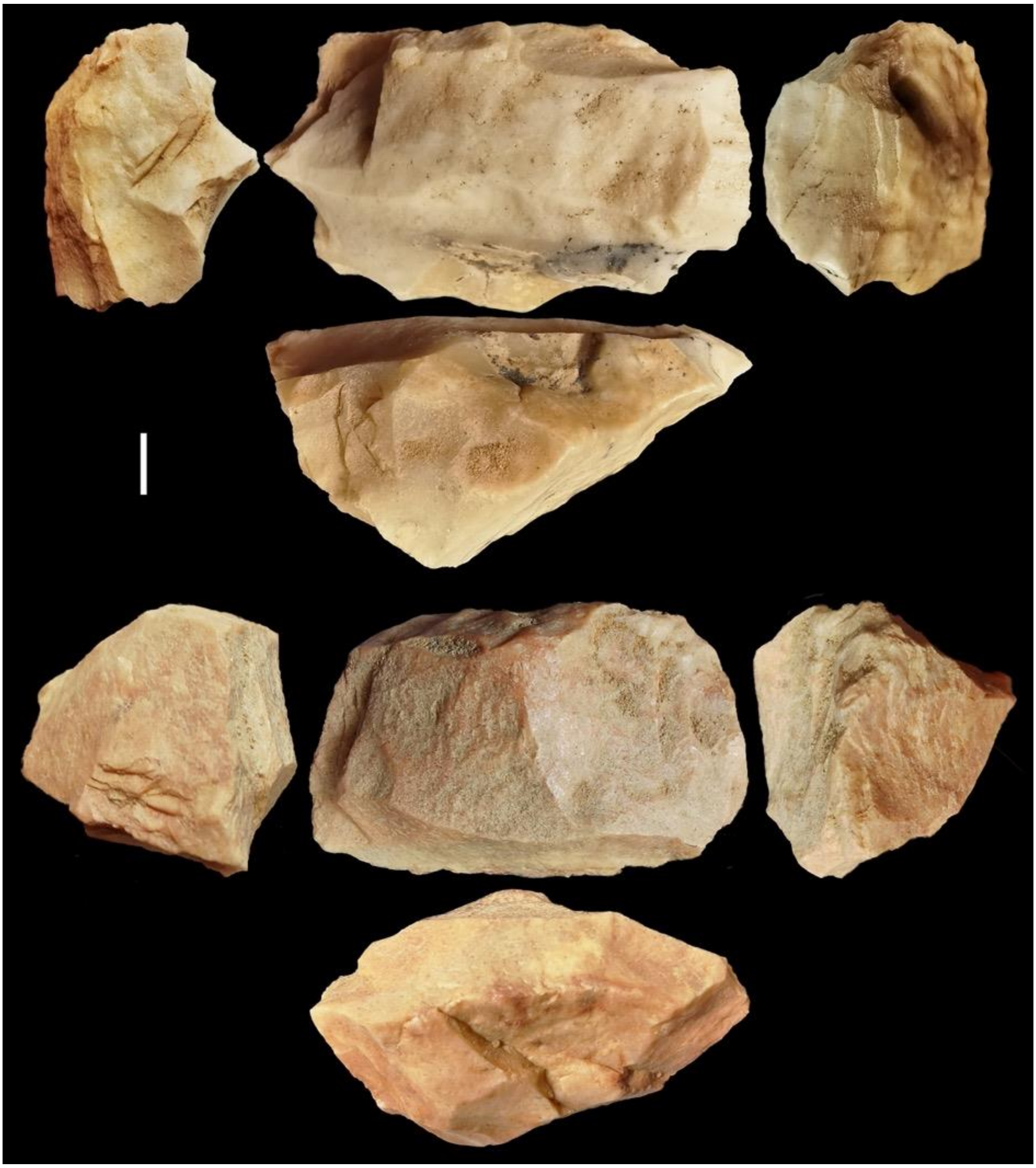

Supplementary Figure 29 Silcrete naviform cores. The upper piece is from the surface at ARN and the lower piece is from JMI7 layer 4. Scale bar is 1 cm long

3.1.3. JMI

Two excavations were conducted at nearby localities at the base of Jebel Misma, JMI7 and JMI8, two boulders with large petroglyph panels (Supplementary Note 2). JMI7 produced 100 lithics while JMI8 produced 519. Milky quartz was the dominant material in both sequences (Supplementary Data 3), eroding as rounded pebbles out of the jebel itself and thus readily available on site. Silcrete was the next most popular material, followed by chert and ferruginous sandstone. A small quantity of the obsidian was found mainly in the JMI8 sequence, while there were very rare occurrences of chalcedony and smoky quartz. Three pink quartzite artefacts were recovered from the

early layers 6 and 7 in JMI8, and there were two examples of the dark chert from layer 4 at JMI7 and layer 3 at JMI8. A notable diachronic change was an increase in silcrete and a relative decrease in quartz going up through both sequences. There was also a relative decrease in ferruginous sandstone and chert in the upper part of both sequences. A chi-square test comparing material distribution between JMI8 layers 1-3 and layers 4-7, lumping obsidian, chalcedony, smoky quartz, and quartzite together, confirmed the significance of variation between the upper and lower sequence ($n=519$, $p<0.001$).

Two chert flakes from JMI8 layers 2 and 5 had ground platforms, linking to the technology at ARN. The only core recovered from the excavations was a silcrete naviform piece from layer 4 in JMI7 (Supplementary Figure 29), indicative of the PPNB in the Levant and further north in Arabia[20]. A surface find of a broken silcrete Helwan point between the two localities (Supplementary Figure 30) confirms a PPNB presence at the site.

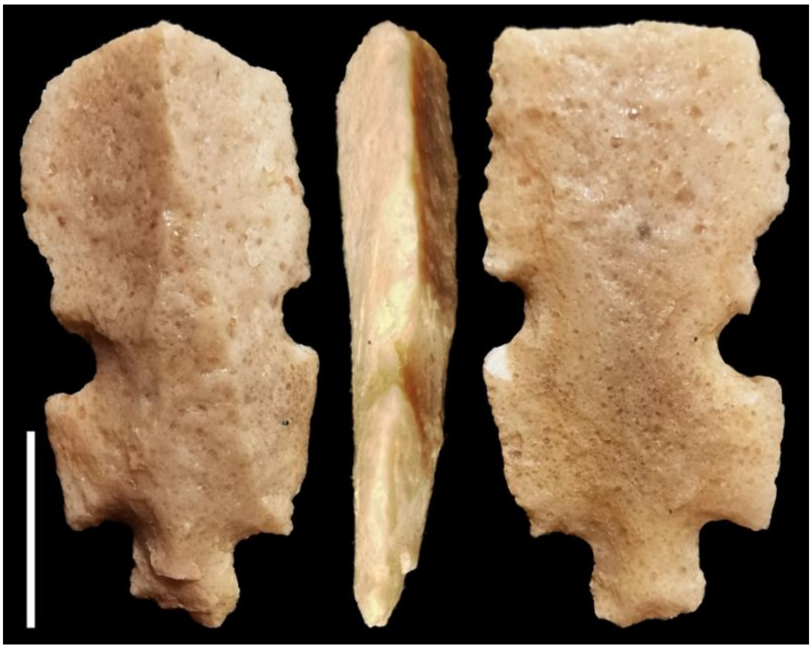

Supplementary Figure 30 Broken silcrete Helwan point from JMI. Scale bar is 1 cm long

The JMI7 and JMI8 excavations produced a total of just 9 retouched artefacts (Supplementary Table 2). These were almost all chert, except for a chalcedony scraper and an end-scraper made on a recycled fine-grained volcanic Middle Palaeolithic flake, both from JMI7 layer 4. In this same layer there was a chert bifacial point with a broken butt and a burin removal along one edge, the latter perhaps from a high velocity impact. The arrowhead is not complete enough to be assigned a type, but it would not be inconsistent with a Byblos point. A key artefact from JMI7 is an opposed notch chert blade in the lowest layer (5) indicating commonality with ARN (Supplementary Figure 31). Two other chert notched blades were recovered from JMI8 layer 4, but these did not have the same distinctive opposing notches. This same layer also produced a chert drill with a distal break. A burin spall from a unifacially marginally retouched edge and a burin were recovered from JMI8 layers 2 and 3 respectively.

The connection between silcrete and PPNB artefact types (Supplementary Figure 29 and Supplementary Figure 30) suggests the transition in materials seen in the JMI

sequences reflects the transition to the PPNB from an earlier culture, most likely PPNA given the connections between the lower levels of the JMI sequences and the upper levels of ARN in the opposed notch blade and the relatively high proportions of milky quartz and ferruginous sandstone.

The only artefacts visible on the surface at these sites that were diagnostic of earlier periods were Levallois cores, an example of which was documented at the southern end of JMI (Supplementary Figure 32). This suggests this landscape was not occupied between the Middle Palaeolithic and the arrival of the population that produced this ancient art.

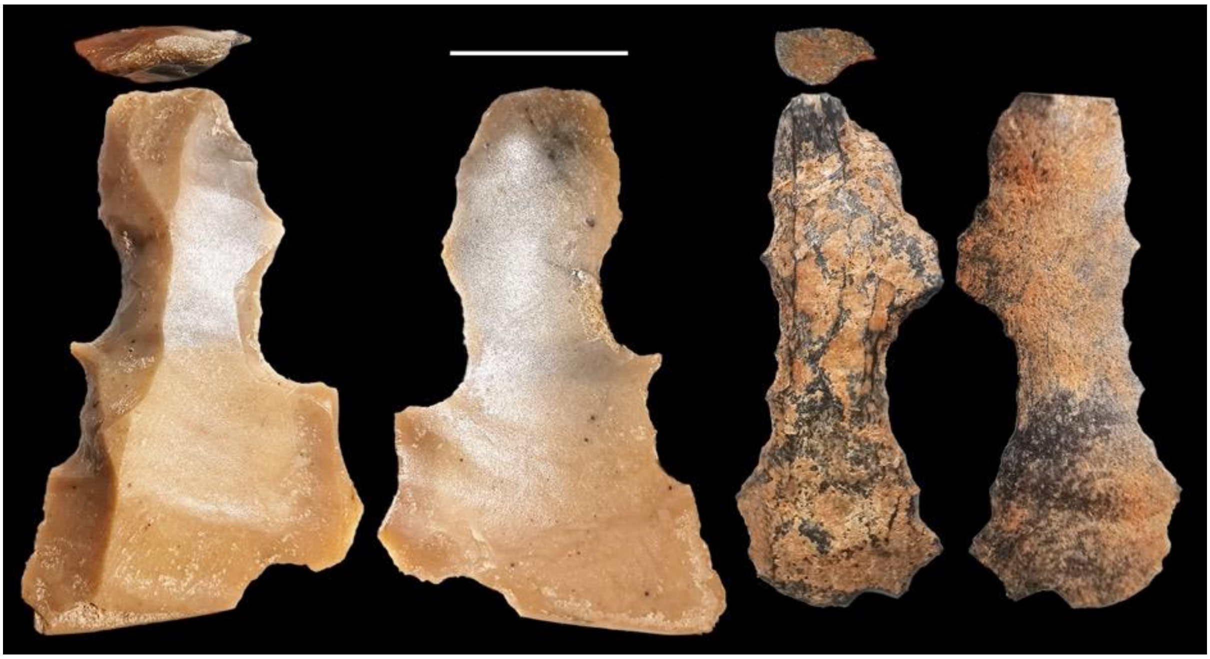

Supplementary Figure 31 Opposed notch chert bladelets from ARN3 T1 disturbance layers (left), and JMI7 T1 layer 5 (right). Scale is 1 cm long.

Supplementary Table 2 Breakdown of Jebel Misma excavated retouched artefacts by locality.

**Retouched types**	**JMI7**	**JMI8**	**Total**
Scraper	2	1	3
Burin/spall	0	1	1
Notched blades	1	2	3
Borer	0	1	1
Bifacial arrowhead	1	0	1
Total	4	5	9

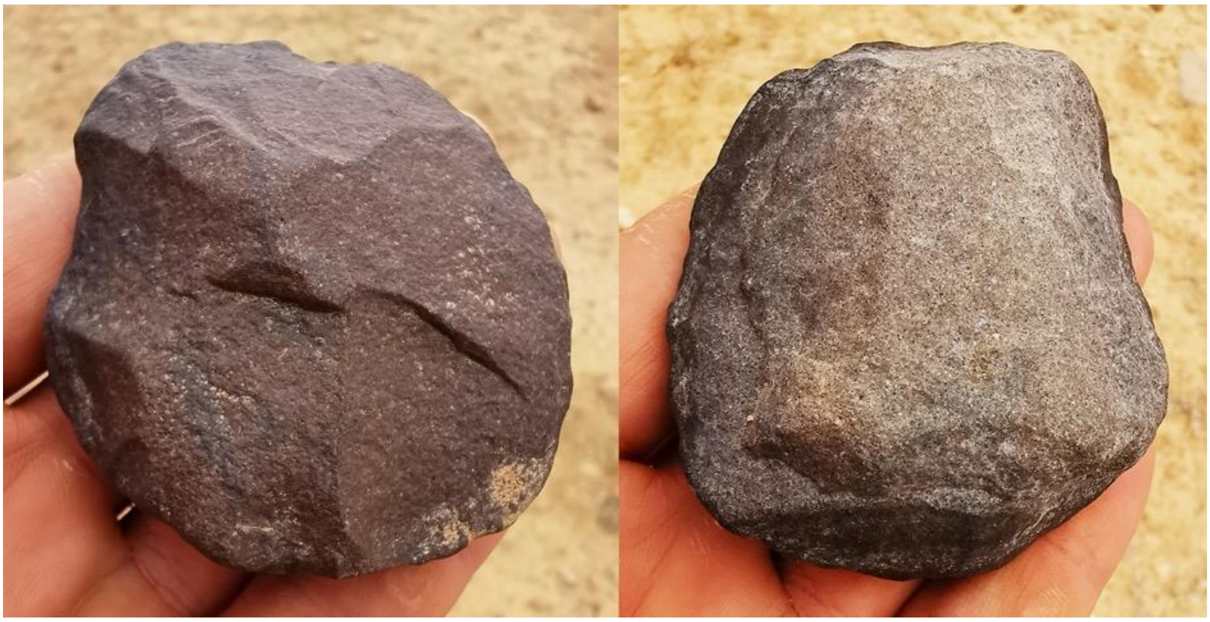

Supplementary Figure 32 Levallois core from the southern end of Jebel Misma.

3.2. Grinding, percussive, and platter stones

3.2.1. ARN grinding stones

Five grinding stones were recovered from the ARN excavations, two from the disturbance layers in T1 and one each from T2 layers 10, 6, and 7. These were all flat grindstones (bottom stones) ranging from 6.34-30.74 mm in thickness, with three made from sandstone and two from ferruginous stone. The specimens from T2 layers 2 and 6 were complete, measuring 180x117x31 and 122x101x14 mm respectively, with the layer 2 piece only very lightly used. The only edge modification on these was some possible unifacial flaking on the sandstone piece from the disturbance layers.

3.2.2. JMI grinding stones

Nine grinding stones were recovered from the JMI excavations, one each from JMI8 layers 4 and 7, and seven from JMI7 layer 4. The four bottom stones were flat, ranging in thickness from 17.83-35.43 mm, with two made from sandstone and two from ferruginous stone. Three of the grindstones had intact edges with two modified through unifacial flaking and one modified through bifacial flaking. A muller (top stone) from JMI8 layer 7 was complete, measuring 78x69x11 mm, with a single flat grinding surface. Two muller fragments from JMI7 layer 4 consisted of a piece of vesicular basalt and a piece of unknown material, perhaps schist, with a convex surface. A sandstone grinding stone measuring 215x168x76 mm from layer 4, had a flat upper ground surface and a concave lower ground surface that could have functioned as a muller for a saddle quern. In relation to this large probable muller, it is worth noting that there were shallow concave grinding slicks on top of the JMI7 boulder (Supplementary Figure 33). The other muller from JMI7 layer 4 was an unusual large complete sub-spherical piece, measuring 103x95x73 mm, with battering indicating its use as a hammer in addition to flat ground facets (Supplementary Figure 34). Such facetted stone balls are a feature of the Levantine Neolithic[18]. On a flat rock behind the two JMI7 and JMI8 boulders there were two deep circular mortar-like cavities pecked into the sandstone, with grinding over the top of the pecking (Supplementary Figure 33). Mortars are typical of the Natufian and PPNA in the Levant (Wright 1994) and have not previously been documented in the Neolithic of northern Arabia (Lucarini et al. 2023).

3.2.3. JMI platter stones

Two flat slabs of stone were found in JMI7 layer 4, with one vertically oriented as though it had been cached. These were a sandstone piece measuring 160x160x30 mm and a mudstone piece measuring 200x180x30 mm with flat but not ground upper surfaces (Supplementary Figure 35). The sandstone piece had been flaked on one edge, while the mudstone piece (a manuport) had a naturally smooth surface. More formally shaped limestone platters have been documented at PPNA sites in the southern Levant[21].

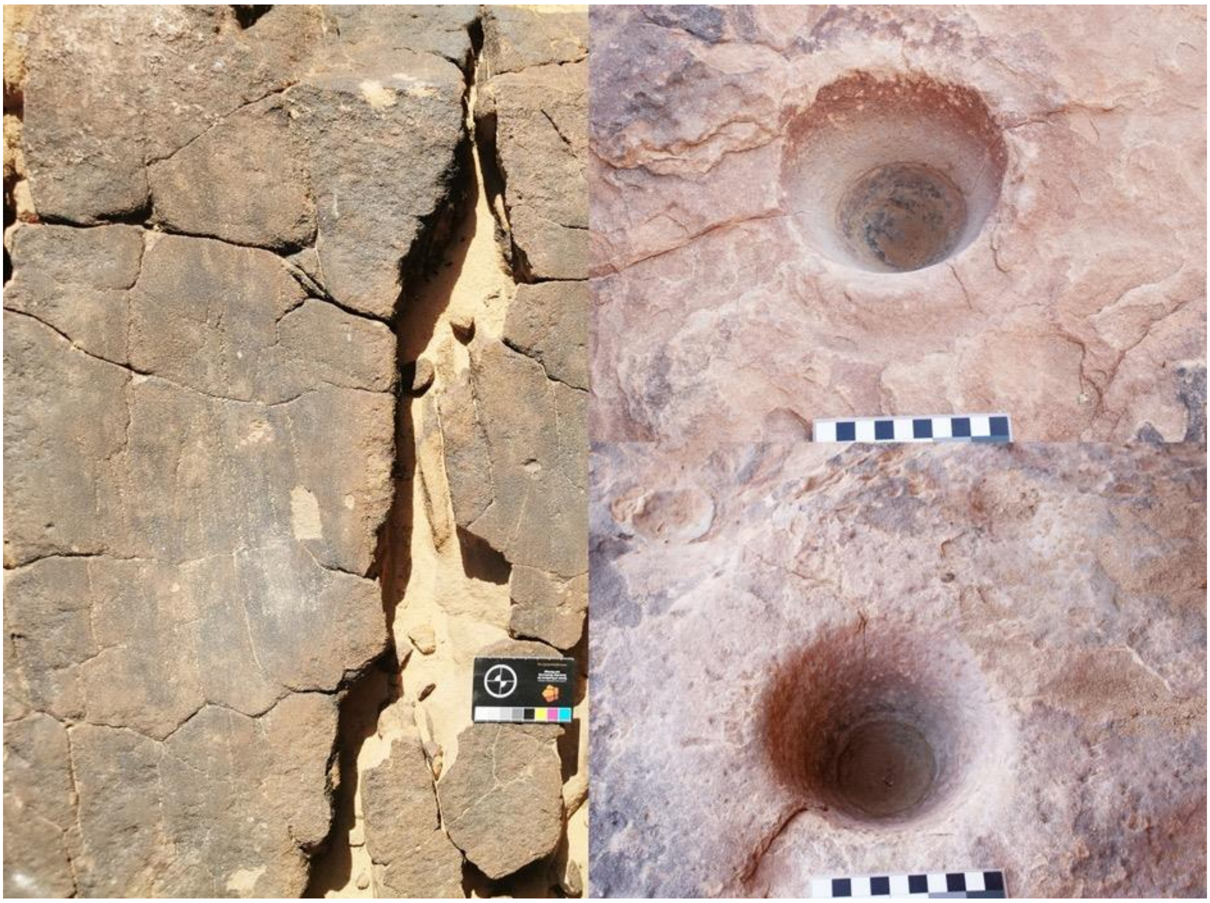

Supplementary Figure 33 In situ grinding features at JMI. Left is a shallow concave grinding slick on top of the JMI7 boulder. Right are two mortars pecked into the bedrock on a flat rock between the JMI7 and JMI8 boulders. Note the deep parallel striations on the grinding slick suggesting use of a heavy muller. Note that the mortars have grinding over the pecking, suggesting they were utilitarian features as opposed to the shallower unground cupules present at ARN. Scale on the left is 8 cm long, scales on the right are 10 cm long.

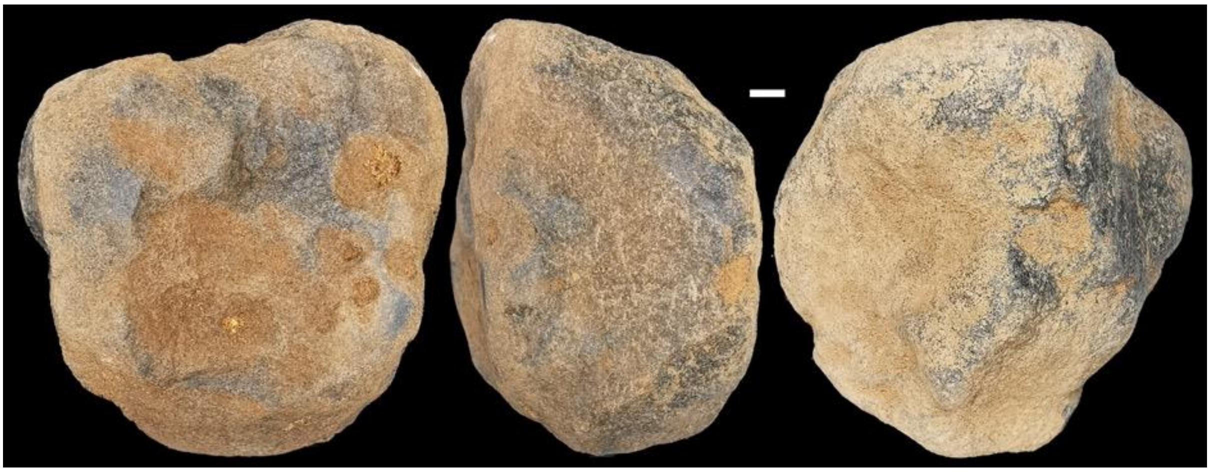

Supplementary Figure 34 Grinding stone from JMI7 layer 4 with both ground facets and battering on some edges. Scale bar is 1 cm long.

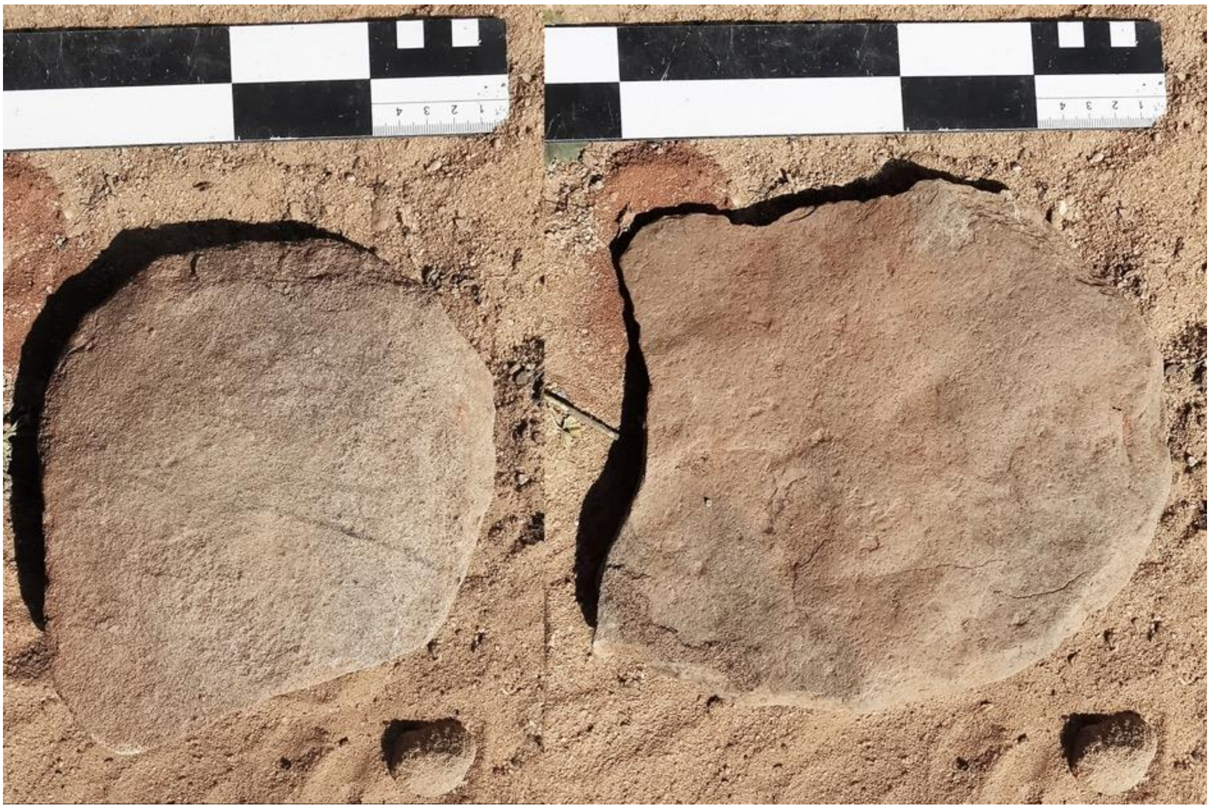

Supplementary Figure 35 Platters from JMI7 layer 4. The piece on the right is a sandstone slab with a few flake scars on the upper edge. The piece on the left is a mudstone slab with a naturally smooth surface.

3.3. Pecking and smoothing stones

On the surface at JMI8 we found a large wedge-shaped piece of silcrete, 90x58x52 mm, about the size of the internal dimensions of a cupped hand, with percussion marks on either end (Supplementary Figure 36). This is the only piece of silcrete from either the excavation or the surface of the site that was not flaked and it is also the largest; we think it was used in creating the petroglyphs as it has extensive battering on both narrow ends of the piece. Silcrete tools were used to create the petroglyphs at the Camel Site on the north side of the Nefud desert[22].

On the surface at ARN30 we recovered an artefact that may have been used to peck the rock art. This was a wedge-shaped flake of ferruginous sandstone, a particularly tough material, not locally available at the ARN rock art localities. It measured 114x95x47 mm, again about the size of a cupped hand. The flake has three flake scars on its distal end, perhaps to create a more robust tip, with battering both over the top of these removals and on the pointed protrusion on the proximal part of the flake (Supplementary Figure 36).

A further example of a wedge-shaped pecking stone was found in the excavation at ARN3 T1. This piece is also wedge-shaped, the size of a cupped hand, and made of ferruginous sandstone with unifacial scars on its narrow edge and battering over the top of these (Figure 5), showing the battering occurred after the flakes were removed, similar to the example from ARN30. Like the ARN30 and JMI8 examples it also has battering on a protrusion at the opposite end of the tool (Supplementary Figure 37), indicating it was used extensively. The topography fits very comfortably in the right hand in a four-jaw chuck grip, either way up; with grooves created by flake scars for the index finger and separate surfaces for the thumb, middle finger, and ring finger, with the former opposing the latter two (Supplementary Figure 38). In this grip, the narrow semi-circular end of the tool protrudes to allow for repetitive hammering without risk to the hand.

The wedge-shaped form of these pecking stones indicates the battering on them was not from freehand percussion. Free-hand knapping requires spherical percussive surfaces: The arced hand movement when following through after striking the core requires the force to dissipate across the hammer surface rather than exit and chip the hammer. In contrast petroglyph creation involves straight-on percussive movements so may be more easily and accurately achieved with a protrusion on an angular stone as in the examples in Supplementary Figure 36. If such wedge-shaped pieces were used in free-hand percussion the pointed protrusions would break off.

Some of the petroglyphs seem to have undergone a second stage of line creation whereby pecked marks were joined and smoothed through linear rubbing (Fig. 4). It is possible that the silcrete piece from JMI8 was also used in this way with its broader rounded contact edges in comparison to the other pecking stones. Another stone that appears to have mainly used in this way was recovered from the surface at ARN44 (Supplementary Figure 39). This is a large ferruginous flake with a worn ridge that protrudes when the stone is held in a four-jaw chuck grip that would allow the stone to

be moved back and forth in line with the ridge. Unifacial flaking on one end of the tool allows the index finger to reach over in line with the ridge (Supplementary Figure 39) and thereby guide this type of motion.

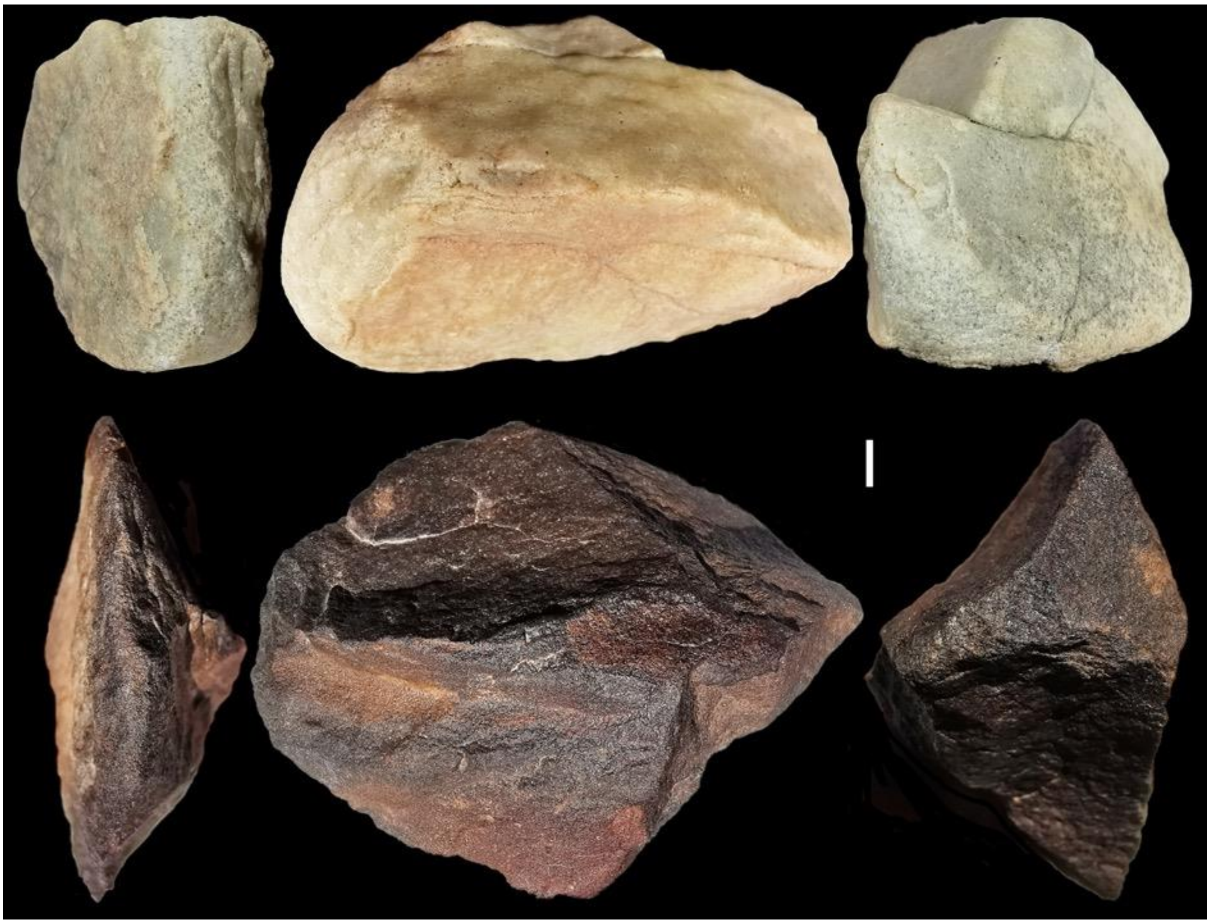

Supplementary Figure 36 Pecking stones from ARN and JMI. Above, unflaked silcrete clast from JMI8 with extensive battering on both narrow ends. Below, ferruginous sandstone large flake from ARN30 with battering on both pointed ends (distal left, proximal right). Scale is 1 cm long.

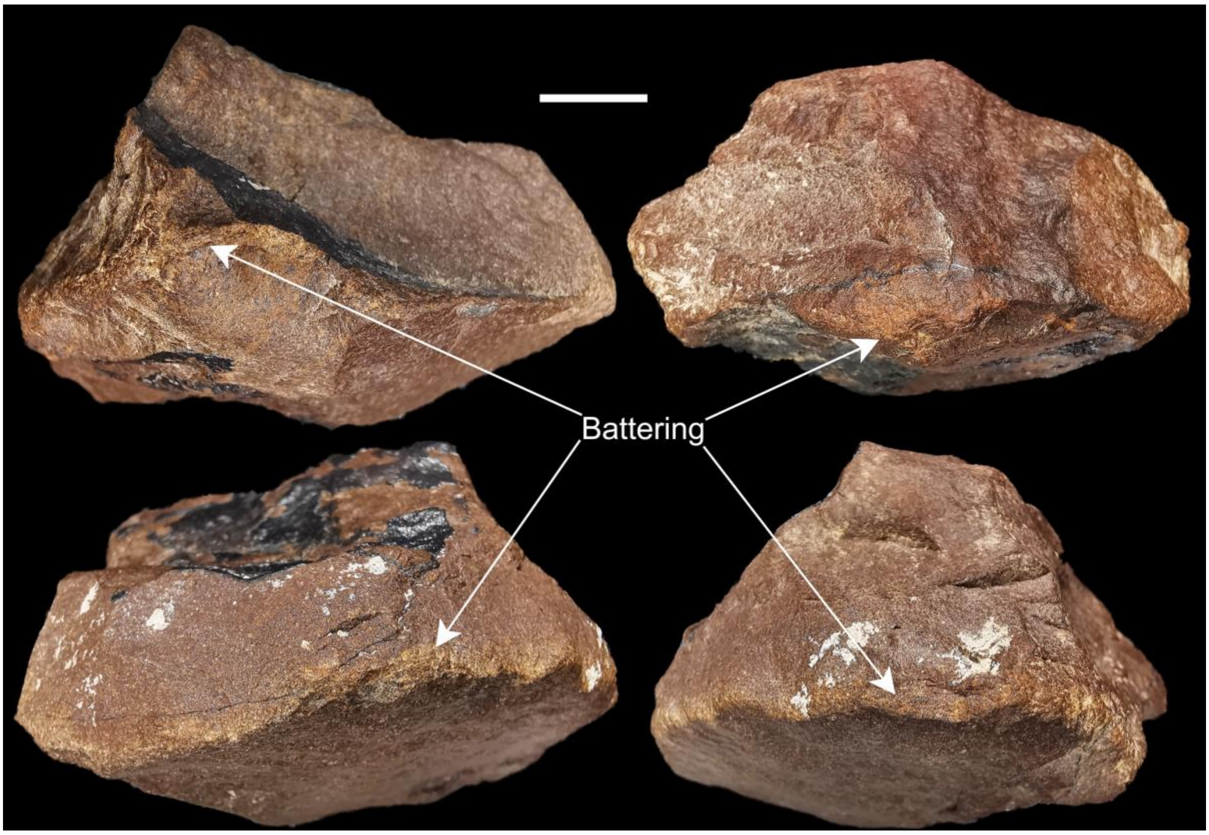

Supplementary Figure 37 Excavated pecking stone from ARN3 showing extensive battering.

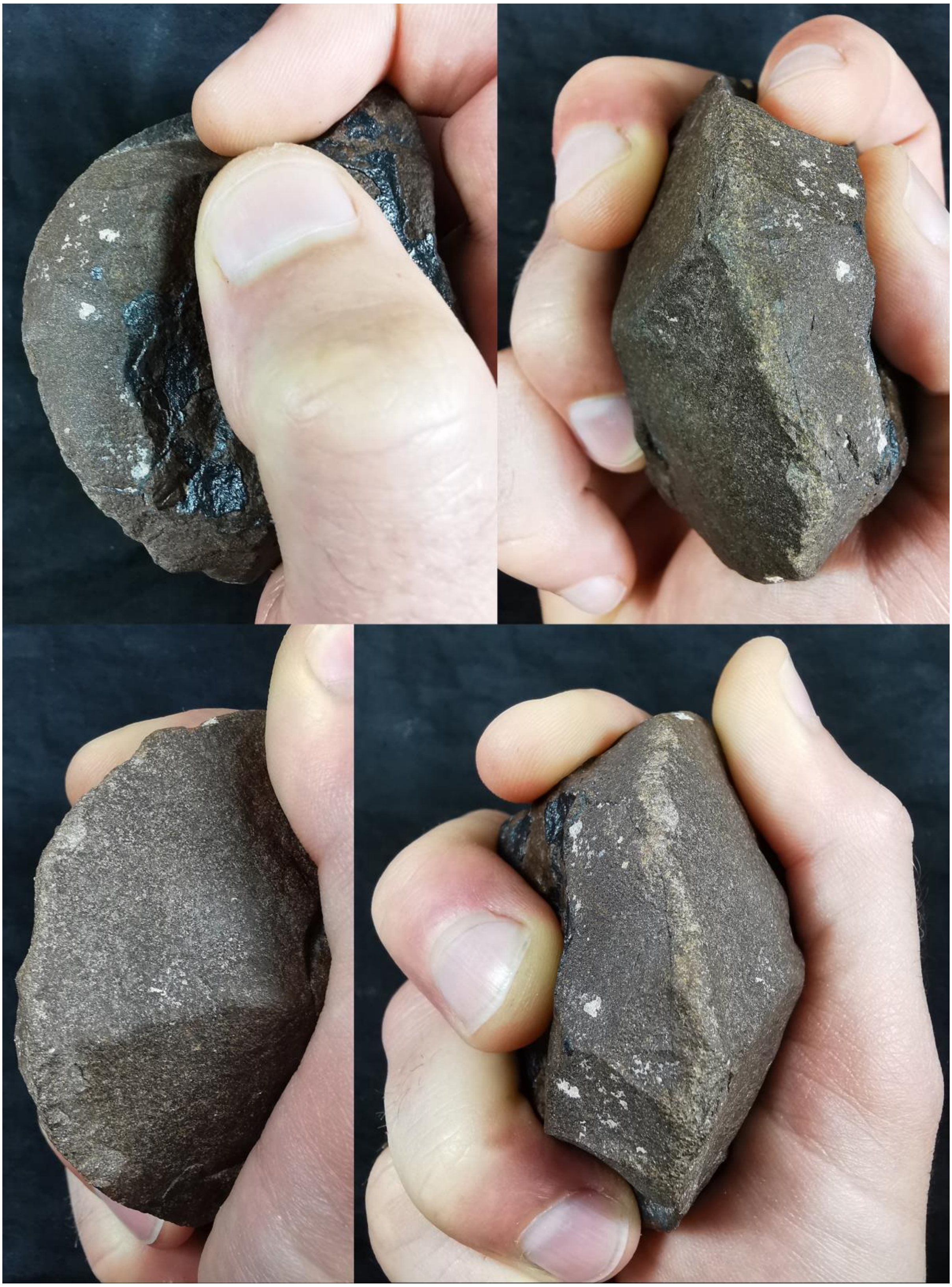

Supplementary Figure 38 Excavated pecking stone from ARN3 in a four-jaw chuck grip in both orientations. Note the battering on the semi-circular protruding edge.

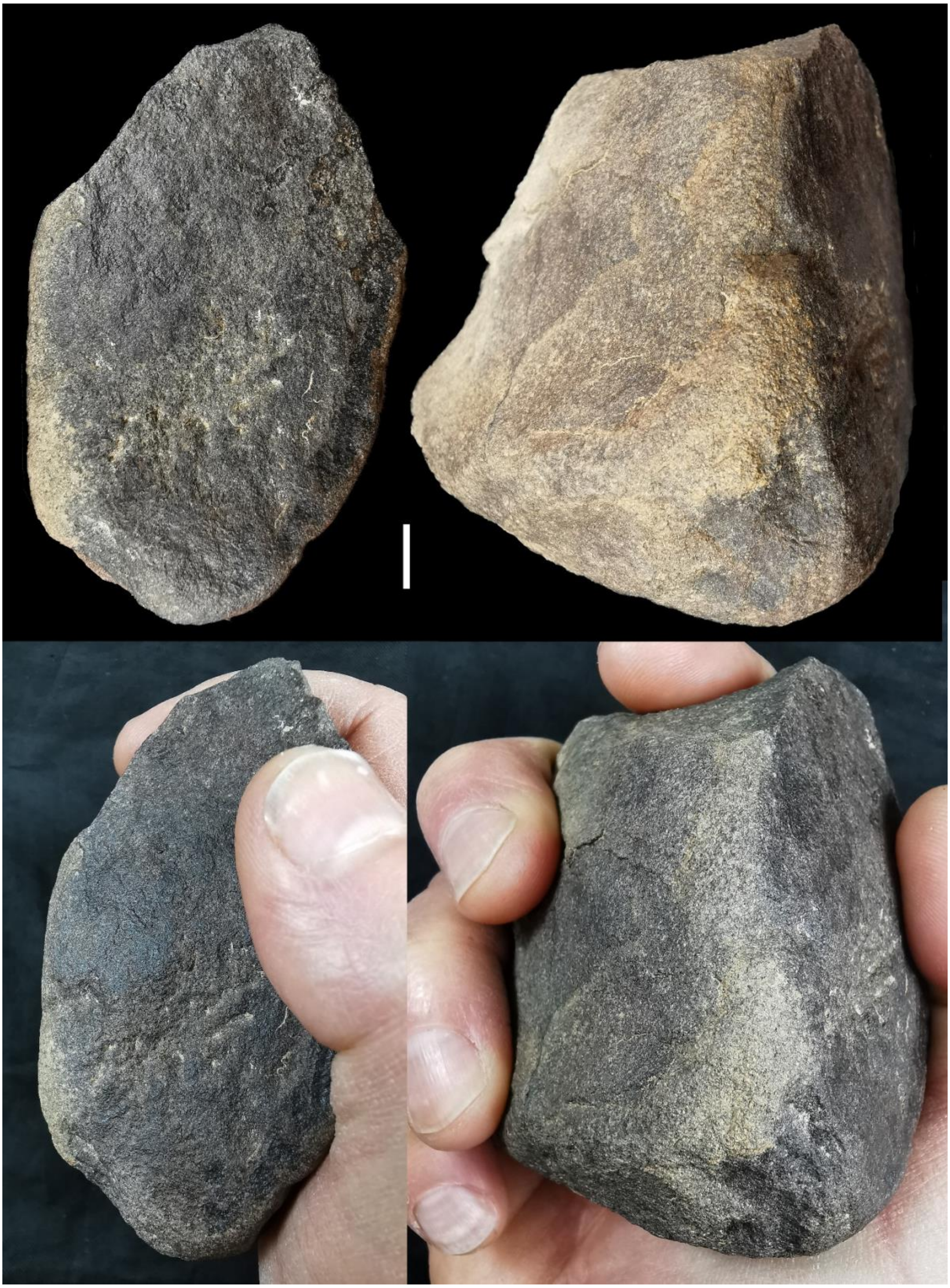

Supplementary Figure 39 Possible ferruginous smoothing stone from ARN44. Note the wear on the ridge which protrudes when held in a four-jaw chuck grip that would allow the stone to be moved back and forth in line with the ridge. Note the left view also shows percussion damage in the centre of the face, where the stone was used an anvil before this large flake was taken off.

3.4. Decorative artefacts

3.4.1. Pigment

Pigment at both ARN and JMI was often in the form of red ferruginous shale which occurs more broadly across northwest Arabia running in seams at the base of sandstone jebels[23]. Two examples of this with striations on were documented in the disturbance layers at ARN3 T1, weighing 2.5 g together. In total 87.6 g of red pigment was recovered from ARN3 T1 and T2 with the bulk of this (84 g) coming in a single slab in T2 layer 7. There were also examples of green and white pigment from ARN3 T2. A total of 4.6 g of green copper ore pigment was excavated, largely in the form of a crayon from layer 7 weighing 4.4 g (Supplementary Figure 40). White chalky pigment totalled 8.7 g and included two crayons from layer 7 and one from layer 8 (Supplementary Figure 40). In addition to the coloured pigment 17.7 g of mica was recovered from ARN3, a material which produces a sparkly effect when crumbled. Most of this came from T2 layers 7 (9.8 g) and 8 (4.8 g). A manuport of a triangular stone with different coloured stripes was found in T1 layer 2.

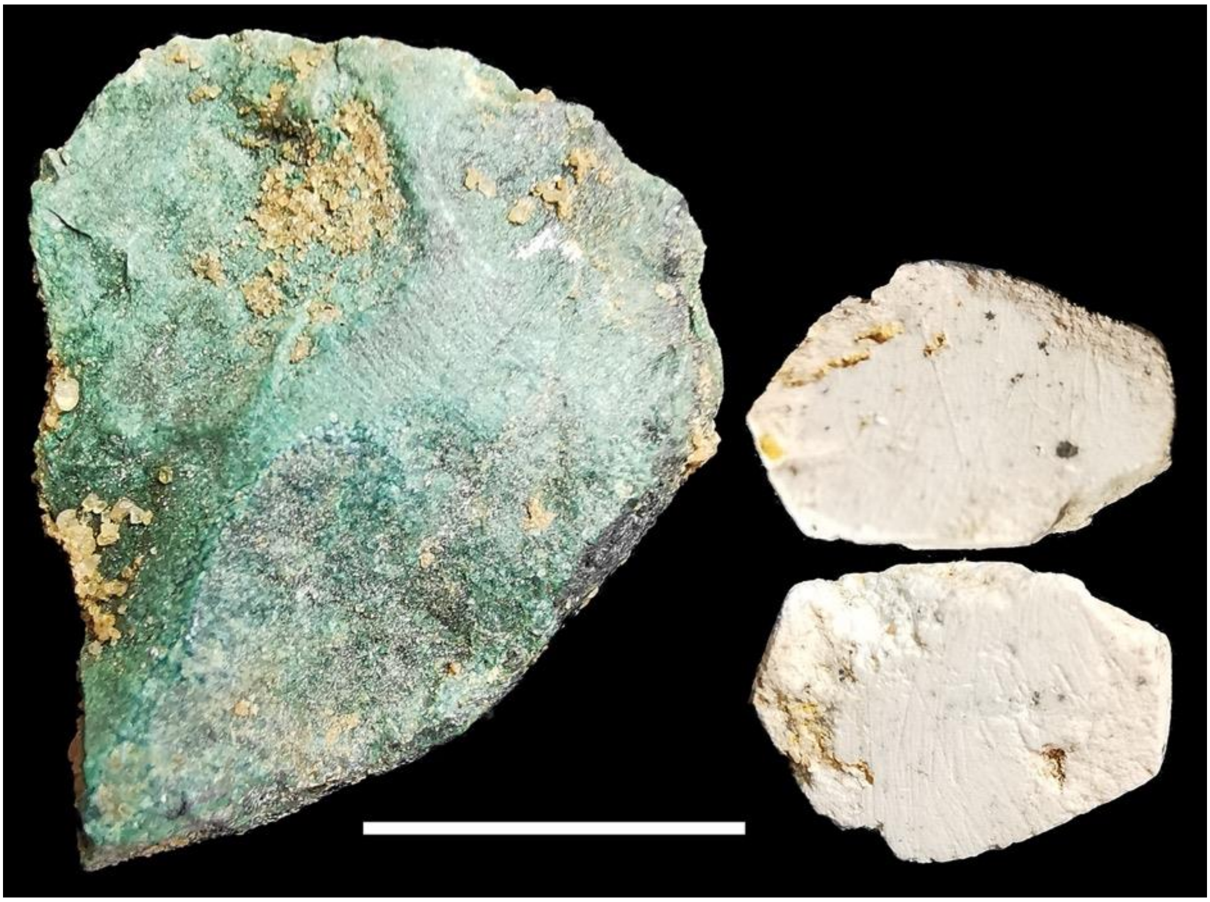

Supplementary Figure 40 Pigment crayons from ARN3 T2 layer 7. Left is green copper ore pigment with a striated facet in the centre, right is a white chalky pigment with two flat striated surfaces. Scale is 1 cm long.

JMI7 produced 27.4 g of red pigment, largely (25.6 g) from layer 3. A big piece (140.3 g) of cream coloured sparkly pigment with some striations was found in layer 4, while a single lump of haematite (197 g) was found in layer 5.

3.4.2. Beads

Six beads were found at ARN3 T2. Two ground stone disc beads were recovered from layer 9, one of which was broken, as well as two such beads from layer 7, again one of which was broken (Figure 4; Supplementary Figure 41). The beads were made on a dark green siliceous stone with pale speckles. Along with the use of copper ore pigment this is perhaps part of a broader preference for greenish hues in the PPNA[24]. Both the broken beads had roughly facetted perimeters (Supplementary Figure 41), unlike the circular complete ones, and they both had larger diameters than the complete ones (9.34 and 8.31 mm versus 4.6 and 5.72 mm). This suggests breakage on site during manufacture and that edge grinding was the final stage of production. On-site manufacture accords with the presence of the lithic drills (Supplementary Figure 24). The width of the complete drill 1.5 mm above the tip is 1.52 mm, narrower than the maximum aperture width of all four disc beads (2.24, 2.1, 1.73, and 2.09 mm). The bead holes were drilled from both sides with the example in Supplementary Figure 41 showing mis-alignment of the holes. Stone disc beads are common in the Natufian and PPN of the Levant[25]. The diameter of the two complete beads (4.36 mm and 5.72 mm) fall within the typical range (3-6 mm) for the large assemblage of PPNA ground disc beads at Shubayqa 6 on the Harrat as-Sham in Jordan[26]. A further bead from ARN3 T2 was a small unground stone piece from layer 5, made on a greyish brown stone, measuring 6.01 mm in diameter, 2.07 mm thick, and with an aperture 1.55 mm wide (Supplementary Figure 41).

Two beads from ARN3 T2 were made of smooth shelled Dentalium (tooth-shell), one from layer 7 and one from layer 8 (Figure 5). Dentalium beads were used in the Levant from Upper Palaeolithic to Chalcolithic times, but are most frequent in the Natufian and PPN[27]. Dentalium beads vary in length from 1-60 mm, with late and final Natufian beads being particularly short (modal length 1-5 mm), while those of the early Natufian and PPNA are moderately sized (modal length 6-10 mm)[28]. Dentalium beads from the PPNA occupation of Salibiya IX in the Levant range from 2-15 mm with an average of 7 mm[25]. The beads from ARN3 measure 11.41 and 7.64 mm long, consistent with a PPNA attribution. Dentalium are marine molluscs with a global distribution including the Red Sea, meaning the shells came from at least 320 km away.

A single ground stone disc bead was recovered from JMI8 layer 2 (Supplementary Figure 41). This was made on a granular greyish blue stone, with rough unground edges suggesting it was unfinished and that there was on-site manufacture, as also indicated by the broken lithic drill from JMI8 layer 3. The bead measures 8.54 mm diameter, 1.51 mm thick, with an aperture width of 2.07 mm, and like the beads from ARN3 appears to have been drilled from both sides (Supplementary Figure 41).

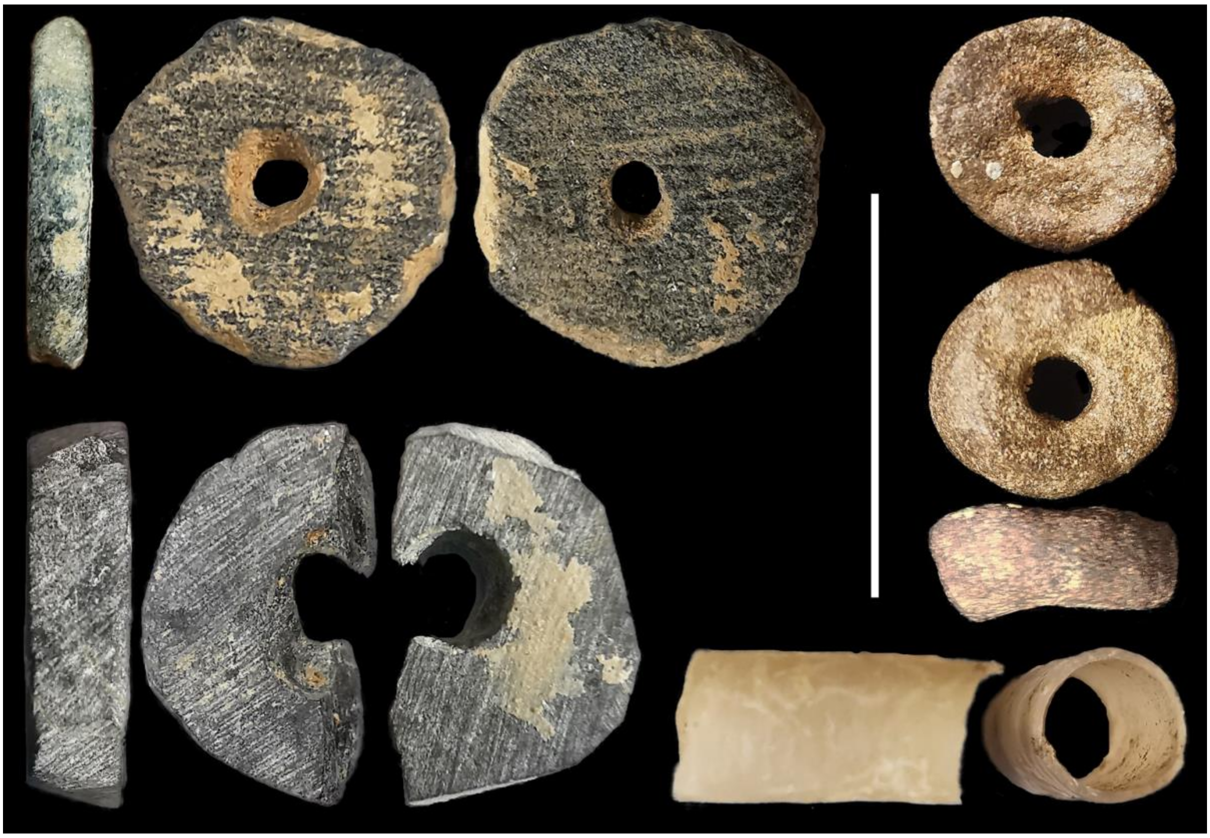

Supplementary Figure 41 A selection of beads from ARN and JMI. Top left is a ground disc bead from JMI8 layer 2; top right is an unground stone bead from ARN3 T2 layer 5; bottom left is a broken ground disc bead from ARN3 T2 layer 9; bottom right is a Dentalium bead from ARN3 T2 layer 8. Note that both disc beads appear to have had their holes drilled from both sides with that from ARN3 having an unfinished mis-aligned hole on the left surface. Scale is 1 cm long.

Supplementary Note 4: ARN and JMI Faunal remains

Only three very small fossil assemblages were recovered from excavations at JMI, MLH, and ARN. The former comprises 13 small bone fragments, including a small rib and three enamel fragments, as well as a single ostrich (Struthio camelus) eggshell and a pearl oyster shell fragment. At MLH only a single small enamel fragment was found, likely from an ungulate, while at ARN two bone fragments were found, including a small microfauna midshaft. All the material is poorly preserved, and there is no evidence of burning.

Supplementary Note 5: Geology, geomorphology and paleoenvironment

The geology of the study area is characterized by Early Ordovician to Early Silurian sandstones of the Tabuk and Tayma groups, forming the mountains of Jebel Arnaan in the west, Jebel Mleiha in the centre, Jebel al-Misma in the east, as well as the lower sandstone formations of Sahout in the north[29]. The sandstones were formed in fluvial to transitional marine environments[29], and are mainly composed of well cemented quartz with a minor clay component[30], forming the ideal substrate for rock engraving. These massifs are surrounded by Quaternary sand dunes of the Nafud desert. Thirty playa deposits of different sizes exist in the region, fed by a network of ephemeral rivers (Supplementary Figure 42) and most commonly found at the base of jebels. Six of the paleolakes were investigated for their sedimentology. At four locations in the vicinity of the archaeological sites (Supplementary Figure 42) trenches were dug for stratigraphic and sedimentological analyses. Dating of sandy layers within the sediments was conducted using luminescence dating; and characterization of the bulk mineralogy was conducted using XRD.

Playas at the foot of alluvial fans were investigated west of Jebel Arnaan (Supplementary Figure 42, Sites 1 & 2) and Sahout (Supplementary Figure 42, Site 3). The playa at Site 1 has a surface area of 0.25 km^2, with a trench in the centre revealing a 1.95 m thick, fine-grained sediment succession, before encountering gravels (Supplementary Figure 43A). Site 2 was a 0.4 km^2 playa, but only 45 cm deep when excavated; like Site 1 it was underlain by coarse gravels. Site 3 was just 0.03 km^2 with 38 cm of deposit sitting on Paleozoic sandstone bedrock. The latter two deposits were too shallow to be dated.

Several playa lake sites are located west of Jebel Misma (Supplementary Figure 42). We chose to investigate the largest one (Site 4), which sits against a steep exposure of Palaeozoic sandstones, covers an area of approximately 0.23 km^2, and is fed from the north by an ephemeral stream running along the jebel base that feeds into several playas along the way (Supplementary Figure 43B). A trench in the middle of the playa revealed 1.75 m of fine-grained sediment underlain by coarse-grained gravels.

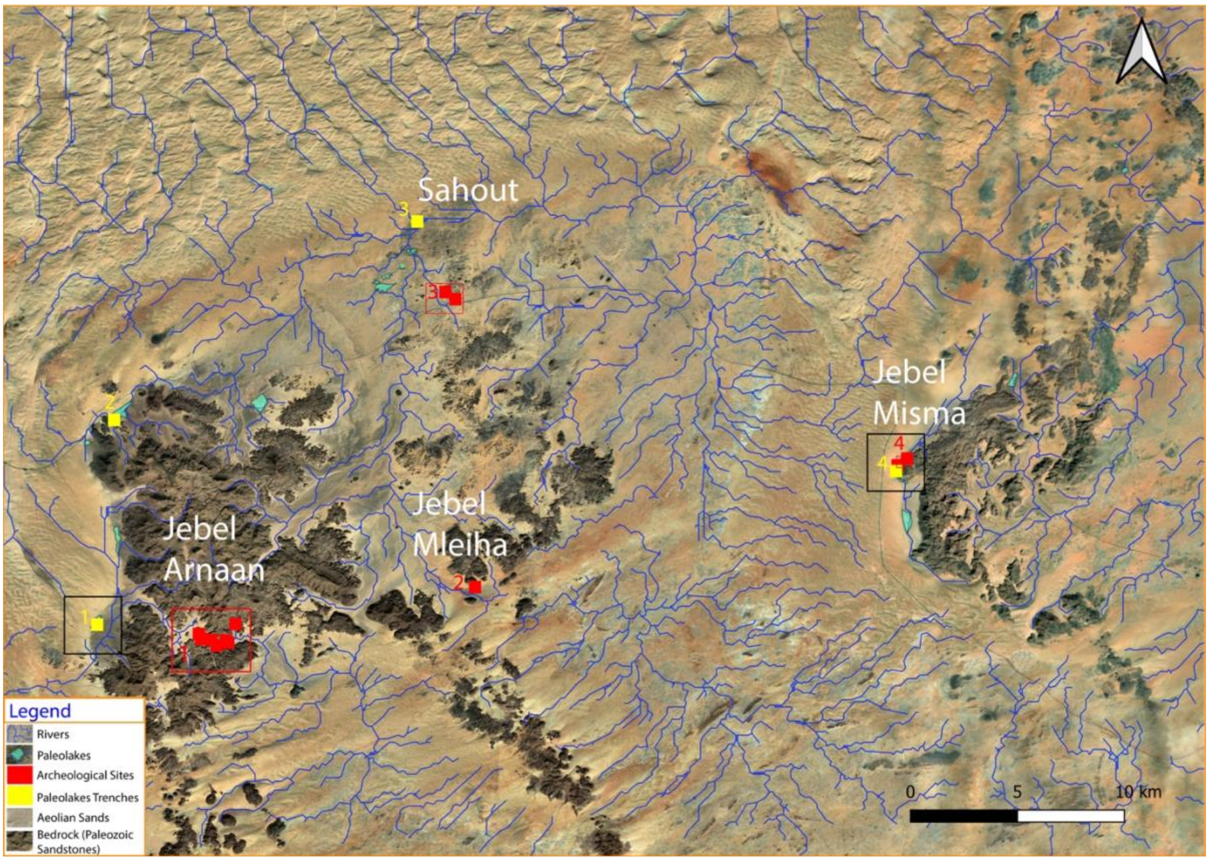

Supplementary Figure 42 Location of excavated playa sites and rock art panels in the study area. Satellite image overlain with playas and rivers. Yellow squares: excavations of playa sediments (site numbers are referenced in the text). Red squares: archaeological sites. Black squares: locations of the site maps shown in Supplementary Figure 43. Water courses were mapped following Breeze et al. (2016); 31 playa lakes were delineated manually using QGIS with a Esri World Imagery base. Sources: Esri, DigitalGlobe, GeoEye, i-cubed, USDA FSA, USGS, AEX, Getmapping, Aerogrid, IGN, IGP, swisstopo, and the GIS User Community

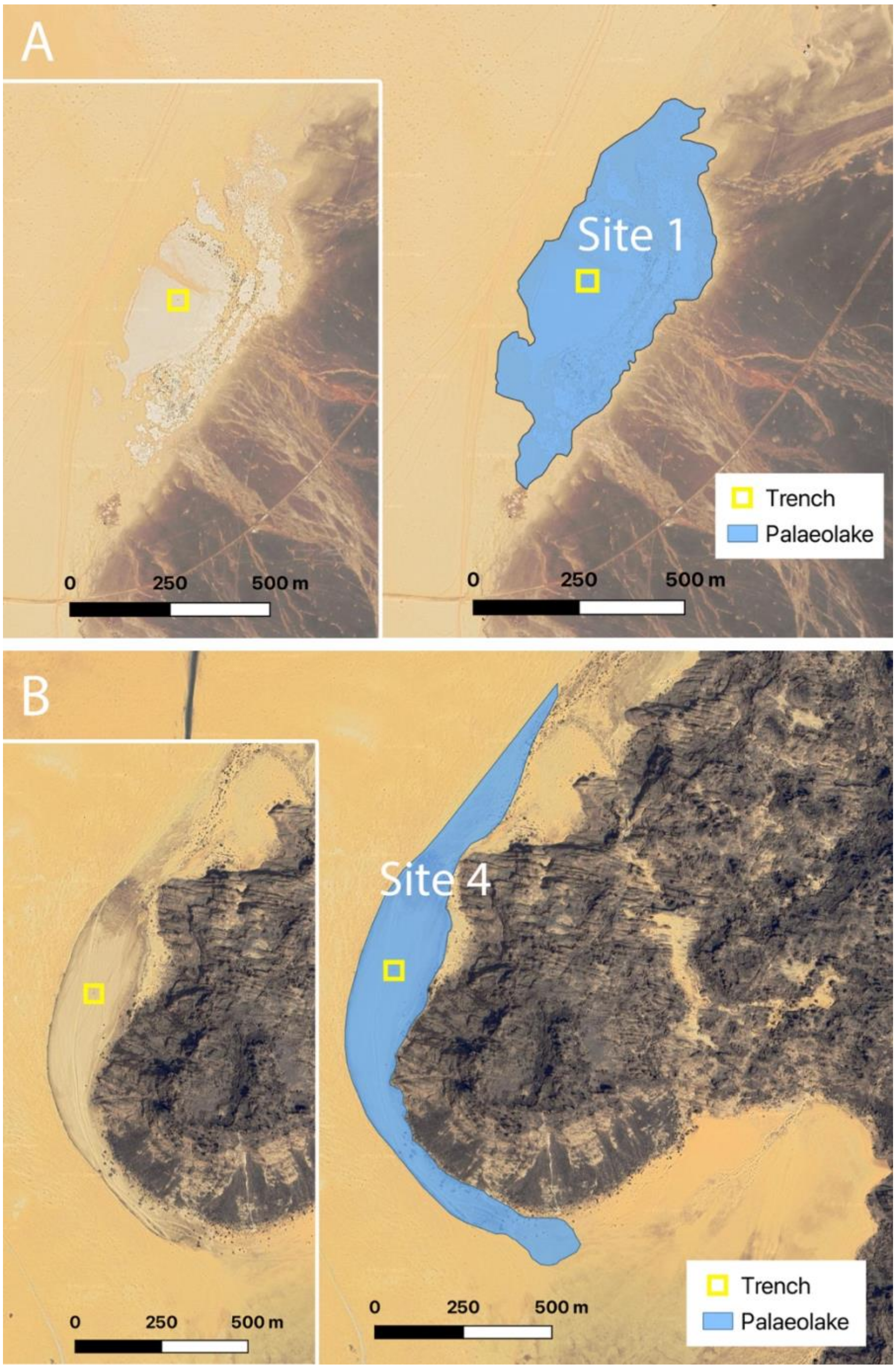

Supplementary Figure 43 Maps and satellite images of sites 1 and 4. ***A:*** *Map of the paleolake west of Jebel Arnaan. Inset: satellite image of the mapped palaeolake.* ***B:*** *Map of the paleolake at Jebel Misma. Inset: satellite image of the mapped palaeolake. Excavated trenches are shown in yellow. Map data: © Google, Maxar Technologies, produced using Google Maps satellite imagery (Basemap layer in QGIS), retrieved 29 July 2025.*

5.1. Sedimentological observations and dating

Grain size and sorting was observed visually and textually in the field and classified according to standard the sedimentological classification. The sedimentological and lithological characteristics of the two thickest sections are similar. At Site 1 (Supplementary Figure 44A) the basal part consists of poorly sorted gravel with 0.5-5 cm size pebbles derived from the nearby outcrops of Paleozoic sandstones. This is overlain by 5 cm of clays with mud cracks at the top. Above this is a 20 cm thick layer of unconsolidated, reddish fine-grained sands, again with mud cracks at the top. The rest of the succession (170 cm) consists of homogeneous, sandy silty clay, which again exhibits mud cracks at the top. At Site 4 (Supplementary Figure 44B) the base also consists of poorly sorted gravel, with similar sized pebbles to Site 1 that originate from eroded sandstone. This is followed by an alternation of clays, silts, and sand consisting of 5 cm of sandy silty clay, 5cm of reddish fine sand, a further 20 cm of sandy silty clay with vugs, then 10 cm of unconsolidated, fine sand and finally 15 cm sandy vuggy clay with small pebbles (<1 cm). The rest of the succession (1.20 m) consists of homogeneous sandy, silty clay and is capped by mudcracks and vugs. The sections at Sites 2 and 3 (Supplementary Figure 46) are much shorter (45 and 38 cm), and sandier (clayey sands). Site 2 overlays poorly sorted gravel at the base and has mudcracks at the top. Site 3 directly overlays Palaeozoic sandstone basement.

Five luminescence ages were obtained from the gravels and sandy layers encountered in the sections, three from Site 1 and two from Site 4. The gravel deposits at the base of Site 1 (Supplementary Figure 44A) produced an age of 68.8±5.0 ka, while 20 and 40 cm higher, the sandy layers interfingering with the playa sediments yielded ages of 15.5±1.5 and 17.1±1.7 ka respectively. These ages are inverted but within the error of each other. We interpret the sediments to be about 16 ka years old. At Site 4 (Supplementary Figure 44B) the gravel deposits were too coarse to sample, but the sands just below the fine-grained playa deposits provided an age of 12.7±2.1 ka. A second luminescence sample, taken 60 cm below the surface, yields an age of 14.2±1.9 ka. It is possible that this latter age is overestimated due to insufficient sunlight exposure of the material prior to deposition, since: a) fine-grained sediments tend to be deposited from relatively turbid water, and b) the slower-bleaching polymineral fine-grained signal ($pIRIR_{225}$)[31] from this sample yields an age of 33.9±2.8 ka. Consequently, we regard the 12.7±2.1 ka age towards the base of the sequence at Site 1 as more reliable.

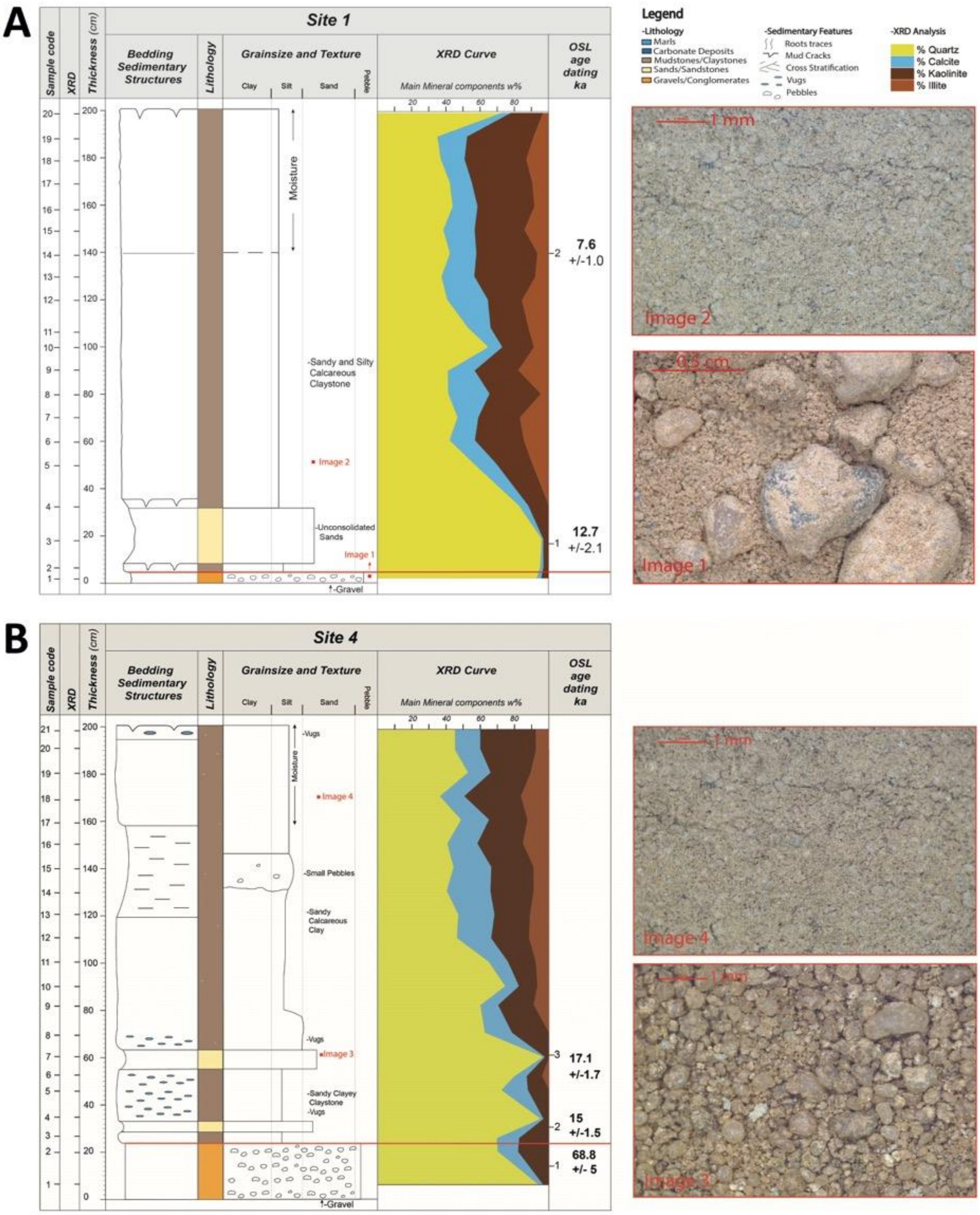

*Supplementary Figure 44 Normalized staked area graphs and sedimentary sections of **A:** Site 1 (ARN) and **B:** 4 (JMI) with XRD analysis of the main mineral components and pictures of the sediment types.*

5.2. Mineralogy

At Site 1, 21 samples were measured for mineralogical content using XRD (Supplementary Figure 45). There is an overall dominance of quartz throughout the section, as also reported by Powers, et al. [30], with the highest values in the gravel and sandy layers, and an overall decrease from about 95% at the base, to about 45% at the top. The clay fraction is represented by kaolinite and illite. Kaolinite is present throughout, with the lowest concentrations in the gravels at the base (around 4%) increasing substantially in the overlying playa sediments to about 33% at the top. The kaolinite may originate from (i) the weathered sandstone bedrock, that consists of quartz (96.5 %) and kaolinite (3.5 %) (the base of Site 3, Supplementary Figure 46), (ii) as an authigenic alteration product, or (iii) through dust deposition[32]. Illite is nonexistent in the gravels and the base of the playa sediments and then starts to increase to 15 % at 60 cm with a subsequent decrease at the top to about 7%. Illite forms in alkaline conditions, by direct precipitation from lake waters or as an alteration product within the sediments[33,34]. We suspect that in this case the illite is authigenic in origin.

Calcite generally follows the kaolinite concentration, starting with 4% at the base of the playa sediments, increasing to about 25% at 130 cm, and then decreasing toward the top of the section to about 15%. Calcite indicates the presence of water, being precipitated either through organic precipitation, or from evaporation of underground and meteoric/surface water in the sediments[35].

At Site 4, 20 samples were analysed by XRD (Supplementary Figure 45B). Again, there is an overall predominance of quartz, with highest values at the bottom of the section where concentrations fluctuate within the gravels and playa sediments between 95% and 60% before falling to about 45% with a final increase to 72% at the top. Kaolinite concentrations start low (4.5%) and fluctuate in opposition with the quartz, then increase in the upper part of the section to 38% before decreasing in the top 10 cm. As was the case in Site 1, illite is not present at the base of the section, it first occurs briefly in small amounts at 50 cm and then continuously from 70 cm when it increases to 18% and then decreases towards the top. Calcite is present from about 35 cm from the base and fluctuates in tandem with kaolinite throughout the section, varying between 3 and 15%.

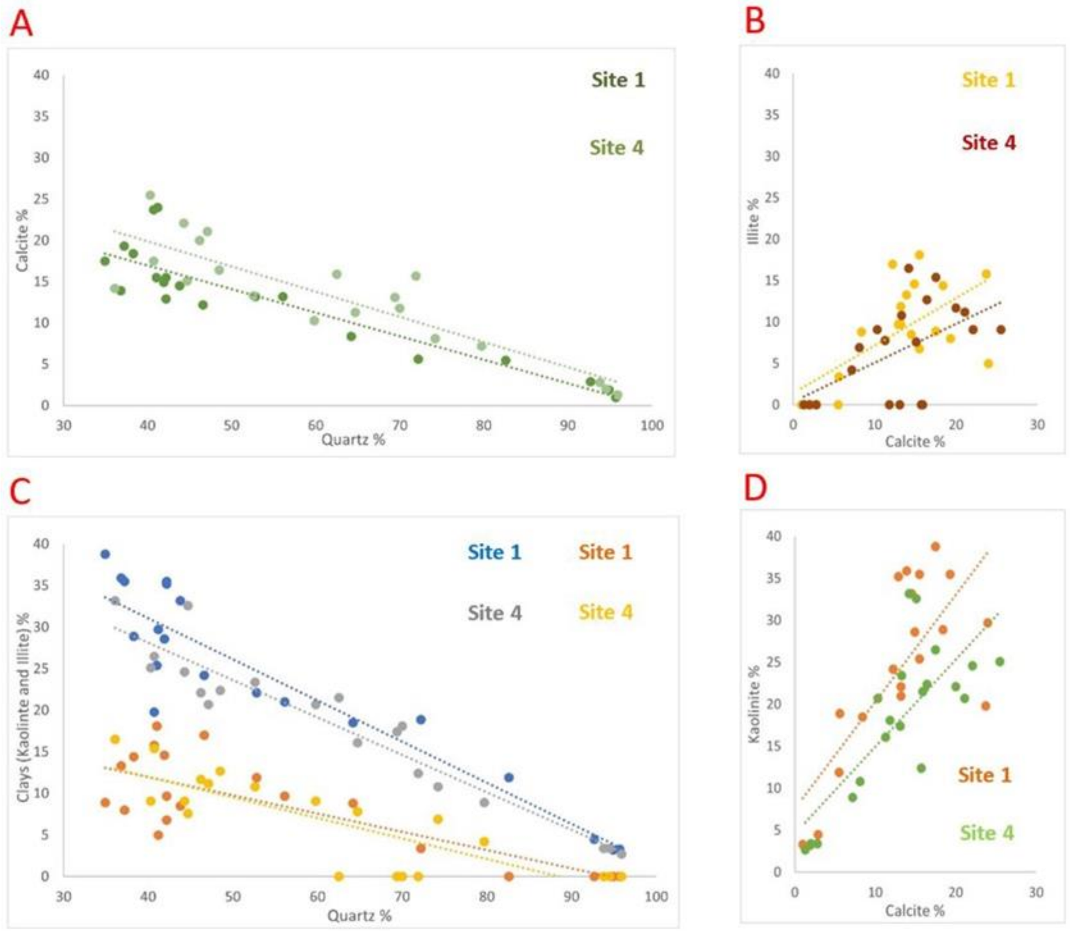

*Supplementary Figure 45 Scatter plots showing concentrations of minerals at sites 1(ARN) and 4 (JMI). **A:** Scatter plot quartz % vs calcite %. Dark green dots depict Site 1 and light green dots Site 4. **B:** Scatter plot calcite % versus illite %, yellow and brown dots are calcite % versus illite % for Sites 1 and 4. **C:** Scatter plot quartz % vs clays %, blue and grey dots are quartz % versus kaolinite % for site 1 and site 4 while orange and yellow dots are quartz % versus illite % for Site 1 and Site 4. **D:** Scatter plot of calcite % vs kaolinite %, orange and green dots are calcite % versus kaolinite % for Site1 and Site 4.*

The relationship between the mineralogical components can be visualized in scatter plots (Supplementary Figure 45) and quantified by the correlation coefficient for the different mineral phases (Supplementary Table 3). Both Site 1 and 4 show similar relationships, so they have been combined for statistical analysis. Interestingly, these virtually identical trends suggest they represent a regionally significant pattern. Quartz displays a strong negative relationship with both clays and calcite exhibiting an increased scatter for the lower quartz values (35 -50%) (Supplementary Figure 45A and (Supplementary Table 3). When calcite is compared to the two clay minerals (Supplementary Figure 45C), it shows a positive linear correlation with an increase of scatter towards the higher values, but these are not as strong as the negative relationships with quartz. The correlation between the calcite and clay fractions suggests they are coupled (Supplementary Figure 45B&D), whereas the inverse linear trend between the quartz and the other components suggests a dilution relationship. Since dune sands were present in the area from about 500 ka[36], the most straightforward interpretation is that the influx of the fine sand fraction varied, diluting the background sedimentation of clay and calcite. These influxes of sand are obvious in

the lower 1/3 part of both sections, whereas in the upper 2/3 of both sections there is fairly constant sedimentation of quartz, clays and calcite.

Supplementary Table 3 Correlation coefficients of the relationship between the mineral phases for Site 1 (ARN) and Site 4 (JMI). The colour scale is from red-orange, negative relationships, to yellow-green for positive

	Quartz	*Calcite*	*Kaolinite*	*Illite*
Quartz	1			
Calcite	-0.865	1		
Kaolinite	-0.936	0.719	1	
Illite	-0.81	0.582	0.644	1

5.3. Stratigraphic and palaeoenvironmental interpretation

Two sedimentological units with different paleoenvironmental interpretations are distinguished: gravels and playa sediments consisting of sandy silty clays. The gravels are poorly sorted and contain pebbles of the bedrock of up to 5 cm, suggesting a phase of significant precipitation in order to transport this coarse sediment fraction. The uncertainties on the luminescence age (68.8±5.0 ka, Table 1; Supplementary Figure 44B) cover the time from the end of MIS 5 to the onset of MIS 4. We propose that this material is most likely to have been deposited during the MIS5a humid phase, when numerous paleolakes were present in this region[37-40].

The gravels are considerably older than the overlying fine-grained sands and playa sediments. The sands are found interfingering with playa sediments, suggesting fluctuations between humid and arid conditions. The onset of this sedimentation occurs at 12.7±2.1 ka at Site 1 (ARN). The onset of sedimentation at Site 4 (JMI) is constrained by inverted but statistically indistinguishable ages of 17.1±1.7 and 15.5±1.5 ka. The apparent difference in age between the two sites may be an artefact of the uncertainties on individual age determinations or differences in their hydrology, driven by factors such as catchment size and elevation. After the period of fluctuating climate indicated by interdigitating sands and playa sediments, the sites appear to have become more humid as evidenced by the gradual increase in calcite that indicates the presence of water. Regardless of the source of this water, our data suggests the onset of wetter conditions started soon after ~16 ka (Site 4, JMI) to ~13 ka (Site 1, ARN), and that more humid conditions continue throughout the deposition of the overlying sediments in the section, albeit with some variation as indicated by the presence of sandy layers, mudcracks, and vugs in the sediment succession. However, the absence of freshwater molluscs, root traces or organic matter rich layers suggest that conditions were too dry for the establishment of permanent lakes.

Numerous paleolake deposits have been investigated in the region, though most are not comparable to those studied here as they correspond to the MIS 5 humid period e.g. Alathar, Al Wusta and Jubbah paleolakes[38,39,41]. Notwithstanding this, a shallow lake phase is documented at Jubbah at about 12.2 ka that is similar in age to the onset of playa sedimentation studied here and is followed by a second humid phase at about 6.6 ka[42]. However, additional luminescence research identified high levels of overdispersion and partial bleaching, which indicates these luminescence ages may reflect minimum ages[43]. In Tayma, a palaeolake is characterized by numerous phases of

carbonate sedimentation. Lake initiation and carbonate sedimentation commenced at 9.25 ka and is followed by deposition of calcareous verves signalling a wetter phase followed by gradual drying until 4.2 ka when lake sedimentation ceases[44]. Furthermore, nearer to the study area at Jebel Oraf, a paleolake exists during two main lake high stands at 8.5 ka and 7.3 ka[45]. The sediments consist of calcareous marls with lenses of organic matter and the presences of aquatic molluscs that indicate permanent water. Similar palaeolake sediments are found at Jebel Qattar in the Jubbah basin dating from about 8.7 to 8 ka (Crassard et al., 2013). These sediments contrast with those studied here, exhibiting more evidence of permanent lacustrine conditions and less evidence for dry episodes than we find. Given this it appears that the lakes in the Sahout region are more ephemeral, perhaps experiencing seasonal inundation.

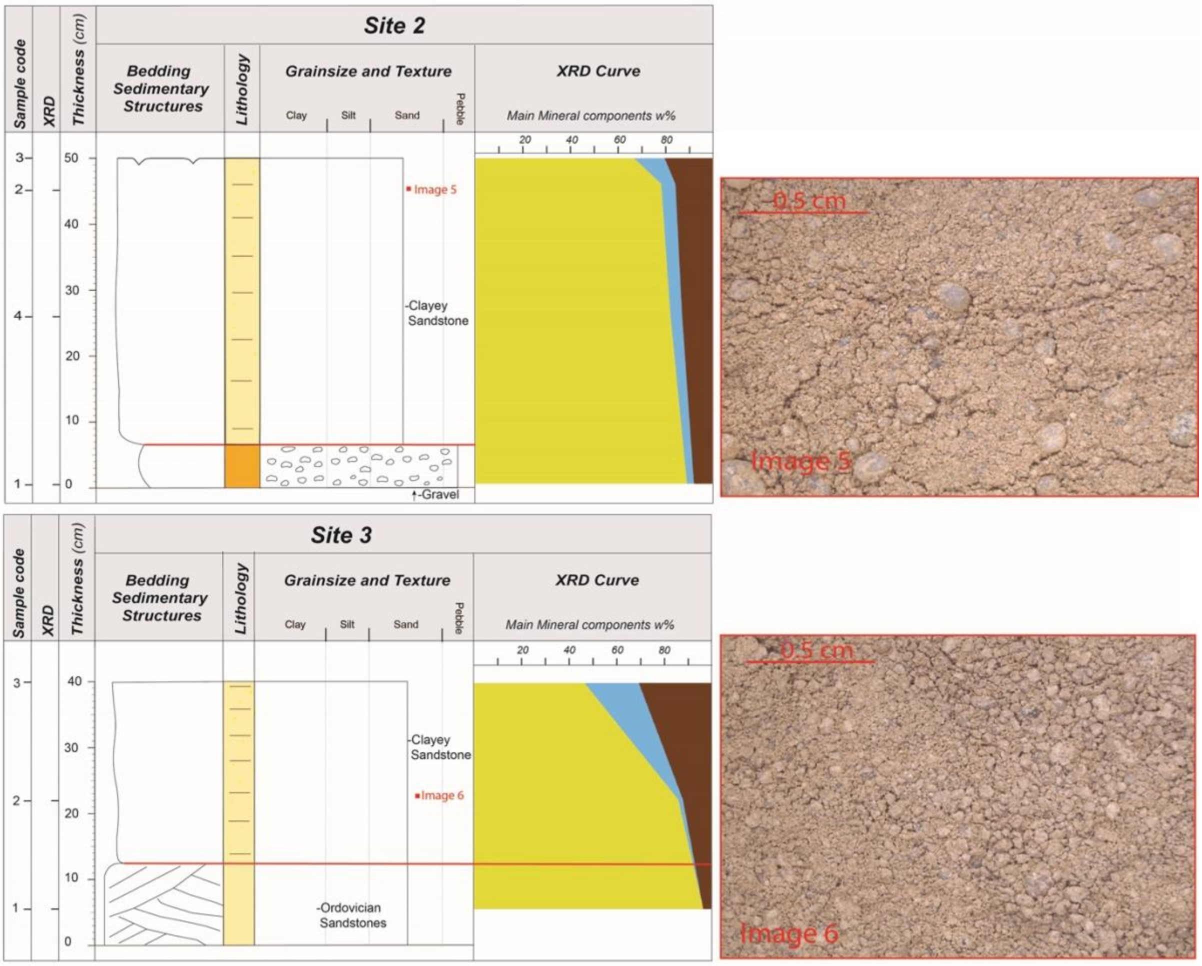

Supplementary Figure 46 Normalized staked area graphs and sedimentary sections of Site 2 and 3 with XRD analysis of the main mineral components and pictures of the sediment types.

Supplementary Note 6: Chronometric Dating

6.1. Luminescence Dating

6.1.1. Luminescence dating methodology

Luminescence dating is a group of dating techniques that allow determination of the time elapsed since sediment was last exposed to sunlight. Luminescence techniques are usually applied to sedimentary quartz (optically stimulated luminescence, OSL) and feldspar (infrared stimulated luminescence, IRSL), and have been applied successfully to a wide range of archaeological and Quaternary deposits[46-48].

After burial the sediment is exposed to a low level of radiation coming from both cosmic radiation (cosmic rays) and the decay of naturally-occurring radionuclides, principally uranium (U), thorium (Th), rubidium (Rb) and potassium (K). These radioisotopes, which are present in the surrounding sediment, emit alpha particles (α), beta particles (β) and gamma rays (γ). Most mineral crystals contain lattice defects or impurities where electrons become trapped when excited by ionizing radiation. The crystals store energy at a constant rate with the amount of stored energy being related to the time since deposition. Electrons can be removed from traps when a small amount of stimulating energy is subsequently applied, either from sunlight during transport or from an external light or thermal source, and this reduces the trapped electron population, a process referred to as bleaching, zeroing or resetting of the signal. In the case of OSL, photons are used to release the electrons from the traps. Once the electrons are evicted, they can be trapped again, or recombine in defects attractive to electrons (luminescence centres). The recombination of the electrons results in the emission of light, termed OSL. The intensity of the light signal emitted is proportional to the amount of electrons stored in the defects. This allows for the stored charge to be quantified. It can be used in combination with the data on the total dose rate received while buried (Gy/ka) to calculate the age since the last light or heat exposure following the equation[49]:

Age (ka) = Equivalent dose (D_e) (Gy) / Dose rate (Gy/ka)

The resulting luminescence age is obtained in calendar years prior to measurement – the datum used in this study is 2023. All uncertainties on quantities relating to the calculation of luminescence ages, including those on the ages themselves, are reported at 1 standard error unless otherwise stated.

6.1.2. Sample collection and preparation

Luminescence samples were collected by driving opaque tubes into cleaned section faces. Samples were prepared under subdued red-light conditions at Royal Holloway Luminescence Laboratory. Briefly, the outer, light-exposed portions of each sample were removed and used for environmental dose rate measurements. The remaining sediment was treated with hydrochloric acid (1M HCl) and hydrogen peroxide (H_2O_2) to remove carbonate and organic matter respectively. The samples were wet sieved to yield 180-210 μm sand, and quartz was extracted via density separation at 2.62 and 2.70 g/cm^3, and a subsequent HF acid etch (23M HF for 60 mins, followed by a 10M HCl

rinse). Etched material was re-sieved to remove partially dissolved grains. Sample SAH-L3-2 contained negligible sand-sized material. For this sample the 4-11 µm fraction was isolated by Stokes settling, and quartz was purified by prolonged (~1 week) immersion in silica-saturated fluorosilicic acid (H_2SiF_6.), followed immersion in 10M HCl overnight.

6.1.3. Luminescence measurements - Equipment

Luminescence measurements were performed using Risø TL/OSL-DA-15 or TL/OSL-DA-20 instruments, with the former system being fitted with a single-grain OSL attachment[50]. Stimulation of multi-grain aliquots was carried out using a blue (470 nm) light emitting diode (LED) array (nominal power density 33 mW/cm^2) and an infrared (875 nm) LED array (nominal power density 135 mW/cm^2), while single-grains were stimulated using a 10 mW Nd: YVO4 solid-state diode-pumped green laser (532 nm) focussed to yield a nominal power density of 50 W/cm^2[51]. Quartz luminescence emissions were detected using an Electron Tubes Ltd 9235QB photomultiplier tube via 7.5 mm of Hoya U-340. All irradiations were performed using a 1.48 GBq ^{90}Sr/^{90}Y beta source, calibrated relative to the National physical Laboratory, Teddington ^{60}Co gamma-source (Hotspot 800)[52]. For single-grain measurements, the effects of source heterogeneity[53] were circumvented by applying a grain position correction to each equivalent dose following[54]. Position corrected equivalent doses were used in all single-grain age calculations.

6.1.4. Equivalent dose determination

All equivalent doses were determined using multigrain aliquots (5 mm diameter sample area for sand-sized grains), with the exception of MIS-B-3, where insufficient material was available for this scale of analysis, and JMI8-4, where aliquots yielded equivalent doses ranging from ~10 to ~500 Gy. This latter result was interpreted as resulting from the incorporation of older grains into a younger sample. Equivalent dose (D_e) determinations were carried out using single-aliquot regenerative-dose protocol[55,56] (Supplementary Table 5). Regeneration doses were chosen to bracket the expected palaeodose. A number of additional regeneration points were also included to monitor the quality of the data generated: (1) a zero-dose point to measure the recuperation; (2) a repeat measurement of the initial regeneration dose to calculate the recycling ratio[55]; (3) a second repeat of the initial regeneration dose followed by a room temperature IR bleach and subsequent OSL measurement to calculate the IR depletion ratio[57]. We adopted a combination of 260 °C for 10 s for preheat 1 (PH1, the preheat before measurement of the natural or regenerated luminescence intensity) and 220 °C for 10 s for PH2 (the preheat before measurement of the test dose luminescence intensity) for multi-grain aliquot measurements and a 240 °C, 10 s PH1 followed by a 160 °C, 0 s PH2 for single-grain measurements (Supplementary Table 5). These measurement conditions were chosen based on previous experience of Nefud quartz and validated for these samples using dose recovery tests. Samples ARN-T1-3, JMI8-T1-4, MIS-B-1, MIS-B-2 and SAH-L3-1 yielded dose recovery ratios of 0.99±0.03, 1.06±0.08, 0.98±0.03, 1.02±0.03, 1.02±0.03 respectively, indicating that the measurement conditions employed were appropriate. The dose recovery test on sample JMI8-T1-4 was conducted on grains mounted in single grain discs, but with the data analysed using the "sum all grains" option in the Luminescence Analyst software, owing to the low intrinsic sensitivity of individual grain from that sample (see below). The dose response curves

were fitted with a saturating exponential plus linear function. D_e values for grains/aliquots were calculated by projecting the sensitivity-corrected natural luminescence intensity (L_n/T_n) onto the dose response curve, with an uncertainty term determined using a 1000 iteration Monte Carlo simulation. Curve fitting, D_e determination and Monte Carlo simulation were performed on multigrain aliquot data using version 4.31.9 of the Luminescence Analyst software[58] and on single-grain data using the function calSARED() provided in the R package numOSL[59,60].

Multigrain aliquots or individual grains were rejected where: (1) the natural signal from the aliquot/grain could not be distinguished from the background signal (determined using Luminescence Analyst "sig. >3 sigma above BG" rejection criterion in Analyst and a combination of "Tn below 3 sigma BG" and "RSE of Tn exceeds 10%" rejection criteria in calSARED(); (2) the recycling ratio differed from unity by >2 standard errors; (3) the sensitivity-corrected zero dose luminescence intensity was >5 % of the natural luminescence intensity; 4) the IR-depletion ratio was more than two standard errors below unity; (5) the natural luminescence signal (L_n/T_n) intercepts the dose response curve at a point where growth has ceased. An additional criterion was applied to single-grain data, and grains were rejected where: (6) the Figure of Merit of the growth curve exceeded 10%; In practice, for multigrain aliquot analysis only criteria 2 (recycling ratio) and 4 (IR-depletion ratio) were responsible for rejecting any data, with the majority of rejections being due to criterion 4. This pattern is consistent with other quartz luminescence data reported from Nefud samples[61, their Supplementary Table 5]. 7800 individual grains of JMI8-4 were analysed, with 18 grains displaying acceptable luminescence properties, a yield of ~0.002% (Supplementary Table 4). By contrast, measurement of 2400 grains of MIS-B-3 produced 20 acceptable grains, a yield of ~0.008%. Both yield values are very low in comparison with samples from elsewhere in the Nefud Desert measured using the same instrument[61], which we attribute to a combination of low intrinsic sensitivity combined with low absorbed doses. Equivalent dose distributions for single-grain datasets are presented in Supplementary Figure 47.

Supplementary Table 4 The number of grains measured, rejected and accepted during single-grain analysis of sample JMI8-4 and MIS-B-1. Numbered rejection criteria correspond to those defined in the text.

**Sample[a]**	**Measured (n)**	**Rejection criterion[b]**						***Accepted***	
		**1) Signal intensity**	**2) Recycling**	**3) Recuperation**	**4) IR depletion**	**5) Saturation**	**6) Figure of Merit**	**n**	**%**
JMI8-4	7800	7625	19	2	57	4	73	18	0.002
MIS-B-3	2400	2367	5	1	5	2	0	20	0.008

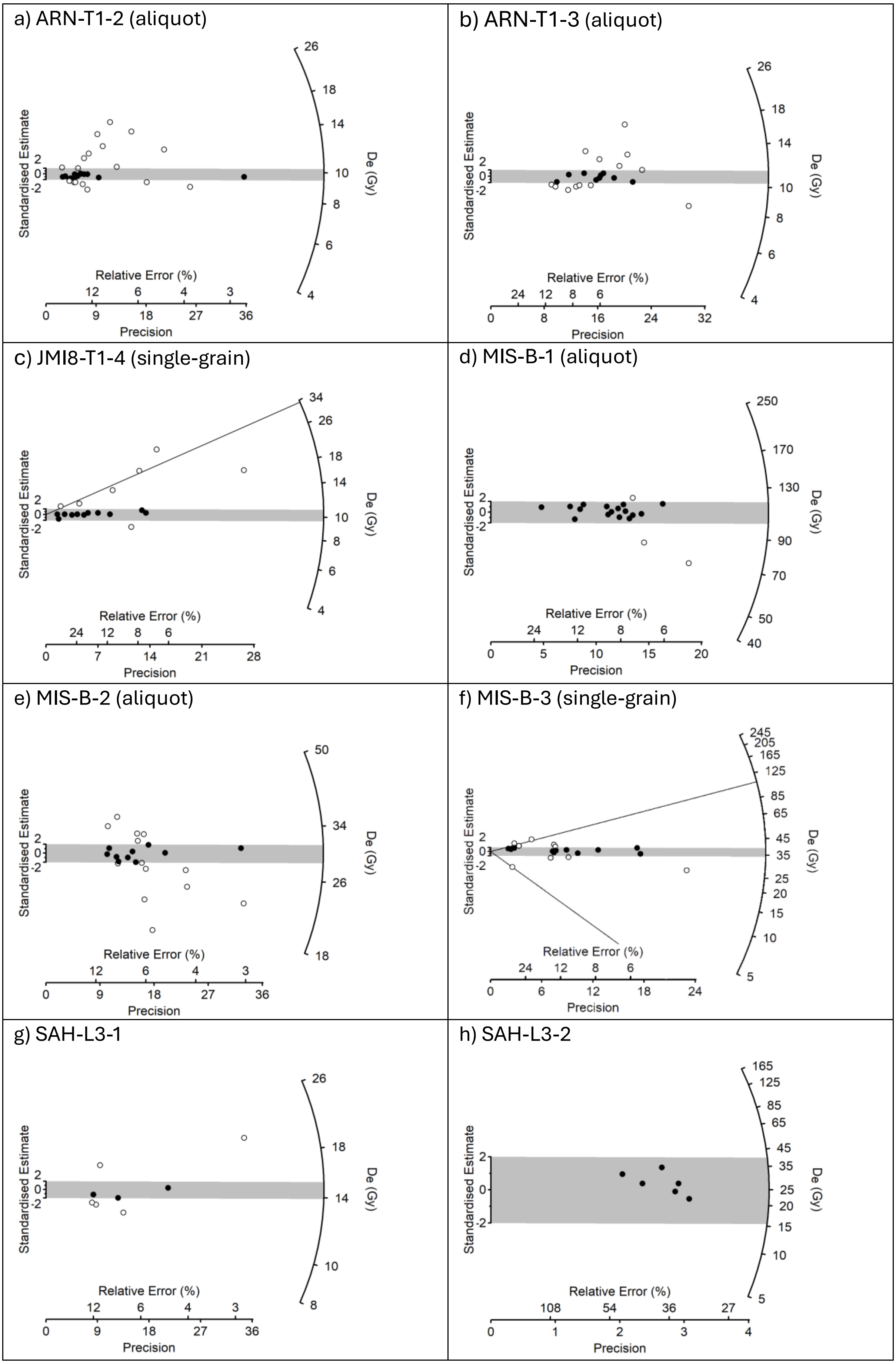
a) ARN-T1-2 (aliquot)
b) ARN-T1-3 (aliquot)
c) JMI8-T1-4 (single-grain)
d) MIS-B-1 (aliquot)
e) MIS-B-2 (aliquot)
f) MIS-B-3 (single-grain)
g) SAH-L3-1
h) SAH-L3-2
Standardised Estimate
De (Gy)
Relative Error (%)
Precision

Supplementary Figure 47. Equivalent dose distributions for samples all samples. The grey bars are centred on the Db for the sample and all points that lie within the bars are consistent (at 2 σ) with this dose. For samples JMI8-T1-4 and MIS-B-3 (panels c and f), the black lines represent non-dominant equivalent dose populations identified using the Finite Mixture Model.

6.1.5. Burial dose determination (Db)

The statistical models most frequently used to extract a single burial dose (D_b) from the distribution of measured D_e values are the central age model (CAM)[62], the Minimum Age Model (MAM)[63], and the Finite Mixture Model (FMM)[64]. The different models use different assumptions about the dataset so it is crucial to choose the correct one. CAM is designed for well-bleached samples which have experienced no post-depositional mixing. This model represents the ideal case, and calculates D_b from a weighted mean of the measured D_es. The MAM, as applied to single grain datasets, is used when the sample contains both fully and partially bleached grains. MAM fits a truncated normal distribution to log D_e values, with the truncation point giving D_b. Finally, FMM was developed to identify separate, fully bleached populations of grains within a single-grain dataset. This model is used when the sample is a mixture of different grain population, for example when mixing occurred after burial.

For all samples measured using multigrain aliquots, the burial dose was determined using the CAM. Because of the presence of high-dose (presumably intrusive) grains within the single-grain dataset for JMI8-T1-4, the FMM was used to identify the discrete grain populations. The FMM was fitted using overdispersion values of 10-26%, and an optimal fit at 20% overdispersion was determined from the maximum log-likelihood values and the Bayesian information criterion[65]. This yielded two populations, with 67% of grains in the most abundant (lower dose) population. The D_e value for this population was used to calculate the age for JMI8-T1-4. Similar analysis of MIS-B-3 yielded three populations at an optimum overdispersion of 25%. The dominant population (79% of grains yielding acceptable data) was used to calculate the age for MIS-B-3, though it is notable that application of the CAM (overdispersion = 72±12 %) yields a statistically indistinguishable D_e value.

6.1.6. Dose rate determination

The total dose rate for an HF etched quartz grain is a combination of beta and gamma radiation from radioisotopes in the sediment combined with a cosmic ray component. Beta dose rates were measured using a Risø GM-25-5 low-level beta counting system[66], using stainless steel and Volkagem loess standards[67]. Gamma dose rates were measured in the field using an EG&G Ortec digiDart-LF gamma-spectrometer, with the data being analysed using the threshold technique[68,69]. Dose rates were corrected for: (i) attenuation due to grain size; (ii) loss of beta dosed material due to HF etching[70] and (iii) moisture content[49]. A mean burial water content of 5±2 % was assumed for all samples. The 2σ uncertainty on water content encompasses very dry conditions (1%) and saturation for 20% of the burial period (8%), representing the full range of reasonable mean water content scenarios for these samples. Cosmic dose rate were calculated using site location (latitude, longitude and altitude) and present-day burial depths[71], assuming an overburden density of 1.85 g/cm^3.

6.1.7. Protocol and Results

Supplementary Table 5 the single-aliquot regenerative dose protocols applied to multigrain aliquot and single-grain of quartz in this study.

**Step**	**Quartz multigrain aliquot**	**Quartz Single-grain**
1	Give regenerative dose[1]	Give regenerative dose[1]
2	Preheat 1 (260 ^{0}C for 10 s)	Preheat 1 (240 ^{0}C for 10 s)
4	Blue diode stimulation (125 ^{0}C for 60 s)	Green laser stimulation (125 °C for 2 s)
5	Give test dose (~10 Gy)	Give test dose (~15 Gy)
6	Preheat 2 (220 ^{0}C for 10 s)	Preheat 2 (160 °C for 10 s)
8	Blue diode stimulation (125 ^{0}C for 60 s)	Green laser stimulation (125 °C for 2 s)
9	Blue diode bleach (280 ^{0}C for 100 s)	Blue diode bleach (280 ^{0}C for 100 s)
10	Return to step 1	Return to step 1

Supplementary Table 6 Dose rates, burial dose and ages. [1]Beta, gamma and total dose rates have been corrected for 5±2 % moisture content, while the beta dose rate has also been corrected for attenuation due to grain size and the effects of HF etching (except for SAH-L3-2, which was not etched). Cosmic dose rates have an assumed 10% uncertainty. [2]Single-grain measurements were made on samples JMI8-T1-4 and MIS-B-3, with D_b being calculated using the FMM. All other samples were measured as multigrain aliquots and D_b values calculated using the CAM.

Sample	Grain size (µm)	Depth (cm)	Dose rate (Gy/ka)[1]				D_b (Gy)	Age (ka)
			Beta	Gamma	Cosmic	Total		
ARN-T1-2	180-210	134	0.30±0.02	0.31±0.03	0.19±0.02	0.81±0.04	9.9±1.0	12.2±1.4
ARN-T1-3	180-210	124	0.32±0.02	0.33±0.03	0.20±0.02	0.85±0.04	10.9±0.8	12.8±1.1
JMI8-T1-4[2]	180-210	45	0.26±0.01	0.37±0.04	0.22±0.02	0.86±0.05	10.3±1.4[2]	12.0±1.8
MIS-B-1	180-210	185	0.66±0.04	0.75±0.08	0.18±0.02	1.59±0.09	109.5±5.3	68.8±5.0
MIS-B-2	180-210	170	0.86±0.05	0.88±0.09	0.19±0.02	1.93±0.10	29.9±2.3	15.5±1.5
MIS-B-3[2]	180-210	140	1.02±0.05	0.96±0.10	0.19±0.02	2.17±0.11	37.2±3.2[2]	17.1±1.7
SAH-L3-1	180-210	180	0.36±0.02	0.61±0.06	0.18±0.02	1.14±0.07	14.6±2.2	12.7±2.1
SAH-L3-2	4-11	60	1.86±0.13	1.26±0.13	0.21±0.02	3.33±0.18	25.2±2.9	7.6±1.0

6.2. Radiocarbon Dating

All samples were sent to the Centre for Applied Isotope Studies (CAIS) at the University of Georgia for radiocarbon dating. Charcoal samples were treated with 5% HCl at 80 °C for one hour, then they were washed with deionized water on a fiberglass filter and rinsed with diluted NaOH to remove possible contamination by humic acids. Samples were then treated with diluted HCl again, washed with deionized water and dried at 60 °C. For accelerator mass spectrometry analysis, the cleaned samples were combusted at 900 °C in evacuated / sealed ampoules in the presence of CuO.

The shell sample was treated with diluted HCl to remove any contamination from the surface. The washed and dried sample was treated in the vacuum with concentrated phosphoric acid to recover carbon dioxide (Supplementary Information 6).

The resulting carbon dioxide was cryogenically purified from the other reaction products and catalytically converted to graphite using the method of Vogel et al. (1984). Graphite $^{14}C/^{13}C$ ratios were measured using the CAIS 0.5 MeV accelerator mass spectrometer. The sample ratios were compared to the ratio measured from the Oxalic Acid I (NBS SRM 4990). The sample $^{13}C/^{12}C$ ratios were measured separately using a stable isotope ratio mass spectrometer and expressed as $\delta^{13}C$ with respect to PDB, with an error of less than 0.1‰.

Microcharcoal was collected during excavation, but appears to have a high incidence of contamination/movement, perhaps due to its small size. A charcoal piece retrieved from a hearth in ARN3 T2 and a piece of pear oyster retrieved from layer 3 at JMI7 T1 provided radiocarbon ages consistent with their stratigraphic position. A piece of ostrich eggshell retrieved from layer 8 at ARN3 T2 (the layer below the charcoal piece, Supplementary Figure 13) exceeds the age range of radiocarbon dating and was likely an ancient fragment that became incorporated in the sediment.

At JMI microcharcoal contamination was evident (Supplementary Table 7), a phenomenon that is quite common in desert environments and has also been reported from other excavations on the Arabian Peninsula[72].

*Supplementary Table 7 Radiocarbon ages from excavations at ARN and JMI. Uncalibrated dates are given in radiocarbon years before 1950 (years BP), using the ^{14}C half-life of 5568 years. The error is quoted as one standard deviation and reflects both statistical and experimental errors. The date has been corrected for isotope fractionation. Calibrated using OxCal calibration programme v4.4.4, which uses the IntCal20 calibration curve (Reimer et al., 2020), and 95.4% probability. *Calibrated using Marine20 (modelled ocean average) calibration curve [73]. Probability for individual calibrated age ranges is indicated in brackets. Radiocarbon ages highlighted in grey are also included in the main text.*

Lab code (UGAMS)	Layer	Material	$\delta^{13}C$,‰	pMC	^{14}C age years BP	calibrated date (calBP)	calibrated date (calBC/AD)
65277	ARN3, T2, Layer 8	eggshell	-10.24	<0.19	<49750	**Exceeds ^{14}C range**	
65278	ARN3, T2, Layer 7	Charcoal from hearth	-11.62	28.89±0.11	9970±30	**11613-11526 (16.2%)**<br>**11505-11421 (19.5%)**<br>**11412-11264 (59.7)**	9664-9577BC<br>9556-9472BC<br>9463-9315BC
69965*	JMI7, T1, Layer 3	shell	-3.36	79.57±0.22	1830±20	**2141-1776 (95.4%)**	192BC-172AD
65279	JMI7, T1, Layer 4	Micro charcoal	-11.37	88.55±0.25	980±20	**929-899 (35.6%)**<br>**871-796 (59.9%)**	1021-1051AD<br>1079-1154AD
65280	JMI7, T2, Layer 4	Micro charcoal	-11.93	89.15±0.31	920±30	**915-770 (88.8%)**<br>**762-742 (6.6%)**	1035-1181AD<br>1188-1210AD
65281	JMI8, T1, Layer 4	Micro charcoal	-12.15	99.41±0.29	50±25	**Invalid for calibration curve**	

Supplementary References

1 Guagnin, M. *et al.* Before the Holocene humid period: Life-sized camel engravings and early occupations on the southern edge of the Nefud Desert. *Archaeological Research in Asia* **36**, 100483, doi:https://doi.org/10.1016/j.ara.2023.100483 (2023).

2 Charloux, G., Guagnin, M., Alsharekh, A. & Petraglia, M. D. A Rock Art Tradition of Life-sized Naturalistic Engravings of Camels in Northern Arabia: New Insights on the Mobility of Neolithic populations in the Nafud Desert. *Antiquity Project Gallery* **96**, 1301-1309, doi:https://doi.org/10.15184/aqy.2022.95 (2022).

3 Guagnin, M. *et al.* Life-sized Neolithic camel sculptures in Arabia: A scientific assessment of the craftsmanship and age of the Camel Site reliefs. *Journal of Archaeological Science: Reports* **103165**, doi:https://doi.org/10.1016/j.jasrep.2021.103165 (2021).

4 Guagnin, M. *et al.* in *Revealing Cultural Landscapes in North-west Arabia (Supplement to Volume 51 of the Proceedings of the Seminar for Arabian Studies)* (eds Rebecca Foote, Maria Guagnin, Ingrid Périssé, & S. Karacic) 110-132 (Archaeopress, 2022).

5 Bednarik, R. G. & Khan, M. Scientific Studies of Saudi Arabian Rock Art. *Rock Art Research* **22**, 49-81 (2005).
6 Khan, M. *Prehistoric Rock Art of Northern Saudi Arabia*. (Ministry of Education, Department of Antiquities and Museums, 1993).
7 Khan, M. Rock Art of Saudi Arabia. *Arts* **2**, 447-475, doi:10.3390/arts2040447 (2013).
8 Guagnin, M. *et al.* Rock art imagery as a proxy for Holocene environmental change: A view from Shuwaymis, NW Saudi Arabia. *The Holocene* **26**, 1822-1834, doi:https://doi.org/10.1177/0959683616645949 (2016).
9 Guagnin, M. *et al.* An illustrated prehistory of the Jubbah oasis: Reconstructing Holocene occupation patterns in north-western Saudi Arabia from rock art and inscriptions. *Arabian Archaeology and Epigraphy* **28**, 138-152, doi:https://doi.org/10.1111/aae.12089 (2017).
10 Guagnin, M. *et al.* Rock art provides new evidence on the biogeography of kudu (Tragelaphus imberbis), wild dromedary, aurochs (Bos primigenius) and African wild ass (Equus africanus) in the early and middle Holocene of north-western Arabia. *Journal of Biogeography* **45**, 727-740, doi:10.1111/jbi.13165 (2018).
11 Groucutt, H. S. *et al.* Middle Palaeolithic raw material procurement and early stage reduction at Jubbah, Saudi Arabia. *Archaeological Research in Asia* **9**, 44-62 (2017).
12 Guagnin, M. *et al.* A tale of two hearth sites: Neolithic and intermittent mid to late Holocene occupations in the Jubbah oasis, northern Saudi Arabia. *Archaeological Research in Asia* **26**, 100278 (2021).
13 El-Gameel, K., Abdallah, S., Deevsalar, R. & Eliwa, H. New Insights into the Petrogenesis of Quaternary Peralkaline Volcanics, Jabal Al Abyad, Saudi Arabia. *Arabian Journal for Science and Engineering* **46**, 543-562 (2021).
14 Guagnin, M. *et al.* Before the Holocene humid period: Life-sized camel engravings and early occupations on the southern edge of the Nefud desert. *Archaeological Research in Asia* **36**, 100483 (2023).
15 Byrd, B. F. Vol. The Natufian culture in the Levant (eds François R Valla & Ofer Bar-Yosef) 245-264 (International Monographs in Prehistory, 1991).
16 Fujii, S. Settlement Pattern and Periodization of the Jordanian Badia Early PPNB: A Fresh Approach to the PPNA/PPNB Transition Issue in the Southern Levant. *Paléorient* **49**, 109-134, doi:https://doi.org/10.4000/paleorient.3582 (2024).
17 Crassard, R. *et al.* Beyond the Levant: first evidence of a Pre-Pottery Neolithic incursion into the Nefud Desert, Saudi Arabia. *PloS one* **8**, e68061 (2013).
18 Shea, J. J. *Stone Tools in the Paleolithic and Neolithic Near East: A Guide*. (Cambridge University Press, 2013).
19 Yashuv, T. & Grosman, L. in *Tracking the Neolithic in the Near East: Lithic perspectives on its origins, development and dispersals* (eds Y Nishiaki, O Maeda, & M Arimura) 17-32 (Sidestone Press, 2022).
20 Crassard, R. & Hilbert, Y. H. Bidirectional blade technology on naviform cores from northern Arabia: New evidence of Arabian-Levantine interactions in the Neolithic. *Arabian archaeology and epigraphy* **31**, 93-104 (2020).
21 Rosenberg, D. Serving meals making a home: the PPNA limestone vessel industry of the southern Levant and its importance to the Neolithic revolution. *Paléorient*, 23-32 (2008).

22 Hilbert, Y. H. *et al.* Traceological analysis of lithics from the Camel Site, al-Jawf, Saudi Arabia: an experimental approach to identifying mineral processing activities using silcrete tools. *Archaeological and Anthropological Sciences* **14**, 93 (2022).
23 Lucarini, G. *et al.* Plant, pigment, and bone processing in the Neolithic of northern Arabia–New evidence from Use-wear analysis of grinding tools at Jebel Oraf. *Plos one* **18**, e0291085 (2023).
24 Raad, D. R. & Makarewicz, C. A. Application of XRD and digital optical microscopy to investigate lapidary technologies in Pre-Pottery Neolithic societies. *Journal of Archaeological Science: Reports* **23**, 731-745 (2019).
25 Bar-Yosef Mayer, D. E. Towards a typology of stone beads in the Neolithic Levant. *Journal of Field Archaeology* **38**, 129-142 (2013).
26 Thuesen, M. B., Alarashi, H., Ruter, A. & Richter, T. Nascent craft specialization in the Pre-Pottery Neolithic A? Bead making at Shubayqa 6 (northeast Jordan). *PLOS ONE* **18**, e0292954, doi:10.1371/journal.pone.0292954 (2023).
27 Bar-Yosef Mayer, D. The lessons of Dentalium shells in the Levant. *Journal of the Israel Prehistoric Society* **40**, 219-228 (2010).
28 Bar-Yosef Mayer, D. Dentalium shells used by hunter-gatherers and pastoralists in the Levant. *Archaeofauna* **17**, 103-110 (2008).
29 Janjou, D. *et al.* (Ministry for Mineral Resources Geoscience Map GM-135, 1996).
30 Powers, R. W., Ramirez, L. F., Redmond, C. D. & Elberg, E. L. J. Geology of the Arabian Peninsula: Sedimentary geology of Saudi Arabia. 147 (1966).
31 Thomsen, K. J., Murray, A. S., Jain, M. & Bøtter-Jensen, L. Laboratory fading rates of various luminescence signals from feldspar-rich sediment extracts. *Radiation Measurements* **43**, 1474-1486, doi:https://doi.org/10.1016/j.radmeas.2008.06.002 (2008).
32 Gornitz, V. in *Encyclopedia of Paleoclimatology and Ancient Environments. Encyclopedia of Earth Sciences Series* (ed V. Gornitz) (Springer Dordrecht, 2009).
33 Poppe, L. J., Paskevich, V., Hathaway, J. & Blackwood, D. A Laboratory Manual for X-Ray Powder Diffraction. *US Geological Survey Open File Report* **1** (2001).
34 McHenry, L. J., Foerster, V. & Gebregiorgis, D. Paleolakes of Eastern Africa: Zeolites, Clay Minerals, and Climate. *Elements* **19**, 96-103, doi:10.2138/gselements.19.2.96 (2023).
35 Khrushcheva, M. O. *et al.* Taloe—Sedimentation in an Intermittent Lake (Russian Federation, Republic of Khakassia). *Minerals* **11**, 522 (2021).
36 Breeze, P. S. *et al.* Prehistory and palaeoenvironments of the western Nefud Desert, Saudi Arabia. *Archaeological Research in Asia* **10**, 1-16, doi:10.1016/j.ara.2017.02.002 (2017).
37 Rosenberg, T. M. *et al.* Middle and Late Pleistocene humid periods recorded in palaeolake deposits of the Nafud desert, Saudi Arabia. *Quaternary Science Reviews* **70**, 109-123, doi:10.1016/j.quascirev.2013.03.017 (2013).
38 Groucutt, H. S. *et al.* Homo sapiens in Arabia by 85,000 years ago. *Nature Ecology & Evolution* **2**, 800-809, doi:https://doi.org/10.1038/s41559-018-0518-2 (2018).

39 Stewart, M. *et al.* Human footprints provide snapshot of last interglacial ecology in the Arabian interior. *Science Advances* **6**, eaba8940, doi:https://www.science.org/doi/abs/10.1126/sciadv.aba8940 (2020).

40 Stimpson, C. M. *et al.* Middle Pleistocene vertebrate fossils from the Nefud Desert, Saudi Arabia: Implications for biogeography and palaeoecology. *Quaternary Science Reviews* **143**, 13-36, doi:10.1016/j.quascirev.2016.05.016 (2016).

41 Petraglia, M. D. *et al.* Hominin dispersal into the Nefud Desert and Middle palaeolithic settlement along the Jubbah Palaeolake, Northern Arabia. *PLoS One* **7**, e49840, doi:10.1371/journal.pone.0049840 (2012).

42 Hilbert, Y. H. *et al.* Epipalaeolithic occupation and palaeoenvironments of the southern Nefud desert, Saudi Arabia, during the Terminal Pleistocene and Early Holocene. *Journal of Archaeological Science* **50**, 460-474, doi:10.1016/j.jas.2014.07.023 (2014).

43 Clark-Balzan, L., Parton, A., Breeze, P. S., Groucutt, H. S. & Petraglia, M. D. Resolving problematic luminescence chronologies for carbonate- and evaporite-rich sediments spanning multiple humid periods in the Jubbah Basin, Saudi Arabia. *Quaternary Geochronology* **45**, 50-73, doi:10.1016/j.quageo.2017.06.002 (2018).

44 Neugebauer, I. *et al.* The unexpectedly short Holocene Humid Period in Northern Arabia. *Nature Communications Earth & Environment* **3**, 1-9, doi:https://doi.org/10.1038/s43247-022-00368-y (2022).

45 Guagnin, M. *et al.* The Holocene Humid Period in the Nefud Desert: Hunters and Herders in the Jebel Oraf Palaeolake Basin, Saudi Arabia. *Journal of Arid Environments* **178**, 104146, doi:https://doi.org/10.1016/j.jaridenv.2020.104146 (2020).

46 Duller, G. A. T. *Luminescence Dating : Guidelines on using luminescence dating in archaeology*. (English Heritage, 2008).

47 Wintle, A. G. Luminescence dating: where it has been and where it is going. *Boreas* **37**, 471-482, doi:https://doi.org/10.1111/j.1502-3885.2008.00059.x (2008).

48 Rhodes, E. J. Optically Stimulated Luminescence Dating of Sediments over the Past 200,000 Years. *Annual Review of Earth and Planetary Sciences* **39**, 461-488, doi:https://doi.org/10.1146/annurev-earth-040610-133425 (2011).

49 Aitken, M. J. *Thermoluminescence Dating* (Academic Press, 1985).

50 Bøtter-Jensen, L., Andersen, C. E., Duller, G. A. T. & Murray, A. S. Developments in radiation, stimulation and observation facilities in luminescence measurements. *Radiation Measurements* **37**, 535-541, doi:https://doi.org/10.1016/S1350-4487(03)00020-9 (2003).

51 Duller, G. A. T., Bøtter-Jensen, L., Murray, A. S. & Truscott, A. J. Single grain laser luminescence (SGLL) measurements using a novel automated reader. *Nuclear Instruments and Methods in Physics Research Section B: Beam Interactions with Materials and Atoms* **155**, 506-514, doi:https://doi.org/10.1016/S0168-583X(99)00488-7 (1999).

52 Armitage, S. J. & Bailey, R. M. The measured dependence of laboratory beta dose rates on sample grain size. *Radiation Measurements* **39**, 123-127, doi:https://doi.org/10.1016/j.radmeas.2004.06.008 (2005).

53 Ballarini, M., Wintle, A. G. & Wallinga, J. Spatial variation of dose rate from beta sources as measured using single grains. *Ancient TL* **24**, 1-7 (2006).
54 Armitage, S. J. *et al.* The Southern Route "Out of Africa": Evidence for an Early Expansion of Modern Humans into Arabia. *Science* **331**, 453-456, doi:doi:10.1126/science.1199113 (2011).
55 Murray, A. S. & Wintle, A. G. Luminescence dating of quartz using an improved single-aliquot regenerative-dose protocol. *Radiation Measurements* **32**, 57-73, doi:https://doi.org/10.1016/S1350-4487(99)00253-X (2000).
56 Murray, A. S. & Wintle, A. G. The single aliquot regenerative dose protocol: potential for improvements in reliability. *Radiation Measurements* **37**, 377-381, doi:https://doi.org/10.1016/S1350-4487(03)00053-2 (2003).
57 Duller, G. A. T. Distinguishing quartz and feldspar in single grain luminescence measurements. *Radiation measurements* **37**, 161-165 (2003).
58 Duller, G. A. T. Assessing the error on equivalent dose estimates derived from single aliquot regenerative dose measurements. *Ancient TL* **25**, 15-24 (2007).
59 Peng, J., Dong, Z., Han, F., Long, H. & Xiangjun, L. R package numOSL: numeric routines for optically stimulated luminescence dating. *Ancient TL* **31**, 41-48 (2013).
60 Peng, J. & Li, B. Single-aliquot Regenerative-Dose (SAR) and Standardised Growth Curve (SGC) Equivalent Dose Determination in a Batch Model Using the R Package 'numOSL'. *Ancient TL* **35** (2017).
61 Groucutt, H. S. *et al.* Multiple hominin dispersals into Southwest Asia over the past 400,000 years. *Nature* **597**, 376-380, doi:https://doi.org/10.1038/s41586-021-03863-y (2021).
62 Galbraith, R. F., Roberts, R. G., Laslett, G. M., Yoshida, H. & Olley, J. M. Optical Dating of Single and Multiple Grains of Quartz from Jinmium Rock Shelter, Northern Australia: Part I, Experimental Design and Statistical Models. *Archaeometry* **41**, 339-364, doi:https://doi.org/10.1111/j.1475-4754.1999.tb00987.x (1999).
63 Galbraith, R. F. & Laslett, G. M. Statistical models for mixed fission track ages. *Nuclear Tracks and Radiation Measurements* **21**, 459-470, doi:https://doi.org/10.1016/1359-0189(93)90185-C (1993).
64 Galbraith, R. F. & Green, P. F. Estimating the Component Ages in a Finite Mixture. *Nuclear Tracks and Radiation Measurements* **17**, 197-206 (1990).
65 Galbraith, R. F. & Roberts, R. G. Statistical aspects of equivalent dose and error calculation and display in OSL dating: An overview and some recommendations. *Quaternary Geochronology* **11**, 1-27, doi:https://doi.org/10.1016/j.quageo.2012.04.020 (2012).
66 Bøtter-Jensen, L. & Mejdahl, V. Assessment of beta dose-rate using a GM multicounter system. *International Journal of Radiation Applications and Instrumentation. Part D. Nuclear Tracks and Radiation Measurements* **14**, 187-191, doi:https://doi.org/10.1016/1359-0189(88)90062-3 (1988).
67 De Corte, F. *et al.* Preparation and characterization of loess sediment for use as a reference material in the annual radiation dose determination for luminescence dating. *Journal of Radioanalytical and Nuclear Chemistry* **272**, 311-319, doi:10.1007/s10967-007-0522-5 (2007).

68 Mercier, N. & Falguères, C. Field gamma dose-rate measurement with a NaI (Tl) detector: re-evaluation of the "threshold" technique. *Ancient TL* **25**, 1-4 (2007).
69 Duval, M. & Arnold, L. J. Field gamma dose-rate assessment in natural sedimentary contexts using LaBr3(Ce) and NaI(Tl) probes: A comparison between the "threshold" and "windows" techniques. *Applied Radiation and Isotopes* **74**, 36-45, doi:https://doi.org/10.1016/j.apradiso.2012.12.006 (2013).
70 Bell, W. T. Attenuation factors for the absorbed radiation dose in quartz inclusions for thermoluminescence dating. *Ancient TL* **8**, 1-12 (1979).
71 Prescott, J. R. & Hutton, J. T. Cosmic ray contributions to dose rates for luminescence and ESR dating: Large depths and long-term time variations. *Radiation Measurements* **23**, 497-500, doi:https://doi.org/10.1016/1350-4487(94)90086-8 (1994).
72 Purdue, L. *et al.* Geoarchaeology of Holocene oasis formation, hydro-agricultural management and climate change in Masafi, southeast Arabia (UAE). *Quaternary Research* **92**, 109-132, doi:10.1017/qua.2018.142 (2019).
73 Heaton, T. J. *et al.* Marine20—The Marine Radiocarbon Age Calibration Curve (0–55,000 cal BP). *Radiocarbon* **62**, 779-820, doi:10.1017/RDC.2020.68 (2020).
