# Supplementary material for: Monumental rock art illustrates that humans thrived in the Arabian Desert during the Pleistocene-Holocene transition
Source: Nat Commun. 2025 Sep 30;16:8249. doi: 10.1038/s41467-025-63417-y (PMC12485027; doi:10.1038/s41467-025-63417-y)
Supplement: Supplementary file 2 — Description of Additional Supplementary Files [file 41467_2025_63417_MOESM2_ESM.pdf]

Description of Additional Supplementary Files

Supplementary Data 1

List of rock art panels recorded at Jebel Arnaan (ARN), Jebel Misma (JMI) and Jebel Mleiha (MLH). Age assessment based on style, varnish and superimposition.

Supplementary Data 2

Breakdown of lithics by layer and material for Jebel Arnaan excavations at ARN3 T1 and T2. Note Layers 1-3 at ARN3 T1 were disturbed by the amateur enthusiast.

Supplementary Data 3

Breakdown of lithics by layer and material for Jebel Misma excavations at JMI7 and JMI8.
