# Supplementary material for: Monumental rock art illustrates that humans thrived in the Arabian Desert during the Pleistocene-Holocene transition
Source: Nat Commun. 2025 Sep 30;16:8249. doi: 10.1038/s41467-025-63417-y (PMC12485027; doi:10.1038/s41467-025-63417-y)
Supplement: Supplementary file 6 — Reporting Summary [file 41467_2025_63417_MOESM6_ESM.pdf]

nature portfolio

Corresponding author(s): Maria Guagnin, Ceri Shipton

Last updated by author(s): Jul 31, 2025

Reporting Summary

Nature Portfolio wishes to improve the reproducibility of the work that we publish. This form provides structure for consistency and transparency in reporting. For further information on Nature Portfolio policies, see our Editorial Policies and the Editorial Policy Checklist.

Statistics

For all statistical analyses, confirm that the following items are present in the figure legend, table legend, main text, or Methods section.

n/a	Confirmed	
☐	☒	The exact sample size (*n*) for each experimental group/condition, given as a discrete number and unit of measurement
☐	☒	A statement on whether measurements were taken from distinct samples or whether the same sample was measured repeatedly
☐	☒	The statistical test(s) used AND whether they are one- or two-sided<br>*Only common tests should be described solely by name; describe more complex techniques in the Methods section.*
☒	☐	A description of all covariates tested
☒	☐	A description of any assumptions or corrections, such as tests of normality and adjustment for multiple comparisons
☐	☒	A full description of the statistical parameters including central tendency (e.g. means) or other basic estimates (e.g. regression coefficient) AND variation (e.g. standard deviation) or associated estimates of uncertainty (e.g. confidence intervals)
☐	☒	For null hypothesis testing, the test statistic (e.g. *F*, *t*, *r*) with confidence intervals, effect sizes, degrees of freedom and *P* value noted<br>*Give P values as exact values whenever suitable.*
☐	☒	For Bayesian analysis, information on the choice of priors and Markov chain Monte Carlo settings
☒	☐	For hierarchical and complex designs, identification of the appropriate level for tests and full reporting of outcomes
☒	☐	Estimates of effect sizes (e.g. Cohen's *d*, Pearson's *r*), indicating how they were calculated

Our web collection on statistics for biologists contains articles on many of the points above.

Software and code

Policy information about availability of computer code

Data collection	Microsoft Excel v.365
Data analysis	OxCal v.4.4 for Table 2, Figure 6, and Supplementary Table 9; Microsoft Excel v.365 for Supplementary Table 2 and Supplementary Table 4; Diffrac.Eva software for Supplementary Figure 42, Supplementary Figure 43, and Supplementary Figure 44; Luminescence Analyst for Figure 6 and Supplementary Table 8

For manuscripts utilizing custom algorithms or software that are central to the research but not yet described in published literature, software must be made available to editors and reviewers. We strongly encourage code deposition in a community repository (e.g. GitHub). See the Nature Portfolio guidelines for submitting code & software for further information.

Data

Policy information about availability of data

All manuscripts must include a data availability statement. This statement should provide the following information, where applicable:

- Accession codes, unique identifiers, or web links for publicly available datasets
- A description of any restrictions on data availability
- For clinical datasets or third party data, please ensure that the statement adheres to our policy

All data used in this study are available in the Supplementary Information files. All data used to support our results was generated in our research. Sediment samples from playa deposits are stored at King Abdullah University for Science and Technology, Physical Science and Engineering Division. Artefacts recovered

during excavations will be returned to, and be stored by the Heritage Commission, Ministry of Culture, Saudi Arabia in 2025. Access can be negotiated with the Heritage Commission (faljibrin@moc.gov.sa).

Research involving human participants, their data, or biological material

Policy information about studies with human participants or human data. See also policy information about sex, gender (identity/presentation), and sexual orientation and race, ethnicity and racism.

Reporting on sex and gender	N/A
Reporting on race, ethnicity, or other socially relevant groupings	N/A
Population characteristics	N/A
Recruitment	N/A
Ethics oversight	N/A

Note that full information on the approval of the study protocol must also be provided in the manuscript.

Field-specific reporting

Please select the one below that is the best fit for your research. If you are not sure, read the appropriate sections before making your selection.

☐ Life sciences ☐ Behavioural & social sciences ☒ Ecological, evolutionary & environmental sciences

For a reference copy of the document with all sections, see nature.com/documents/nr-reporting-summary-flat.pdf

Ecological, evolutionary & environmental sciences study design

All studies must disclose on these points even when the disclosure is negative.

Study description	Archaeological survey and excavation, coupled with palaeoenvironmental survey and analysis of ancient lake sediments.
Research sample	Four excavated archaeological trenches (total of 7 square metres) excavated to a depth of 190cm/125cm/45cm/70cm respectively, and survey of surrounding landscape, following the topography of sandstone outcrops. Four palaeoenvironmental trenches excavated to a depth of 200cm/200cm/50cm/40cm respectively.
Sampling strategy	All archaeological sediment was sieved with 3mm mesh to retrieve small artefacts and bone fragments. All artefacts and bone fragments were examined.
Data collection	Pro forma excavation context and survey sheets in the field (paper forms and pen). Calliper measurements of stone artefacts in the laboratory. Visual assessment against comparative collections of fauna in the laboratory. XRF analysis of lake sediments.
Timing and spatial scale	Excavation in May 2023, data collection in the lab continued until July 2024
Data exclusions	Two palaeoenvironmental excavation trenches did not contain sufficient sediment for analysis and luminescence dating (Site 3 and Site 3, Supplementary Figure 44)
Reproducibility	All typologically distinctive artefacts are shown in the figures of the Manuscript and Supplementary Notes to ensure reproducibility of results.
Randomization	Samples were grouped based on the trench they were collected from and based on stratigraphic position.
Blinding	Blinding was not used as most researchers were involved in the fieldwork as well as the laboratory data collection.

Did the study involve field work? ☒ Yes ☐ No

Field work, collection and transport

Field conditions	Warm and dry desert environment
Location	Sahout region: Lat 27.339036° Long 39.851160°

Access & import/export	Excavations and export of finds were carried out with permission of the Saudi Heritage Commission and according to Saudi, EU and UK law; permission was grated on 16 May 2023 .
Disturbance	Excavations were back-filled upon completion.

Reporting for specific materials, systems and methods

We require information from authors about some types of materials, experimental systems and methods used in many studies. Here, indicate whether each material, system or method listed is relevant to your study. If you are not sure if a list item applies to your research, read the appropriate section before selecting a response.

Materials & experimental systems

n/a	Involved in the study
☒	☐ Antibodies
☒	☐ Eukaryotic cell lines
☐	☒ Palaeontology and archaeology
☒	☐ Animals and other organisms
☒	☐ Clinical data
☒	☐ Dual use research of concern
☒	☐ Plants

Methods

n/a	Involved in the study
☒	☐ ChIP-seq
☒	☐ Flow cytometry
☒	☐ MRI-based neuroimaging

Palaeontology and Archaeology

Specimen provenance	Saudi Arabia, Heritage Commission, Saudi Ministry of Culture
Specimen deposition	Finds are temporarily housed at the University College London, but long-term curation will be at the Heritage Commission, Saudi Ministry of Culture.
Dating methods	Dating methods are described in detail in Supplementary Notes 6.1 and 6.2 - Radiocarbon dating and Luminescence dating

☒ Tick this box to confirm that the raw and calibrated dates are available in the paper or in Supplementary Information.

Ethics oversight	Saudi Heritage Commission, Ministry of Culture

Note that full information on the approval of the study protocol must also be provided in the manuscript.

Plants

Seed stocks	N/A
Novel plant genotypes	N/A
Authentication	N/A
